# The KdmB-EcoA-RpdA-SntB (KERS) chromatin regulatory complex controls development, secondary metabolism and pathogenicity in *Aspergillus flavus*

**DOI:** 10.1016/j.fgb.2023.103836

**Published:** 2023-09-03

**Authors:** Betim Karahoda, Brandon T. Pfannenstiel, Özlem Sarikaya-Bayram, Zhiqiang Dong, Koon Ho Wong, Alastair B. Fleming, Nancy P. Keller, Özgür Bayram

**Affiliations:** a Biology Department, Maynooth University, Maynooth, Co. Kildare, Ireland; b Department of Medical Microbiology and Immunology, University of Wisconsin, Madison, USA; c Faculty of Health Sciences, University of Macau, Macau; d Institute of Translational Medicine, University of Macau, Macau; e Ministry of Education Frontiers Science Center for Precision Oncology, University of Macau, Macau; f Department of Microbiology, Moyne Institute of Preventive Medicine, Trinity College Dublin, Dublin, Ireland

## Abstract

The filamentous fungus *Aspergillus flavus* is a plant and human pathogen predominantly found in the soil as spores or sclerotia and is capable of producing various secondary metabolites (SM) such as the carcinogenic mycotoxin aflatoxin. Recently, we have discovered a novel nuclear chromatin binding complex (KERS) that contains the JARID1-type histone demethylase KdmB, a putative cohesion acetyl transferase EcoA, a class I type histone deacetylase RpdA and the PHD ring finger reader protein SntB in the model filamentous fungus *Aspergillus nidulans*. Here, we show the presence of the KERS complex in *A. flavus* by immunoprecipitation-coupled mass spectrometry and constructed *kdmB*Δ and *rpdA*Δ strains to study their roles in fungal development, SM production and histone post-translational modifications (HPTMs). We found that KdmB and RpdA couple the regulation of SM gene clusters with fungal light-responses and HPTMs. KdmB and RpdA have opposing roles in light-induced asexual conidiation, while both factors are positive regulators of sclerotia development through the *nsdC* and *nsdD* pathway. KdmB and RpdA are essential for the productions of aflatoxin (similar to findings for SntB) as well as cyclopiazonic acid, ditryptophenaline and leporin B through controlling the respective SM biosynthetic gene clusters. We further show that both KdmB and RpdA regulate H3K4me3 and H3K9me3 levels, while RpdA also acts on H3K14ac levels in nuclear extracts. Therefore, the chromatin modifiers KdmB and RpdA of the KERS complex are key regulators for fungal development and SM metabolism in *A. flavus*.

## 1. Introduction

The genus *Aspergillus* contains more than 300 biotechnologically, medically and agriculturally relevant species including the model filamentous fungus *Aspergillus nidulans* and the opportunistic human pathogen *A. fumigatus* and the notorious toxin producing species *A. flavus* which is both a plant and human pathogen (Amaike and Keller, 2009; Hedayati et al., 2007). *A. flavus* can contaminate oil-rich seeds such as corn, maize or peanuts during pre or post-harvest with the carcinogenic mycotoxin, aflatoxin (AF) (Mitchell et al., 2016). Every year, approximately $1 billion of economic loss is caused by AF contamination in the US alone (Amare and Keller, 2014). Furthermore, it is estimated that around 5 billion people are susceptible to AF toxicoses worldwide (Faustinelli et al., 2016). Once AF is metabolised by animals, reactive epoxide intermediates, which are mutagenic, are generated and may affect the tumour suppressor gene *p53*, leading to liver cancer (Hsu et al., 1991).

The *A. flavus* genome (37 Mb) encodes approximately 12,000 genes on 8 chromosomes. Depending on the isolates, *A. flavus* contains between 78 and 85 secondary metabolite (SM) gene clusters, including the AF cluster (Drott et al., 2021; Rokas et al., 2007; Skerker et al., 2021). Some of these SM clusters have been shown to produce harmful (e.g. aflatoxin, aflatrem, aflavarin, cyclopiazonic acid, piperazine) and beneficial (e.g. the cosmetic product kojic acid) compounds (Gallagher and Wilson, 1979; Parrish et al., 1966). Among these SM clusters, the AF cluster has been the best characterized (Fernandes et al., 1998; Yu, 2012). Many SM global regulators have been found to regulate the AF cluster including the Velvet complex members LaeA, VeA and VelB (Chang et al., 2013) as well as several histone modifying proteins (Pfannenstiel et al., 2018; Yang et al., 2022).

Histone posttranslational modifications (PTMs) are important for the regulation of eukaryotic gene transcription (Klemm et al., 2019; Weaver et al., 2018). Enzymes that catalyse or remove histone modifications are collectively named as ‘writers’ and ‘erasers’, respectively. Proteins that bind to these histone modifications and facilitate the function of writers and erasers are called ‘readers’ (Gillette and Hill, 2015).

In fungi, histone PTMs regulate numerous key fungal processes including SM production, development and virulence (Bok et al., 2009; Keller, 2018; Ramirez-Prado et al., 2018; Reyes-Dominguez et al., 2010; Yang et al., 2022). Histone acetylation and deacetylation play opposing roles in SM regulation. It was shown that histone acetylation is required for production of AF and sterigmatocystin (ST) in *A. flavus* and *A. nidulans* (Roze et al., 2007; Soukup et al., 2012; Sun et al., 2021). Furthermore, two HAT enzymes, GcnE, (required for histone H3 lysine-9 (H3K9ac) and lysine-14 acetylation (H3K14ac)) and EsaA (involved in histone H4 lysine-12 acetylation (H4K12ac)) were shown to be essential for activation of SMs in *A. nidulans* (Nutzmann et al., 2011; Soukup et al., 2012). Several regulators of histone acetylation or deacetylation have been shown to influence pathogenicity and aflatoxin production in *A. flavus* (Lan et al., 2016; Wen et al., 2022). Use of HDAC inhibitors such as suberoylanilide hyrdoxamic acid (SAHA) or trichostatin A (TSA) in fungal cultures have proven to be an effective strategy to activate cryptic gene clusters and produce novel SMs (Henrikson et al., 2009; Shwab et al., 2007). The class I histone deacetylase HDAC RpdA (Rpd3 ortholog in *Saccharomyces cerevisiae*) is essential for growth and development in *A. nidulans*, *A. fumigatus* and *Cochliobolus carbonum* (Graessle et al., 2000; Tribus et al., 2010). Interestingly, reduction of RpdA expression repressed ST production while promoting expression of several cryptic metabolites in *A. nidulans* (Albright et al., 2015). Therefore, in general, histone acetylation by histone acetyltransferases (HATs) positively regulates production of SMs, whereas histone deacetylases (HDACs) have negative impacts (Shwab et al., 2007; Soukup et al., 2012).

The role of histone methylation in the regulation of SM gene expression is less clear. Methylation of H3 lysine-9 (H3K9me) was shown to be important for heterochromatin formation and for repression of ST in *A. nidulans* (Gacek-Matthews et al., 2016; Reyes-Dominguez et al., 2010), although this modification is not found at most SM clusters in *A. fumigatus* (Colabardini et al., 2022). Interestingly, loss of the COMPASS complex member CclA, which is required for the activating PTM H3 lysine-4 methylation (H3K4me2/3), led to the production of several cryptic metabolites in *A. nidulans* (Bok et al., 2009). On the other hand, histone demethylation has also been implicated in SM regulation. In *A. nidulans*, two histone demethylases are involved in the production of SMs; KdmA, a histone H3K36me demethylase, was shown to be a regulator of primary and SM production (Gacek-Matthews et al., 2015), while KdmB, another Jumonji class histone H3K4me demethylase, was shown to be required for the positive regulation of SM (Gacek-Matthews et al., 2016).

Recently, a reader protein has also been shown to regulate SM production. SntB is a ring finger protein with bromo-adjacent homology (BAH), plant homeodomain (PHD) and Swi3-Ada2-N-CoR-TFIIIB (SANT) domains required for the production of AF and ST in *A. flavus and A. nidulans*, respectively (Pfannenstiel et al., 2018; Pfannenstiel et al., 2017). This protein was also shown to be required for sclerotia formation in *A. flavus* (Pfannenstiel et al., 2018). In *S. cerevisiae*, the yeast orthologue of SntB (Snt2) interacts with the HDAC Rpd3, and the lysine demethylase Ecm5 to mediate hydrogen peroxide oxidative stress responses (Baker et al., 2013). *Schizosaccharomyces pombe* Snt2 can also interact with the HDAC Rpd3 and the lysine demethylase Lid2 (homolog of KdmB) (Roguev et al., 2004). The function of Snt2 homologs were also studied in the model fungus *Neurospora crassa* and the plant pathogenic fungus *Fusarium oxysporum f.sp. melonis* where *snt2* deletion caused reduced conidiation and pathogenicity, respectively (Denisov et al., 2011; Pfannenstiel et al., 2018). Additionally, further PHD domain proteins were found to be involved in sporulation (conidiation) and sclerotia formation in *A. flavus* (Hu et al., 2018).

We have recently identified KdmB-EcoA-RpdA-SntB (KERS) chromatin regulatory complex in *A. nidulans* using protein pulldowns coupled with liquid chromatography-mass spectrometry (LC-MS) (Bayram et al., 2012; Karahoda et al., 2022). The KERS complex binds to promoter regions of more than 1,600 genes and control the expression of many regulatory genes for SM production and fungal development. Consequently, the KERS mutants have defects in asexual conidiation, sexual fruit body formations as well as the production of the mycotoxin ST.

Here, we present our findings on the identification and functional characterisation of the KERS complex in *A. flavus*. We show that, similar to *A. nidulans*, the *A. flavus* KERS complex consists of two erasers (KdmB and RpdA), one putative writer (the putative cohesin acetyl-transferase EcoA) as well as the reader protein SntB. Strikingly, we found that KdmB and RpdA regulate conidiation, sclerotia development, AF B_1_ production and virulence on peanuts and are required for wild-type expression of almost 80% of identified SM backbone genes, despite their differential roles on the chromatin – e.g. KdmB influence methylation of histone H3 at lysines 4 and 9, while RpdA is important for H3K14ac deacetylation.

## 2. Results

### 2.1. KdmB interacts with EcoA, RpdA and SntB to form a tetrameric demethylase complex

**Fig. 1.**
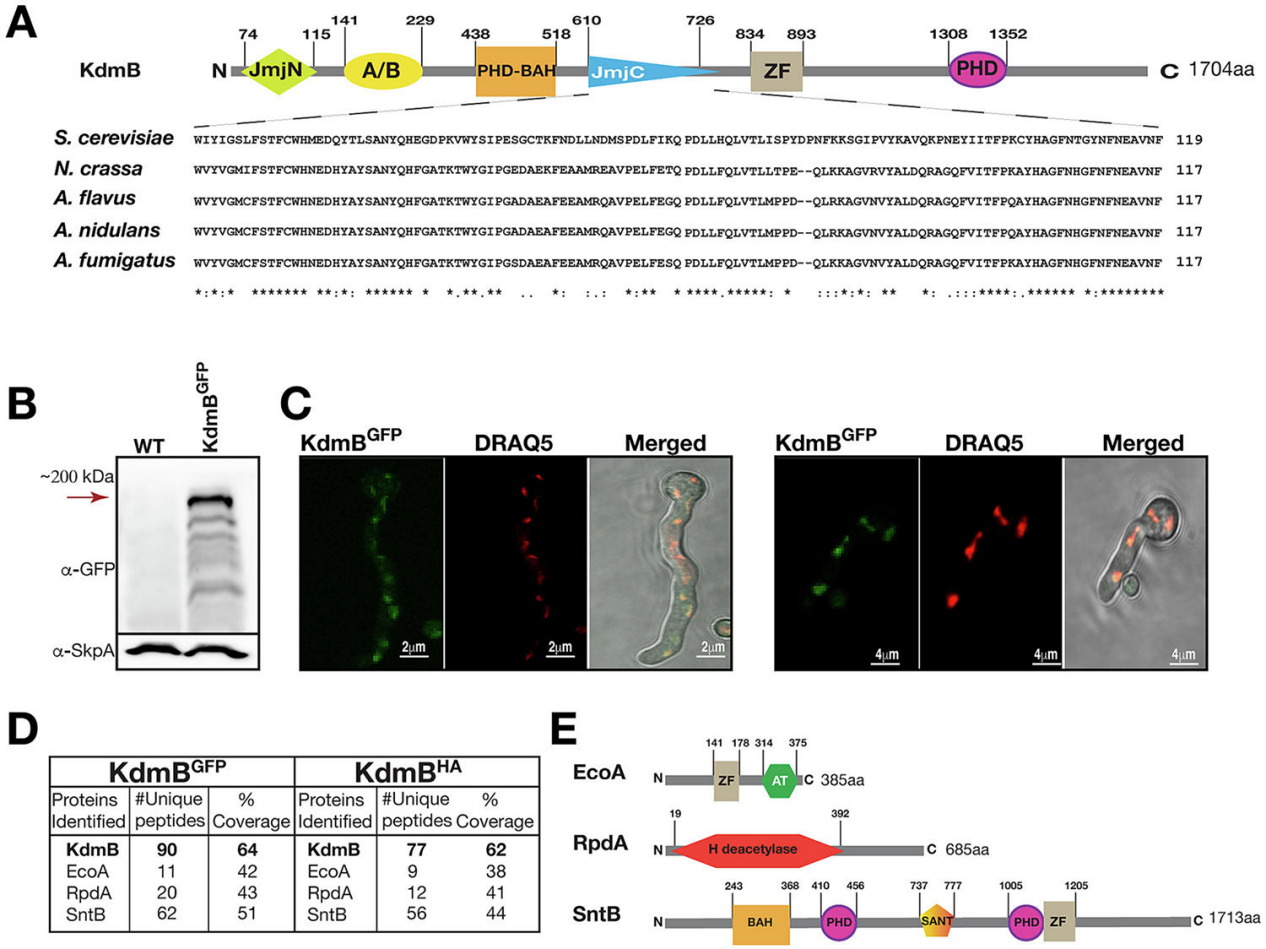
Identification of the KERS complex in Aspergillus flavus. (**A**) *A. flavus* KdmB domain architecture and alignment of Jumonji C (JmjC) domain with *S. cerevisiae* Jhd2, *Neurospora crassa* NCU01238, *A. nidulans* KdmB, and *A. fumigatus* AFUA_5G03430. (**B**) Protein expression of KdmB::GFP fusion protein assayed by a Western blot analysis. KdmB::GFP fusion immunoblot was prepared from crude extract of vegetative growth at 30 °C for 24 h. As a loading control, a subunit of SCF complex, SkpA was used. (**C**) Subcellular localization of KdmB expressed under native promoter. Nuclei staining was performed at the end of 16 h submerged growth by treating samples with 1:10,000 DRAQ5 dye for 30 min at room temperature. (**D**) LC-MS^2^ identification of KERS complex. Table shows the identified complex components with unique peptide numbers and total coverage. Demethylase KdmB interacts with putative acetyltransferase EcoA, histone deacetylase RpdA and Ring finger protein SntB to form the tetrameric KERS complex *in vivo*. Two biological replicates of KdmB::sGFP and KdmB::3xHA fusion strains and WT as negative control were immunoprecipitated and run in LC-MS^2^. The protein list obtained from the WT control was subtracted from KdmB::sGFP (Table S1) and KdmB::3xHA (Table S2) purifications to eliminate non-specific contaminants. (**E**) Domain architectures of multidomain KERS complex proteins. Like KdmB, SntB contains the histone binding plant homeodomain (PHD) which is uniquely involved in chromatin regulation.

The histone demethylase, KdmB, has been shown to control SM production in *A. nidulans* (Gacek-Matthews et al., 2016) and is essential for the formation of the KERS complex that controls key regulators in *A. nidulans* (Karahoda et al., 2022). We aimed to determine whether the KERS complex is conserved and whether KdmB is important for production of SM and pathogenicity in *A. flavus*. The *A. flavus* KdmB ortholog sequence (AFLA 006240) contains a Jumonji N domain at its N-terminus (between amino acids 74–115), an ARID/Bright domain (141–229), a plant homeodomain, a bromo adjacent homology (PHD-BAH) domain (438–518), a Jumonji C domain (610–726), a zinc finger domain (834–893) and a second PHD domain (1306–1352) at the C terminus with a total size of 1704 amino acids (Fig. 1A). The *kdmB* gene was endogenously expressed from its native locus as a fusion protein to green fluorescent protein (sGFP) (*kdmB::sgfp*) or human influenza hemagglutinin (HA) (*kdmB::3xha*). The full length (~200 kDa) KdmB^GFP^ fusion was clearly observed in an immunoblotting assay (Fig. 1B). The KdmB^GFP^ strain was used to investigate the intracellular localization of KdmB. GFP signals of KdmB overlapped with red-stained nuclei (DRAQ5) and presented a predominantly nuclear localization in vegetative cells under confocal microscopy (Fig. 1C).

In order to determine the interaction partners of KdmB (with 79% similarity to *A. nidulans* KdmB), separate HA and GFP immunoprecipitations were performed and and subjected to LC-MS/MS. The KdmB^GFP^ and KdmB^HA^ proteins recruited the same three interacting proteins in both HA and GFP immunoprecipitations (Fig. 1D); the cohesin acetyltransferase EcoA (yeast Eco1 ortholog), the histone deacetylase RpdA (yeast Rpd3) and the PHD domain ring finger protein SntB. These proteins are the top candidates in the pull down results with the highest number of unique peptides among all other peptides identified, suggesting that they are genuine interacting partners forming strong stable interactions (Fig. 1D).

Domain architectures of KERS members contain chromatin recognition and binding sites with catalytic domains involved in demethylation, acetylation and deacetylation (Fig. 1F). EcoA (with 83% similarity to *A. nidulans* EcoA) is the smallest member of the tetrameric complex comprising 385 amino acids. It contains a zinc finger domain (141–178) and an acetyl transferase (AT) (314–375) domain. The putative HDAC RpdA (with 78% similarity to *A. nidulans* RpdA) contains a deacetylase domain (19–392). The previously characterized SntB protein (69% similar to *A. nidulans* SntB) is composed of 1713 amino acids and contains a PHD-BAH domain (243–368), two PHD domains (410–456 and 1005–1205) and a SANT domain (737–777) named after common chromatin regulators Swi3-Ada2-N-CoR-TFIIIB of yeast (Ji and Arnot, 1997). The KERS complex members have domain architecture unique to chromatin modifier enzymes involved in catalytic regulation of histone PTMs. Taken together, our data show that KdmB interacts with three additional proteins EcoA, RpdA and SntB to establish a tetrameric nuclear KERS complex in *A. flavus*. Therefore, the formation KERS complex is conserved in *A. flavus*.

### 2.2. KdmB and RpdA are regulators of asexual conidiation

The KERS complex subunit SntB has been recently shown to be essential for sclerotia development in *A. flavus* (Pfannenstiel et al., 2018). In order to investigate the possible impact of other complex subunits in fungal development, deletion cassettes for *kdmB* (AFLA 006240), *ecoA* (AFLA 103370) and *rpdA* (AFLA 092360) were introduced into a WT *pyrG^−^* strain. *kdmB* and *rpdA* were successfully deleted in *A. flavus* (Fig. S1) generating four independent deletion strains for each gene. The successful deletion of *rpdA* was surprising considering that this gene is essential in *A. nidulans*, *A. fumigatus*, and *C. carbonum* (Tribus et al., 2010). On the other hand, no transformant was obtained for *ecoA* deletion despite many attempts, suggesting that the *ecoA* gene is essential for growth, similar to the situation in *A. nidulans* (Karahoda et al., 2022). The deletion strains could be complemented by the introduction of endogenous loci into the deletion strains (Fig. S2 and S3).

**Fig. 2.**
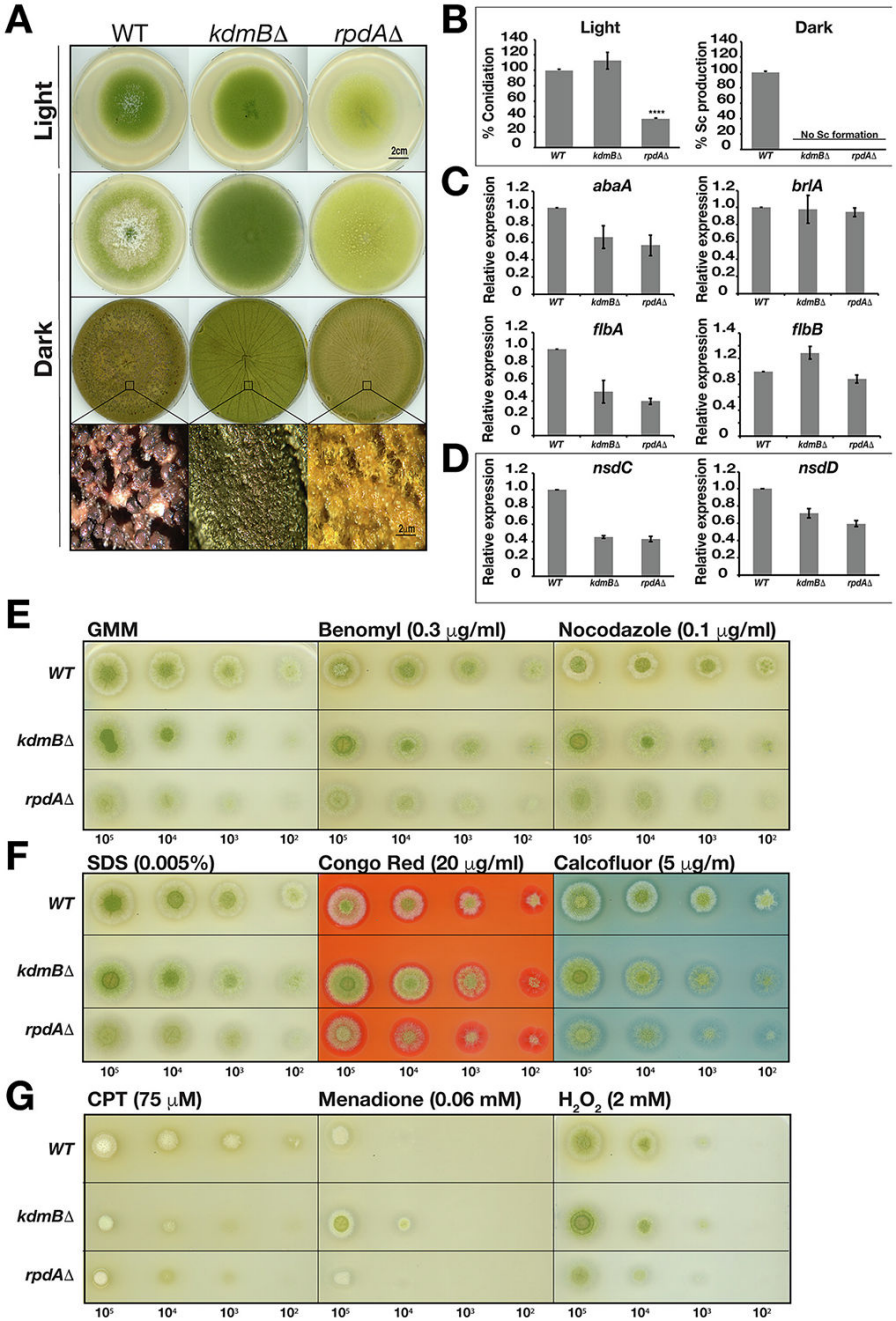
KdmB and RpdA are essential for sclerotia development. (A) For conidiation analysis, ~5x10^3^ spores of WT, *kdmB* and *rpdA* mutants were spot inoculated onto PDA plates and grown for 4 days at 30 °C under illumination (upper lane). For sclerotia induction, PDA (5 days) and WKM plates (21 days) were incubated in dark conditions at 30 °C. Lower section shows the stereomicroscopic images of sclerotia formation in WT which was totally abolished in *kdmB* and *rpdA* mutants. (B) Percentage of conidiation and sclerotia production in WT and deletion strains. Final values were normalised with respect to WT representing 100% production. (C) RT-qPCR expression analysis of conidia regulatory genes *abaA*, *brlA*, *flbA*, *flbB* and (D) sclerotia regulatory genes *nsdC* and *nsdD*. Fungal mycelia were shifted onto agar plates from submerged cultures grown for 24 h at 30 °C. For the analysis of conidia regulatory genes, total mRNA was obtained by harvesting fungal mat grown on PDA plates for 3 days at 30 °C under illumination. For the analysis of the mRNA expression profiles of sclerotia regulatory genes, WKM agar plates were cultured for 5 days at 30 °C in dark conditions. WT mRNA levels were adjusted to 1.0. RT-qPCR experiments were carried out in two independent biological replicates and six technical replicates. (E, F, G) The effects of *kdmB* and *rpdA* on various stress agents. Glucose minimal media (GMM) agar plates were supplemented with various stress inducing agents (oxidative stress, menadione and H2O2, cell wall stress, SDS, congo red and calcofluor, cytoskeleton stress, Nocodazole and Benomyl, DNA damage, Camptothecin (CPT)) at the concentrations indicated and were incubated for 3 days under light conditions. All phenotypic tests were carried out in three independent biological replicates.

Phenotypic tests on potato dextrose agar (PDA) media showed that light induced asexual conidiation was negatively affected (e.g. reduced by ~ 70%) in the *rpdA* mutant when compared to the WT strain (Fig. 2A). On the contrary, asexual conidiation appears slightly higher in the *kdmB*Δ mutant, but the increase is not statistically significant (Fig. 2B). Similarly, a slight (statistically non-significant) increase in conidiation was also observed when the mutant was grown in the dark (Fig. 2A). Expression analysis of key transcription factors required for *Aspergillus* conidiation showed that *abaA*, *flbA* and *flbB* were downregulated in *rpdA*Δ. *flbA* and *abaA* expression were also reduced in the *kdmB*Δ mutant, whereas *flbB* was found to be upregulated, possibly explaining why there was a slight induction of conidiation in *kdmB*Δ (Fig. 2C). These data suggest that the KERS complex is required for the expression of asexual conidiation pathway in *A. flavus*.

### 2.3. KdmB and RpdA are essential for sclerotia development and stress resistance

The effect of the mutants on sclerotia development was studied. At the end of 21 days, no sclerotia formation was present in either the *kdmB*Δ or *rpdA*Δ mutants (Fig. 2A, 2B), similar to what was reported for SntB loss (Pfannenstiel et al., 2018). *nsdC* and *nsdD*, which encode proteins required for sclerotia production, were found to be downregulated in the *kdmB*Δ and *rpdA*Δ mutants, indicating a positive role of the KERS complex in sclerotia production (Fig. 2D).

Having found that the KERS complex is important for development in *A. flavus*, we wondered if this complex was also involved in responses to environmental stresses. Therefore, both mutants were subjected to various stress tests, including microtubule, DNA damage, high osmolarity, oxidative and cell wall stress (Fig. 2E). It was observed that both *kdmB*Δ and *rpdA*Δ mutants were more susceptible to the topoisomerase inhibitor camptothecin (CPT), suggesting their possible role in DNA replication during the cell cycle (Fig. 2G). Interestingly, although the *rpdA*Δ strain did not exhibit any difference in growth on menadione-mediated oxidative stress when compared to WT, the *kdmB*Δ strain was found to be more tolerant (Fig. 2G). Both mutants and WT were similarly sensitive to hydrogen peroxide. This is in agreement with the role of similar Snt2-Ecm5-Rpd3 complex in yeast, where this complex regulate genes in response to oxidative stress (Baker et al., 2013). However, we could not detect any effect of the cellular stressors benomyl, nocodazole and cell-wall stressors, SDS, congo red and calcofluor on either the *kdmB*Δ or *rpdA*Δ mutants when compared to WT (Fig. 2E, 2F). Together, these results suggest that not only is the KERS complex essential for development, but it is also involved in the response to different stress factors.

### 2.4. KdmB and RpdA are essential for aflatoxin production in solid media and on peanut seeds

**Fig. 3.**
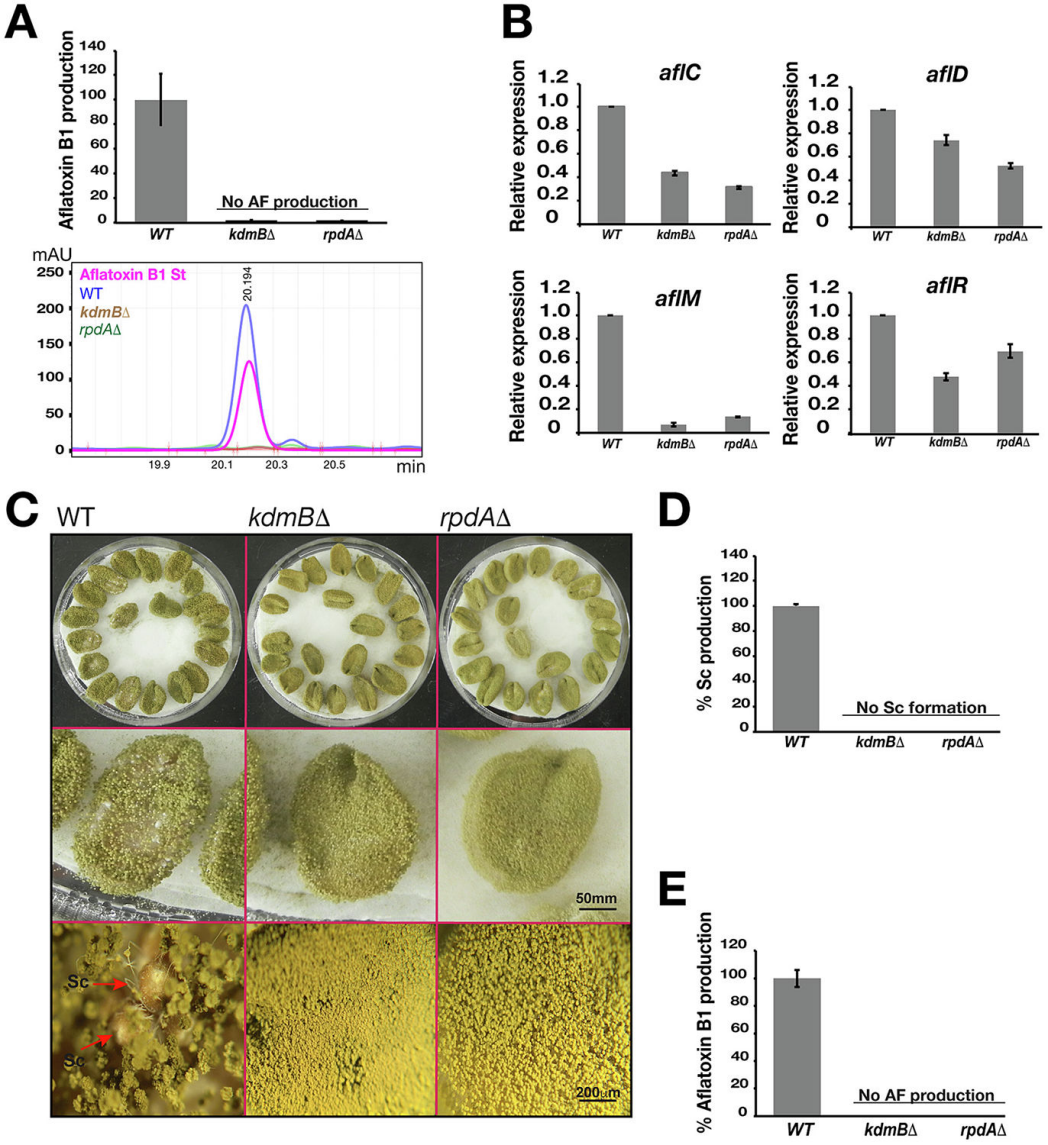
KdmB and RpdA are essential for aflatoxin production and play vital roles in peanut seed contamination. (A) Aflatoxin B_1_ analysis by reversed-phase HPLC in WT, *kdmB* and *rpdA* mutant strains grown on YES agar media. WT represents 100 % production. Lower panel shows the chromatogram of aflatoxin B1 peaks obtained from standard (Sigma), WT and *kdmB*, *rpdA* mutants. (B) Relative expression analysis of aflatoxin regulatory gene clusters *aflC*, *aflD*, *aflM* and *aflR*. Total mRNA was obtained by harvesting fungal mat grown on PDA plates for 3 days at 30 °C under dark conditions. (C) Peanut infection assay of WT and mutant strains. Peanut seeds were infected with ~ 5x10^3^ spores of WT, *kdmB*Δ and *rpdA*Δ strains and incubated for 5 days at 30 °C in a dark environment. Mock control peanut seeds without any fungal treatment are not included in the figure. (D) The number of sclerotia produced on seeds infected with WT and mutant strains. Sclerotia number grown on each peanut were manually counted and adjusted relative to WT set at 100. (E) RP-HPLC analysis of aflatoxin from infected peanut seeds. All values are the average of three independent biological replicates and error bars represent standard errors.

Development is often linked with SM production in filamentous fungi. To this end, having discovered that KERS is essential for sclerotia development, we wondered if KdmB and RpdA are also required for AF production in *A. flavus*. Therefore, total crude organic extracts from mutants and WT grown on yeast extract sucrose (YES) agar plates were isolated by chloroform extraction. Reversed-phase HPLC analysis revealed that AF production could not be detected in *kdmB*Δ or *rpdA*Δ (Fig. 3A), suggesting that KdmB and RpdA are important for AF production. We next determined expression of the AF biosynthetic (*aflC*, *aflD*, *aflM*) and regulatory genes (*aflR*) by RT-qPCR in the *kdmB*Δ, *rpdA*Δ and WT strains. All four genes were moderately downregulated in both mutants when compared to WT, with the most drastic reduction seen in *aflM* expression (Fig. 3B). It is interesting to note that AF production was totally lost even though the four AF biosynthesis genes analyzed were still expressed albeit at lower levels.

Next we performed infection tests using raw peanut seeds to monitor the impact of the *kdmB*Δ and *rpdA*Δ mutants on growth on seeds. We did not see any effect on surface colonization, and there was no sign of sclerotia production observed in the deletion strains, while the WT produced premature sclerotia at the end of the 5th day of growth in dark conditions (Fig. 3C, 3D). More importantly, AF could not be detected from peanut samples infected with *kdmB*Δ or *rpdA*Δ mutants, while WT infected seed were contaminated with aflatoxin B_1_ (Fig. 3E). Together, these results underlined the critical role of KdmB and RpdA in sclerotia development and aflatoxin production.

## 3. KdmB and RpdA are global regulators of secondary metabolite production

**Fig. 4.**
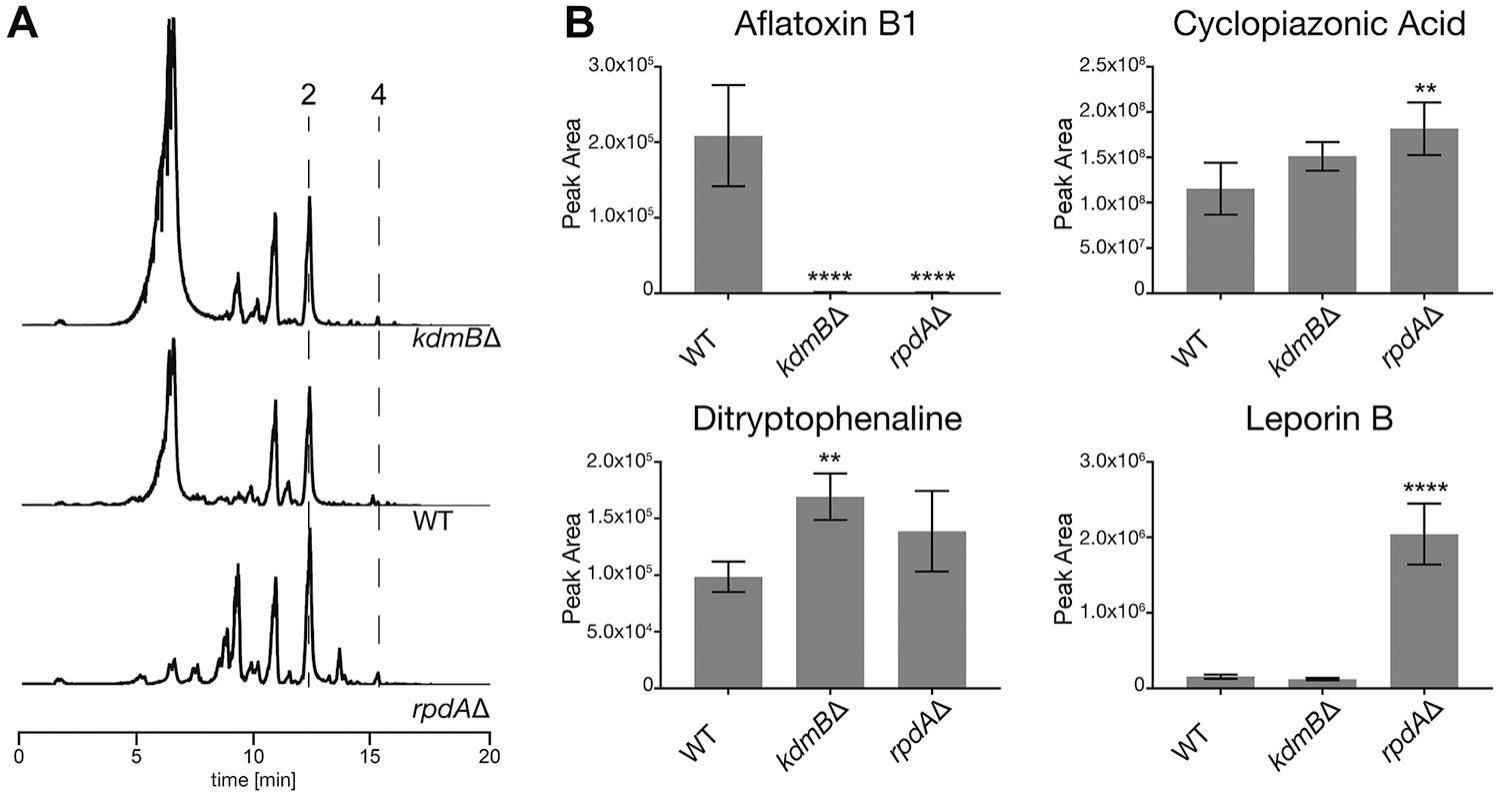
Secondary metabolite analysis of deletion strains via LC-MS. (A) Base peak chromatograms generated from ES^−^ mode of WT and *kdmB*Δ and *rpdA*Δ mutant strains. Peaks correspond to; 2 = CPA (Cyclopiazonic Acid), 4 = Leporin B. (B) Individual graphs of known secondary metabolites produced by *A. flavus*. Average peak area and standard deviation were calculated from four biological replicates. Statistical significance was calculated using one way ANOVA. *P*-value **p < 0.01, ****p < 0.0001.

The KERS complex subunit SntB has recently been shown to play a crucial role in SM production in *A. nidulans* and *A. flavus* (Pfannenstiel et al., 2018; Pfannenstiel et al., 2017). To determine whether KdmB and RpdA controls production of major metabolites (i.e. beyond AF), we performed ultra-high-performance high resolution mass spectrometry (UHPLC-HRMS) to determine the levels of other known metabolites such as aflavarin (Cary et al., 2015a), aflatrem (Nicholson et al., 2009), asparasone A (Cary et al., 2014), AF B1 (Yu et al., 2004), cyclopiazonic acid (Chang et al., 2009), ditryptophenaline (Saruwatari et al., 2014) and leporin B (Cary et al., 2015b) (Fig. 4A). Visualization using metabolomics data analyser MAVEN identified known metabolites and further confirmed that AF B1 production was completely abolished in the *kdmB*Δ and *rpdA*Δ strains. On the other hand, the deletion strains produced increased levels of cyclopiazonic acid and ditryptophenaline when compared to WT (Fig. 4B). Leporin B production was shown to be reduced in *kdmB*Δ while *rpdA*Δ produced significantly higher levels of this metabolite. Aflavarin, aflatrem, or asparasone A levels did not show any statistical difference between WT and the mutant strains (data not presented). These results suggest that KdmB and RpdA are negative-regulators of cyclopiazonic acid and ditryptophenaline production, whereas KdmB and RpdA are positive and negative regulators of Leporin B synthesis, respectively (Fig. 4B). These results corroborate the role of KERS complex as global regulator of mycotoxins, consistent with the findings for the *A. nidulans* and *Penicillium expansum* KERS complexes (Karahoda et al., 2022; Luciano-Rosario et al., 2023).

### 3.1. KdmB and RpdA regulate the expression of predicted SM backbone genes

**Fig. 5.**
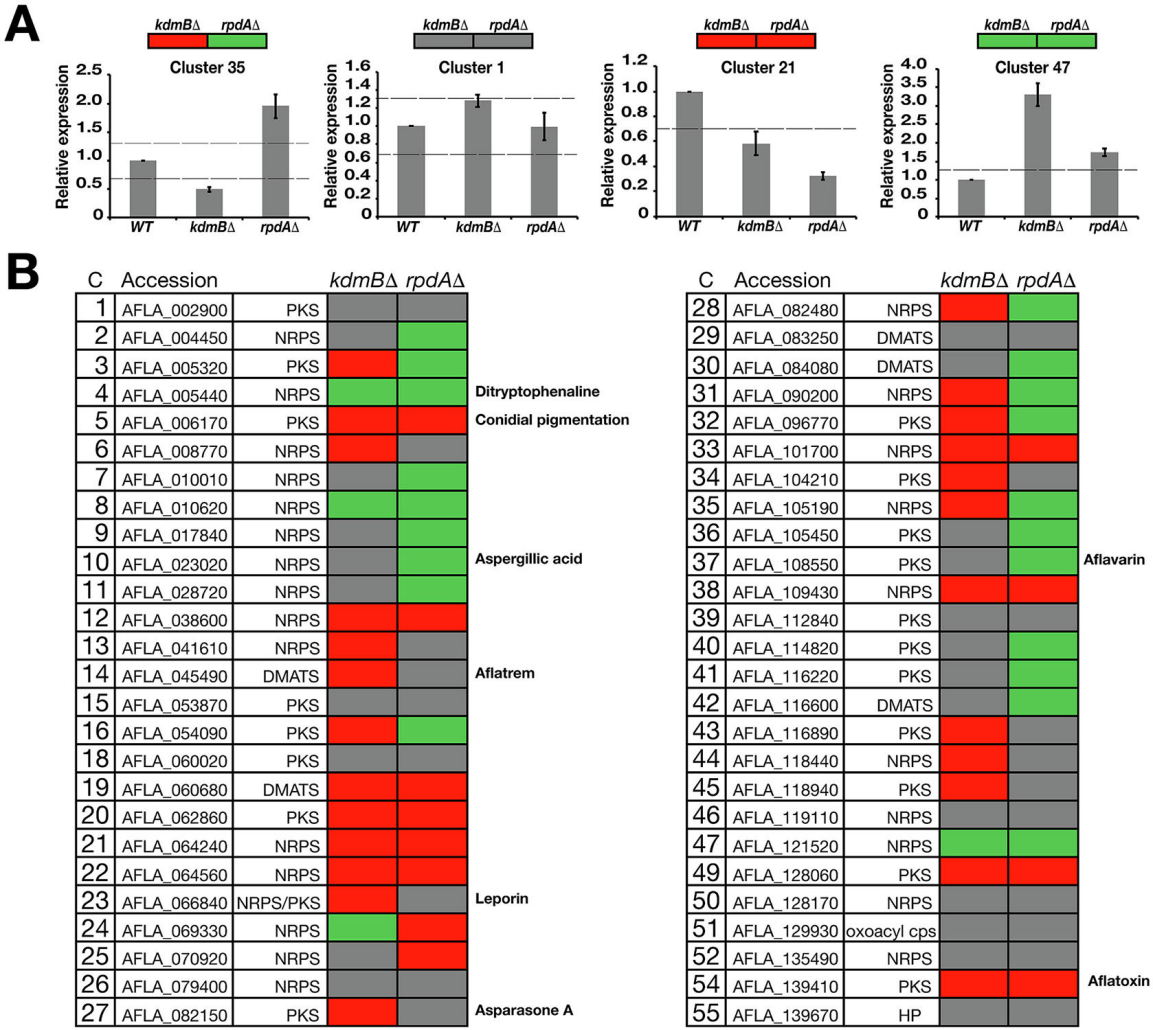
KdmB and RpdA are global regulators of secondary metabolite production in *A. flavus*. (A) Four representatives of RT-qPCR expression analysis showing either upregulation, downregulation or no change compared to WT. Grey box represents no change, red box represents down-regulation by 30 % or more and green box represents 30% upregulation or more. cDNA was obtained from total mRNA extracted from fungal samples grown on PDA plates for 72 h at 30 °C in dark conditions. (B) mRNA profiles of 52 secondary metabolite cluster backbone genes corresponding to predicted NRPS, PKS, dimethylallyl tryptophan synthase, 3-oxoacyl carrier protein synthase and a hypothetical protein (HP). All values are the average of two independent biological replicates and 6 technical replicates. The error bars represent standard errors. See Supplementary S3 Table for full description of secondary metabolite gene clusters.

We were interested in the role of KdmB and RpdA on global SM production in *A. flavus* SM as the *sntB* deletion resulted in changes in the production of many SMs in this species in addition to AF (Pfannenstiel et al., 2018). Genome analysis predicted that the *A. flavus* genome contains 56 backbone genes (encoding putative synthetases/synthases known to produce SMs) (Ehrlich and Mack, 2014; Fountain et al., 2020; Skerker et al., 2021). We therefore assessed the relative mRNA levels of the 55 backbone genes (identified by the SMURF program) in *kdmB*Δ, *rpdA*Δ and WT strains (Table S3). The expression of 3 out of 55 genes was not detected in our selected growth condition, while 52 genes were expressed (Fig. 5). Considering the above observation that a slight reduction (e.g. ~ 20–30%) in the AF biosynthesis genes could lead to complete loss of AF production (Fig. 3A and B), we used a slightly less stringent cut-off than the standard (e.g. two-fold) to identify possible effects of the mutants on their overall secondary metabolism; e.g. a minimum of 30% change in expression of backbone genes was considered as up (Green box) or down-regulated (Red box) (Fig. 5A). Remarkably, 41 of the backbone genes (corresponding to ~ 79% of all SM backbone genes) were affected in either *kdmB*Δ or *rpdA*Δ strain. For genes that are commonly affected in both mutants, 10 genes were downregulated but only 3 genes (all NRPS) were up-regulated, emphasizing that the KERS complex generally acts as a positive-regulator of SM backbone genes. When analyzing the mutants individually, expression of 25 SM backbone genes (11 NRPS, 11 PKS, 2 DMATS and 1 NRPS/PKS hybrid) was negatively affected while only 4 genes (all NRPS) were positively affected in *kdmB*Δ, demonstrating an overall activating role for KdmB in regulating gene transcription. In contrast to *kdmB*Δ, in the *rpdA*Δ mutant, expression of 20 genes were affected positively (11 NRPS, 7 PKS and 2 DMATS), while expression of 12 genes were affected negatively (7 NRPS, 4 PKS and 1 DMATS), suggesting RpdA acts predominantly as a repressor of certain secondary metabolite backbone gene transcription.

The conidial pigmentation genes (Cluster 5) (Wang et al., 2016) were down-regulated in both mutants. Interestingly, the asparasone A (Cluster 27) (Chang et al., 2017)) and *lepA* genes (Cluster 23) (Cary et al., 2015b)) were down-regulated in the *kdmB*Δ mutant while there was no significant change of these genes in the *rpdA*Δ mutant when compared to WT. Similarly, the aflatrem gene (Cluster 14) (Chang et al., 2017) was down-regulated in the *kdmB* mutant, while it did not change in the *rpdA* mutant with respect to WT, emphasizing a distinct role of KdmB in the regulation of asparasone A and aflatrem. On the other hand, expression of the NRPS gene for biosynthesis of aspergillic acid (Cluster 10) (Lebar et al., 2018)) and the PKS gene synthesizing an insecticide metabolite aflavarin (Cluster 37) (Cary et al., 2015a)) did not change in *kdmB*Δ while they were increased in the *rpdA*Δ strain, indicating repressive roles of RpdA in the production of these metabolites. Previously, SntB was shown to be a positive regulator of aflavarin, AF B_1_, asparasone A, aflatrem and a negative regulator of ditryptophenaline and leporin B (Pfannenstiel et al., 2018). Our results show that KdmB acts as a positive-regulator of asparasone A, aflatrem, AF and leporin backbone gene mRNA levels highlighting the common KERS complex functions on regulation of SMs. RpdA, on the other hand, does not have any effect on expression of aflavarin or asparasone backbone genes. RpdA is a positive regulator of AF and a negative regulator of aspergillic acid and aflavarin. Therefore, KERS complex components KdmB, RpdA and SntB are positive-regulators of AF production while RpdA and SntB may have opposing roles in aflavarin production.

### 3.2. KERS complex regulates in vivo histone modifications

**Fig. 6.**
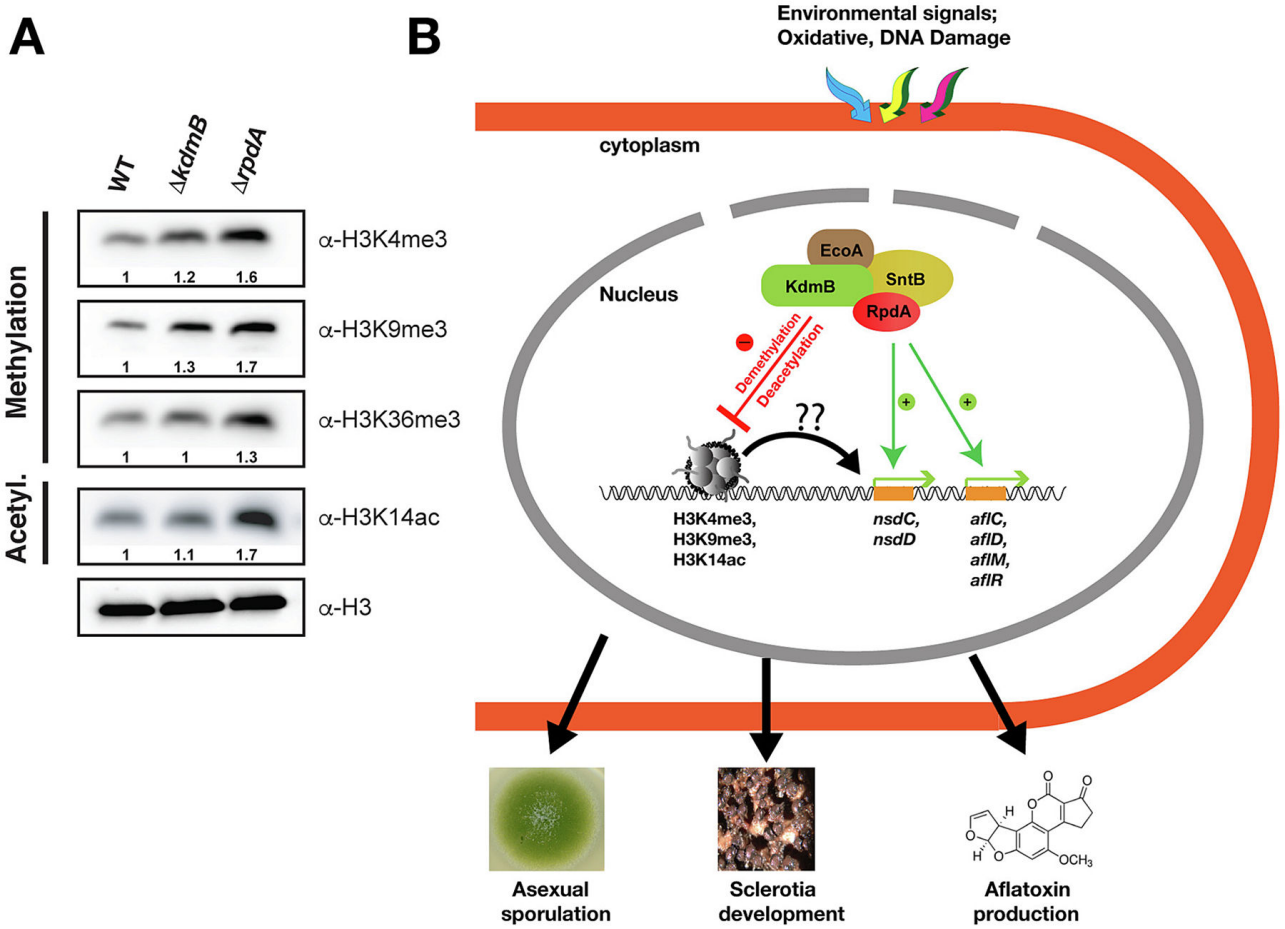
KERS complex has *in vivo* HDM and HDAC activities. (A) Histone PTM analysis using various antibodies against methylated and acetylated histone lysine residues. For nuclear enrichment, approximately 2x10^6^ spores were inoculated into GMM with required supplements and grown for 24 h at 30 °C submerged culture. Quantification of band intensities was performed from two independent biological replicates using imageJ software. H3 was used as loading control and as a reference signal intensity for quantifications (B) Schematic model representing the role of the KERS complex on the fungal development and SM production. The KERS complex negatively affects global H3K4me3, H3K9me3, H3K36me3 methylation levels. RpdA remarkably represses H3K14 acetylation. KdmB and RpdA are positive regulators of sclerotia development and aflatoxin biosynthesis through regulation of *nsdC*, *nsdD* and AF gene cluster pathways respectively.

In *A. nidulans*, it was shown that KdmB targets H3K4me3 residues *in vivo* and K3H9me3 and H3K4me3 residues *in vitro* (Gacek-Matthews et al., 2016). RpdA, on the other hand, was shown to have global HDAC activity for acetylated H3 and H4 histone tails (Tribus et al., 2010). A recent study in *Magnaporthe oryzae* suggested that MoSnt2 interacts with histone deacetylase Hos2 (*A. nidulans* HosA) to mediate H3 deacetylation and plant infection (He et al., 2018). In *A. flavus*, SntB was shown to negatively regulate H3K9 and H3K14 acetylation (Pfannenstiel et al., 2018). In order to analyse histone PTMs regulated by KdmB and RpdA in *A. flavus*, we carried out a nuclei extraction protocol to enrich for histones in *kdmB*Δ, *rpdA*Δ and WT strains. Interestingly, H3K4me3 and H3K9me3 levels were up-regulated in the *kdmB*Δ strain comparing to wild-type. On the other hand, H3K36me3 levels did not change in *kdmB*Δ suggesting that this residue is not targeted by KdmB. Notably, H3K36me3 levels were increased by 30% in the *rpdA* mutant when compared to wild-type. Previous studies suggested that RpdA acts on global histone modifications including H3K9ac that controls ST production in *A. nidulans* (Reyes-Dominguez et al., 2010; Tribus et al., 2010). Surprisingly, we did not see any change in H3K9ac levels in the *rpdA*Δ mutant (data not presented). However, an increase in H3K14ac residues occurred in the absence of *rpdA*, suggesting that the HDAC RpdA mainly targets H3K14ac residues. As expected for a demethylase, KdmB deletion did not have any effect on histone acetylation levels (Fig. 6A). Therefore, KdmB and RpdA regulate H3K4me3, H3K9me3, H3K36me3 and H3K9ac levels. These results indicate that these proteins influence global histone PTMs and the situation at a specific locus might be different. We propose that these chromatin functions at specific loci contribute to the overall regulation of development, SM production and pathogenicity (Fig. 6B).

## 4. Discussion

Chromatin modifying complexes play important roles in cell proliferation, cell survival and numerous other cellular pathways by regulating histone PTMs. It is expected that proper transcriptional regulation of fungal development, mycotoxin production and pathogenicity depends on histone PTMs. Previously, we discovered the chromatin binding KERS complex in *A. nidulans*. In this paper, we report the conservation of the KERS complex formation in the pathogenic fungus *A. flavus* (Fig. 1C). It is interesting to note the presence of a partial complex (KRS) composed of the KdmB, RpdA and SntB subunits (i.e. lacking EcoA) in species of the *Penicillium* genus (Luciano-Rosario et al., 2023). The functional significance of the difference in the complex composition is not clear, but it highlights the importance of having coordinated recruitment of KdmB, RpdA and SntB to chromatin.

We found that the *ecoA*Δ mutation was inviable, while the *kdmB* and *rpdA* genes can be deleted. Unlike in yeast, *rpdA* was shown to be essential in *A. fumigatus* and *A. nidulans* (Karahoda et al., 2022; Rundlett et al., 1996; Tribus et al., 2010). Successful generation of *rpdA*Δ in *A. flavus* suggests that RpdA in this fungi may be functionally more closely related to yeast Rpd3 and/or RpdA has redundant functions with another histone deacetylase.

KdmB and RpdA are both eraser proteins which influence global histone PTMs affecting development, SM production and pathogenicity. This hypothesis is supported by the fact that both *kdmB* and *rpdA* deletions resulted in the lack of sclerotia formation. The KERS complex members are found in both budding and fission yeast. In budding yeast, the SntB homolog,Snt2 forms a complex with RpdA (Rpd3) as well as a JmjC protein Ecm5, but not with the EcoA (Eco1) or KdmB (Jhd2) homologs (Baker et al., 2013). *S. cerevisiae* has a total of five JmjC domain-containing proteins namely Jhd1, Jhd2, Rph1, Ecm5 and Gis1 (Baker et al., 2013). Furthermore, Ecm5 and Snt2 were shown to be responsible for the recruitment of Rpd3 to a number of promoter regions. In the fission yeast, *S. pombe*, the SntB homolog (Snt2), KdmB homolog (Lid2), and three other proteins, Ash2, Sdc1 and Jmj3 form a five member complex (Roguev et al., 2003). In *A. nidulans*, the KERS complex is recruited to the core promoter of more than 1,600 genes (Karahoda et al., 2022) playing pleotropic roles in diverse physiological pathways and cellular processes.

There is some evidence that certain members of the KERS complex may interact in animals. Lid2 interacts with FOXO and promotes the activation of oxidative-response genes under oxidative stress by recruiting HDAC4 to FOXO in order to facilitate FOXO deacetylation which affects FOXO DNA binding (Liu et al., 2014). KDM5C has been shown to interact with two distinct histone deacetylase complexes, the SIN3B-HDAC and the HDAC NuRD complexes (Gajan et al., 2016; Nishibuchi et al., 2014). The human ortholog EcoA, a homolog of Eco1, was found to be associated with Lysine-specific-demethylase 1 (LSD1) (Choi et al., 2010).

It remains unknown if EcoA directly contributes to histone modification in filamentous fungi, since we could not obtain a viable *ecoA* deletion strain. A conditional knock-down mutant would help to investigate its genome wide effect in future studies. Ortholog analysis strongly suggest that KERS members are highly conserved across various fungi and human. In addition to KERS complex in *A. nidulans*, this and an associated paper (Luciano-Rosario et al., 2023) together suggest that KERS complex may be functionally and biochemically conserved in other pathogenic fungi and may be conserved in higher eukaryotes and human cells.

We showed that KdmB and RpdA functions are essential for sclerotia development (Fig. 2.A, 2C, 2D). Gene expression analysis suggested that RpdA regulates conidiation by targeting and positively activating the expression of *abaA* and the upstream conidia regulator *flbA* (Fig. 2C). As opposed to *rpdA*Δ, conidiation is increased in the *kdmB* mutant indicating opposite functions of KdmB and RpdA for light-induced sporulation. The *kdmB*Δ and *rpdA*Δ mutants show sensitivity against the topoisomerase inhibitor camptothecin (CPT), while the *kdmB*Δ mutant is more resistant towards menadione-mediated oxidative stress (Fig. 2G), emphasizing their critical roles against stress response agents. In yeast, *ecm*5Δ was shown to be sensitive towards oxidative stress (Baker et al., 2013). As opposed to yeast *ecm*5Δ, *kdmB* deletion resulted in increased resistance toward menadione-mediated oxidative stress but did not have any effect against hydrogen peroxide, suggesting functional differences of KdmB and Ecm5 (Fig. 2G). Furthermore, EcoA homologs are responsible for cohesin acetylation, which is required for genome integrity and sister chromatid separation. Therefore, the sensitivity of *kdmB* and *rpdA* against topoisomerase inhibitor might stem from EcoA functions on cohesins and genome integrity.

The loss of AF production in the *kdmB*Δ and *rpdA*Δ mutants is consistent with the previous study carried out using a *sntB*Δ mutant which was shown to be unable to produce AF in *A. flavus* and ST in *A. nidulans* (Pfannenstiel et al., 2017), suggesting full assembly of KERS subunits are equally essential for transcriptional activation of AF production. It is interesting that AF production is totally lost while there is still moderate expression of AF gene cluster genes. A possible explanation for this is that expression of at least one AF biosynthesis genes not analysed in our experiment was completely abolished in the mutant. It is also possible the mutants might have affected the expression of another non-AF cluster gene (or genes) or lack the precursor metabolite(s) essential for AF production. Alternatively, the result may also suggest the existence of an additional unknown posttranscriptional regulation of AF biosynthesis genes for AF production. In addition to reduced expression of AF gene cluster, the production of several metabolites as well as transcription levels of nearly 80% of SM backbone genes analysed were affected in the *kdmB* and *rpdA* deletion mutants, the KERS complex is therefore a global regulator of SM biosynthesis (Figs. 4, 5).

Hence, this work suggests that the chromatin modifier KERS complex likely regulates mycotoxin production by targeting histone marks of the AF biosynthetic gene cluster. Since the KERS complex is a putative reader of H3K4me3, H3K9me3, H3K36me3 and H3K9ac marks. We propose that it facilitates development, SM production and pathogenicity through targeting histone tails for modification (Fig. 6B). KdmB clearly has H3K4me3 and H3K9me3 demethylase activities. We could not see any effect on H3K36me3 levels in *kdmB*Δ, while the *rpdA*Δ mutant showed increased H3K36me3. However, overall these data indicate that the KERS complex targets a number of histone modifications, which might be differentially regulated at a specific locus. Furthermore, KdmB and RpdA regulation of histone PTMs can be direct (like higher acetylation in *rpdA* mutants) or indirect (like H3K36me3 levels *in rpdA* mutants). It remains unknown if RpdA can associate with other chromatin readers such as methyltransferases, demethylases or heterochromatin protein. However, RpdA is able to form multiple complexes in *A. nidulans* such as RcLS2F co-KDAC repressor complex and it may potentially form similar complexes in *A. flavus* (Bauer et al., 2020). Further pulldowns with RpdA is required to show if RpdA is also a part of these complexes in *A. flavus*.

In *A. nidulans*, KERS binding sites on promoters do not correlate with target histone modifications (Karahoda et al., 2022). For example, KdmB ChIP-seq signals do not correlate with H3K4me3 ChIP-seq signals, suggesting that the KERS complex serves to localize the various chromatin modifiers to target promoters. It is currently unknown where the KERS complex binds in *A. flavus* genome and how these bindings can correlate with potential histone modifications and how these modifications can be translated into changes in expression of developmental regulators (such as *nsdC* and *nsdD*) and AF biosynthesis genes. In *A. nidulans*, the KERS complex also binds to the ST gene cluster and RNA Pol II occupancy is lower in this gene cluster in all KERS mutants. Since ST and AF gene clusters show structural and regulatory similarities, it will be interesting to see how the AF gene cluster is regulated by the KERS complex in *A. flavus*.

Lastly, the KERS complex might also be involved in transcription of histone genes since two subunits of the HIR complex required for repression of histone genes outside of S-phase (Prochasson et al., 2005) were consistently detected (Hir1 and Hir3) at higher peptide numbers in KERS purifications (Table S1-S2). However, this needs further studies to show a mechanistic interdependency between the KERS and HIR complexes.

Because the KERS complex exhibits a broad influence on development and SM production and is conserved in pathogenic fungi, it could be a good target not only for novel drug discovery but also to understand the epigenetic mechanisms contributing to fungal pathogenicity. Our findings in this paper provide an insight into how chromatin modifier protein complexes can have broad effects on growth, development and natural product biosynthesis by regulating histone PTMs. These epigenetic effects mediated by the KERS complex are probably conserved in filamentous fungi including plant and human pathogens.

## 5. Methods

### 5.1. Strains, growth media and culturing conditions

*Aspergillus flavus* strains used in this study are listed in S3 Table. For spore cultivation, strains were grown in GMM glucose minimal media at 30 °C in 1% glucose as the carbon source and nitrate as the nitrogen source as described earlier (Elramli et al., 2019). Uridine and uracil were supplemented when required. For phenotypic analysis, 5 μl (5x10^3^ spores) of spore suspension was spot inoculated onto petri dish. Asexual conidiation was observed at the end of 5 days grown in Potato Dextrose Agar (PDA) with supplements in light conditions at 30 °C. 0.5 cm diameter of agar surface was removed and resuspended in 500 μl PBS solution prior to counting spores on haemocytometers. For sclerotia analysis, spore suspensions were spot inoculated onto Wickerham medium (Chang et al., 2012) for 21 days in dark conditions at 30 °C. Sclerotia quantification was performed by manually counting the surface of each petri dish after washing the plate surface with EtOH. For aflatoxin analysis by RP-HPLC, 5 μl (5x10^3^ spores) were spot inoculated onto YES agar medium (20 g yeast extract, 150 g sucrose, 1 g MgSO_4_7H_2_O, 20 g agar) for 7 days in the absence of light at 30 °C. Stress-inducing agents for growth tests of wild-type and deletion strains where supplemented into GMM when necessary. Stereomicroscopic images were captured using the Olympus szx16 microscope with Olympus sc30 camera. Digital images were taken and processed with the Cell Sens Standard software (Olympus). Quantifications of asexual conidiation and sclerotia production were performed using three independent biological replicates. For protein extraction, spores were inoculated into complete media (GMM with 0.1% yeast extract, 0.2% peptone, 0.1% tryptone and required supplements) and grown for 24 h 200 rpm at 30 °C.

### 5.2. Generation of A. flavus deletion, complementation and epitope tagged strains

All oligonucleotides used in this study are listed in S4 Table. Plasmids employed in this study are listed in S5 Table. In order to create deletion constructs of *kdmB* and *rpdA* genes, the *A. fumigatus pyrG* marker was amplified from plasmid pME3858 using OZG694 and OZG695 primers yielding a 1.89 kbp fragment. To create pBK22, 5′ UTR (BK67/BK69) and 3′ UTR (BK70/BK71) regions were amplified by using the wild-type genomic DNA of NRRL3357 strain. These fragments were fused to *Sma*I-digested pUC19 by using In-Fusion HD Cloning kit, yielding pBK22 from which *kdmBΔ::AfpyrG* cassette was amplified (BK68/72) and transformed into recipient fungal strain. Similarly, to create pBK23, 5′ UTR (BK73/BK75) and 3′ UTR (BK76/BK77) regions were amplified by using the wild-type genomic DNA of NRRL3357 strain. These fragments were fused to *Sma*I-digested pUC19 by using In-Fusion HD Cloning kit, yielding pBK23 from which *rpdAΔ::AfpyrG* cassette was amplified (BK74/78) and transformed into recipient fungal strain. TJES19.1 (*pyrG^−^*) was used as recipient for DNA transformation into *A. flavus* that was cultured and transformed as previously described (de Assis et al., 2021; Punt and van den Hondel, 1992).

In order to create HA and GFP C-terminal epitope tags of KdmB, *3xha::AfpyrG* and *sgfp::AfpyrG* fragments were amplified using OZG916/694 from plasmids pOB430 and pOB435 respectively. To create pBK80, ORF (BK350/BK351) and 3′ UTR (BK352/BK353) regions were amplified by using the WT genomic DNA of NRRL3357 strain. These two fragments with *3xha::pyrG* were fused to *Sma*I-digested pUC19 by using In-Fusion HD Cloning kit, yielding pBK80 from which *kdmB::3xha::AfpyrG* cassette was amplified (BK354/355) and transformed into recipient fungal strain resulting in AFLBK1 (KdmB::3xHA. To create pBK79, ORF (BK350/BK351) and 3′ UTR (BK352/BK353) regions with *sgfp::AfpyrG* were fused to *Sma*I-digested pUC19, yielding pBK79 from which *kdmB::sgfp::AfpyrG* cassette was amplified (BK354/355) and transformed into recipient fungal strain TJES19.1, resulting in AFLBK2 (KdmB::sGFP).

### 5.3. GFP&HA-TRAP and sample preparation for LC-MS protein identification

Isolation and preparation of GFP and HA fusion proteins for mass spectrometry analysis was performed as explained in detail (Helmstaedt et al., 2008; Sarikaya Bayram et al., 2022; Sarikaya Bayram et al., 2019). GFP and HA experiments were performed from two independent biological replicates. WT strain protein extracts were treated with HA and GFP magnetic beads and eluates of the WT from these treatments were used to identify unspecific proteins that stick to the beads. Proteins identified from KdmB-HA and KdmB-GFP experiments were filtered from non-specific HA and GFP control purifications with WT to remove non-specific protein contaminants. The final lists of the proteins were presented in excel format in Supplementary tables (S1-S2).

### 5.4. Southern blot hybridization

Southern blot hybridization experiments were carried out using Roche DIG Nucleic Acid Detection Kit (Roche, 11175041910). Amplification of 5′UTR region (each yielding approximately 1.2 kbp) probes were carried out using non-radioactive digoxigenic (DIG) labelling kit (Roche, 11093657910).

### 5.5. Confocal microscopy

Microscopy experiments were performed as described previously (Bayram et al., 2019). Strains expressing sGFP were grown in Lab-Tek chambered Coverglass W/CVT (Thermo Scientific, 155360) in 400 μl GMM with required supplements for 16 h at 25 °C. DRAQ5 (Sigma) with 1:10,000 dilution was used for nuclear staining 30 min prior to imaging under microscope. Microscopic images were captured using the Olympus FV1000 confocal microscopy in 60x magnification.

### 5.6. Immunoblotting

For GFP-tagged KdmB, mouse α-GFP antibody (Cat# SC-9996, SantaCruz) was used at 1:1,000 dilution in blocking solution (TBST with 5% milk). Secondary goat α-mouse (Cat# 170–6516, Biorad) was used at 1:2,000 dilution in blocking solution. A rabbit polyclonal antibody against a subunit of the SCF complex, SkpA (SconC) was used (1:2,000 dilution in TBST with 5% milk) as a loading control with the Goat α-Rabbit (Bio-Rad, Cat# ab190479; 1:2,000) secondary antibody. For nuclear isolation, approximately 2x10^6^ spores were inoculated into GMM with required supplements for 24 h at 30 °C submerged culture. Buffers and extraction protocol were performed similarly as described previously (Soukup and Keller, 2013). Antibodies (1:2,000 dilution in blocking solution) used for histone PTMs were as following; α-H3 (Abcam; AB1791), α-H3K4me3 (Active Motif; 39159), α-H3K9me3 (Abcam; ab8898), α-H3K36me3 (Abcam; ab9050), α-H3K9ac (Merck; 07–352), α-H3K14ac (Merck; 07–353).

### 5.7. RNA extraction and quantitative real time PCR analysis

100 mg of mycelia was collected and mRNA was isolated according to the ‘RNeasy Plant Mini Kit’ manufacturer’s protocol (Qiagen). mRNA was quantified using ‘Qubit RNA BR Assay Kit’ Protocol (Thermo Fisher). cDNA was synthesised from 1 μg of mRNA using the ‘Transcriptor First Strand cDNA Synthesis Kit’ (Roche). qPCR reaction mixtures were prepared using LightCycler 480 SYBR Green I Master mix and a LightCycler 480 qPCR (Roche) was used to determine gene expression levels. Beta-tubulin (*benA*) control was used as a housekeeping gene. Bar charts represent the mean data of two combined biological replicates and 6 combined technical replicates per strain.

### 5.8. Pathogenicity tests

Pathogenicity assays were performed similar to previously described (Christensen et al., 2012). Skins of raw peanut seeds were peeled off, washed with ethanol for 5 min and rinsed with sterile water for 5 min. Rinsing step was repeated three more times and then seeds were let air dry for 30 min under sterile cabinet. Ten peanut seeds were placed into sterile 50 mL centrifuge tubes and incubated with 5x10^3^ per ml conidia in PBS for 30 min on shaker at room temperature. Non-infected peanut seeds were used as mock. Ten infected peanut seeds for each strain and mock were placed onto sterile cellulose paper and covered with petri dish. Samples were incubated at 30 °C under dark condition for five days.

### 5.9. RP-HPLC analysis of aflatoxin levels

To analyse aflatoxin levels from culture media, approximately 2 cm of agar cores were removed from the centre of culture plates and were subjected to chloroform extraction to collect the organic phase. Samples were dried under speedy-vac and resuspended in methanol for HPLC analysis. To analyse aflatoxin from infected peanuts, one infected peanut seed for each strain was collected and incubated for 30 min with 6 mL chloroform:dH_2_O (v/v) at 4 °C. Samples were centrifuged at 4000 rpm for 15 min at 4 °C. Organic phase was transferred into microfuge tube. Chloroform was dried out under speedy-vac and samples were resuspended in methanol prior to HPLC analysis. 3 biological replicates were prepared for each experiment. Same procedure was repeated twice. WT was adjusted to 100%.

### 5.10. Secondary metabolite analysis

For secondary metabolite analysis, strains were point inoculated and grown at 30 °C on potato dextrose agar (PDA) in a 90 mm petri dish wrapped with Parafilm. After 12 days, a 1.0 cm core was taken from the plate and homogenized in 3 mL of 0.01% Tween 20 added, then extracted with 3 mL of ethyl acetate. Samples were shaken and spun for 10 min at 3000 rpm. The organic layer was removed, dried down, and resuspended in 100% acetonitrile, and.filtered through an Acrodisc syringe filter with a nylon membrane (Pall Corporation; 0.45 μm pore size). Ultra-high-performance high resolution mass spectrometry (UHPLC-HRMS) was then performed on a Thermo Scientific-Vanquish UHPLC system connected to a Thermo Scientific Q Exactive Orbitrap mass spectrometer in ES^+^ or ES^−^ mode between 200 *m/z* and 1000 *m/z* to identify metabolites. A Zorbax Eclipse XDB-C18 column (2.1 × 150 mm, 1.8 μm particle size) was used with a flow rate of 0.2 mL/min for all samples. LCMS grade water with 0.5% formic acid (solvent A) and LCMS grade acetonitrile with 0.5% formic acid (solvent B) were used with the following gradient 0 min, 20% Solvent B; 2 min, 20% Solvent B; 15 min, 95% Solvent B; 20 min, 95% Solvent B; 20 min, 20% Solvent B; 25 min, Solvent B. Nitrogen was used as the sheath gas. Data acquisition and UHPLC-MS were controlled by Thermo Scientific Xcalibur software. Files were converted to the.mzXML format using MassMatrix MS Data File Conversion, and analyzed in MAVEN and XCMS (24, 25).

### 5.11. Protein alignment and ortholog analysis

Data was obtained using Basic local alignment tool (BLAST) with *A. flavus* KERS protein sequences as reference. Listed orthologs correspond to the proteins of highest scores obtained from the most significant alignments while values represent % of identification of protein sequence (Fig. 1A).

## Supplementary Material

supplemental1

supplemental2

supplemental3

supplemental4

supplemental5

supplemental6

figure S1

Figure S2

Figure S3

## Data Availability

Data will be made available on request.
