# Supplementary material for: The KdmB-EcoA-RpdA-SntB (KERS) chromatin regulatory complex controls development, secondary metabolism and pathogenicity in Aspergillus flavus
Source: Fungal Genet Biol. Author manuscript; Available in PMC 2024 Feb 5. (PMC10841535; doi:10.1016/j.fgb.2023.103836)
Supplement: supplemental1 [file NIHMS1938650-supplement-supplemental1.pdf]

Table S1. KdmB::sGFP interacting protein list.

Accession	Description	Score	Coverage	# Proteins	# Unique Peptides	# Peptides	# PSMs	# AAs
CADAFLAP0001068	pep:known supercontig:JCVI-afl1-v2.0:EQ963482:1685104:1690608:-1 gene:CADAFLAG00010689 transcript:CADAFLAT00010689 description: PHD transcription factor, putative	799.99	63.62	1	90	90	170	1704
CADAFLAP0000237	pep:known supercontig:JCVI-afl1-v2.0:EQ963473:1948258:1953549:1 gene:CADAFLAG00002374 transcript:CADAFLAT00002374 description: PHD finger and BAH domain protein (Snt2), putative	322.06	50.90	1	62	62	81	1713
CADAFLAP0000397	pep:known supercontig:JCVI-afl1-v2.0:EQ963474:2204554:2210905:-1 gene:CADAFLAG00003973 transcript:CADAFLAT00003973 description: Transcriptional corepressor of histone genes (Hir3), putative	156.00	26.47	1	36	36	42	2059
CADAFLAP0000399	pep:known supercontig:JCVI-afl1-v2.0:EQ963474:2276434:2279944:-1 gene:CADAFLAG00003996 transcript:CADAFLAT00003996 description: Histone transcription regulator Hir1, putative	146.67	39.22	1	28	28	35	1058
CADAFLAP0000278	pep:known supercontig:JCVI-afl1-v2.0:EQ963473:3053401:3056239:-1 gene:CADAFLAG00002780 transcript:CADAFLAT00002780 description: Mitochondrial aconitate hydratase, putative	77.62	39.62	1	21	21	23	785
CADAFLAP0000301	pep:known supercontig:JCVI-afl1-v2.0:EQ963473:3679184:3681492:-1 gene:CADAFLAG00003012 transcript:CADAFLAT00003012 description: Phosphoenolpyruvate carboxykinase AcuF	120.61	51.67	1	20	20	28	600
CADAFLAP0000892	pep:known supercontig:JCVI-afl1-v2.0:EQ963480:874863:877102:1 gene:CADAFLAG00008921 transcript:CADAFLAT00008921 description: Histone deacetylase RpdA/Rpd3	85.71	43.07	1	20	20	23	685
CADAFLAP0000461	pep:known supercontig:JCVI-afl1-v2.0:EQ963475:1215660:1217220:-1 gene:CADAFLAG00004614 transcript:CADAFLAT00004614 description: Aldehyde dehydrogenase AldA, putative	98.83	55.33	1	19	19	25	497
CADAFLAP0000885	pep:known supercontig:JCVI-afl1-v2.0:EQ963480:687870:689811:1 gene:CADAFLAG00008853 transcript:CADAFLAT00008853 description: Putative uncharacterized protein Has domain(s) with predicted cell outer meml	83.76	57.36	1	16	16	22	401
CADAFLAP0001123	pep:known supercontig:JCVI-afl1-v2.0:EQ963483:1281340:1283202:-1 gene:CADAFLAG00011231 transcript:CADAFLAT00011231 description: Isocitrate lyase	72.12	45.54	1	16	16	18	538
CADAFLAP0000596	pep:known supercontig:JCVI-afl1-v2.0:EQ963476:2187122:2194103:1 gene:CADAFLAG00005963 transcript:CADAFLAT00005963 description: Acetyl-CoA carboxylase, putative	49.75	10.07	1	16	16	16	2125
CADAFLAP0000090	pep:known supercontig:JCVI-afl1-v2.0:EQ963472:2412375:2414764:1 gene:CADAFLAG00000902 transcript:CADAFLAT00000902 description: ARS binding protein Abp2, putative	80.14	31.84	1	15	15	20	779
CADAFLAP0001093	pep:known supercontig:JCVI-afl1-v2.0:EQ963483:417154:418937:1 gene:CADAFLAG00010931 transcript:CADAFLAT00010931 description: Malate synthase	55.49	31.17	1	14	14	16	539
CADAFLAP0000494	pep:known supercontig:JCVI-afl1-v2.0:EQ963475:2095220:2099094:-1 gene:CADAFLAG00004946 transcript:CADAFLAT00004946 description: Pyruvate carboxylase	55.23	17.77	1	14	14	14	1193
CADAFLAP0000500	pep:known supercontig:JCVI-afl1-v2.0:EQ963475:2257383:2260906:-1 gene:CADAFLAG00005003 transcript:CADAFLAT00005003 description: ATP dependent RNA helicase (Dob1), putative	46.89	16.94	1	14	14	14	1080
CADAFLAP0000044	pep:known supercontig:JCVI-afl1-v2.0:EQ963472:1149017:1152795:1 gene:CADAFLAG00000442 transcript:CADAFLAT00000442 description: Translation elongation factor eEF-3, putative	46.23	22.40	1	13	13	15	1067
CADAFLAP0000244	pep:known supercontig:JCVI-afl1-v2.0:EQ963473:2146910:2148707:-1 gene:CADAFLAG00002446 transcript:CADAFLAT00002446 description: Phosphoglucomutase PgmA	44.92	34.23	1	13	13	13	555
CADAFLAP0000482	pep:known supercontig:JCVI-afl1-v2.0:EQ963475:1801208:1804231:1 gene:CADAFLAG00004824 transcript:CADAFLAT00004824 description: Putative uncharacterized protein	46.44	13.68	1	12	12	14	972
CADAFLAP0001207	pep:known supercontig:JCVI-afl1-v2.0:EQ963484:1621938:1624159:1 gene:CADAFLAG00012077 transcript:CADAFLAT00012077 description: Eukaryotic translation initiation factor subunit eIF2A, putative	40.66	27.42	1	12	12	12	682
CADAFLAP0001010	pep:known supercontig:JCVI-afl1-v2.0:EQ963481:2006011:2007476:-1 gene:CADAFLAG00010106 transcript:CADAFLAT00010106 description: Replication protein A 70 kDa DNA-binding subunit	48.69	36.00	1	11	11	13	375
CADAFLAP0000494	pep:known supercontig:JCVI-afl1-v2.0:EQ963475:2101733:2110515:-1 gene:CADAFLAG00004947 transcript:CADAFLAT00004947 description: Clathrin heavy chain	43.69	11.46	1	11	11	11	1762
CADAFLAP0001207	pep:known supercontig:JCVI-afl1-v2.0:EQ963484:1615136:1616293:1 gene:CADAFLAG00012073 transcript:CADAFLAT00012073 description: Sister chromatid cohesion acetyltransferase Eco1, putative	42.13	32.21	1	11	11	13	385
CADAFLAP0001052	pep:known supercontig:JCVI-afl1-v2.0:EQ963482:1155676:1158179:-1 gene:CADAFLAG00010521 transcript:CADAFLAT00010521 description: Vacuolar dynamin-like GTPase VpsA, putative	37.92	24.06	1	11	11	11	694
CADAFLAP0001101	pep:known supercontig:JCVI-afl1-v2.0:EQ963483:679089:680918:-1 gene:CADAFLAG00011019 transcript:CADAFLAT00011019 description: Conserved lysine-rich protein, putative	33.92	30.67	1	11	11	12	538
CADAFLAP0000580	pep:known supercontig:JCVI-afl1-v2.0:EQ963476:1776812:1778528:1 gene:CADAFLAG00005809 transcript:CADAFLAT00005809 description: Glucose-6-phosphate isomerase	57.54	30.56	1	10	10	14	553
CADAFLAP0000747	pep:known supercontig:JCVI-afl1-v2.0:EQ963478:1359100:1361580:-1 gene:CADAFLAG00007478 transcript:CADAFLAT00007478 description: Putative uncharacterized protein	46.53	20.03	1	10	10	12	644
CADAFLAP0001006	pep:known supercontig:JCVI-afl1-v2.0:EQ963481:1890958:1892981:1 gene:CADAFLAG00010065 transcript:CADAFLAT00010065 description: Hexokinase Kxk, putative	43.37	32.65	1	10	10	12	490
CADAFLAP0000816	pep:known supercontig:JCVI-afl1-v2.0:EQ963479:837390:838972:-1 gene:CADAFLAG00008160 transcript:CADAFLAT00008160 description: Elongation factor Tu	42.55	34.47	1	10	10	11	441
CADAFLAP0001172	pep:known supercontig:JCVI-afl1-v2.0:EQ963484:724131:727328:-1 gene:CADAFLAG00011721 transcript:CADAFLAT00011721 description: Putative uncharacterized protein	40.41	18.23	1	10	10	10	1048
CADAFLAP0000208	pep:known supercontig:JCVI-afl1-v2.0:EQ963473:1126458:1128842:-1 gene:CADAFLAG00002082 transcript:CADAFLAT00002082 description: Acetyl-coenzyme A synthetase FacA	27.99	13.80	1	10	10	11	710
CADAFLAP0001092	pep:known supercontig:JCVI-afl1-v2.0:EQ963483:390873:392389:-1 gene:CADAFLAG00010921 transcript:CADAFLAT00010921 description: Citrate synthase	27.43	29.12	1	10	10	10	467
CADAFLAP0001208	pep:known supercontig:JCVI-afl1-v2.0:EQ963484:1638349:1640768:1 gene:CADAFLAG00012083 transcript:CADAFLAT00012083 description: Histone promoter control protein, putative	54.18	19.44	1	9	9	11	787
CADAFLAP0000185	pep:known supercontig:JCVI-afl1-v2.0:EQ963473:491941:493962:-1 gene:CADAFLAG00001859 transcript:CADAFLAT00001859 description: Putative uncharacterized protein	50.45	26.00	1	9	9	12	673
CADAFLAP0000045	pep:known supercontig:JCVI-afl1-v2.0:EQ963472:1182111:1184111:1 gene:CADAFLAG00000450 transcript:CADAFLAT00000450 description: Pyruvate dehydrogenase complex, dihydrolipoamide acetyltransferase	37.96	32.16	1	9	9	9	485
CADAFLAP0000547	pep:known supercontig:JCVI-afl1-v2.0:EQ963476:876078:877741:1 gene:CADAFLAG00005479 transcript:CADAFLAT00005479 description: Chromosome segregation protein (Pcs1), putative	36.54	29.48	1	9	9	10	519
CADAFLAP0001010	pep:known supercontig:JCVI-afl1-v2.0:EQ963481:1995326:1996965:-1 gene:CADAFLAG00010101 transcript:CADAFLAT00010101 description: Putative uncharacterized protein	35.32	27.31	1	9	9	9	509
CADAFLAP0000852	pep:known supercontig:JCVI-afl1-v2.0:EQ963479:1865471:1866979:-1 gene:CADAFLAG00008523 transcript:CADAFLAT00008523 description: UTP-glucose-1-phosphate uridylyltransferase Ugp1, putative	33.98	24.10	1	9	9	9	502
CADAFLAP0000572	pep:known supercontig:JCVI-afl1-v2.0:EQ963476:1524272:1525447:-1 gene:CADAFLAG00005725 transcript:CADAFLAT00005725 description: Mannitol-1-phosphate dehydrogenase	31.52	31.71	1	9	9	9	391
CADAFLAP0000494	pep:known supercontig:JCVI-afl1-v2.0:EQ963475:2114609:2116687:-1 gene:CADAFLAG00004949 transcript:CADAFLAT00004949 description: Phosphoribosylaminoimidazolecarboxamide formyltransferase/IMP	24.24	22.52	1	9	9	9	595
CADAFLAP0000860	pep:known supercontig:JCVI-afl1-v2.0:EQ963480:36079:41772:1 gene:CADAFLAG00008603 transcript:CADAFLAT00008603 description: Fatty acid synthase alpha subunit FasA	23.54	6.79	1	9	9	9	1857
CADAFLAP0000062	pep:known supercontig:JCVI-afl1-v2.0:EQ963472:1631080:1633180:-1 gene:CADAFLAG00000624 transcript:CADAFLAT00000624 description: Fumarate reductase Osm1, putative	21.50	17.22	1	9	9	9	633
CADAFLAP0000355	pep:known supercontig:JCVI-afl1-v2.0:EQ963474:1042720:1044510:1 gene:CADAFLAG00003559 transcript:CADAFLAT00003559 description: Putative uncharacterized protein	32.23	20.24	1	8	8	8	573
CADAFLAP0000928	pep:known supercontig:JCVI-afl1-v2.0:EQ963480:1839860:1842354:1 gene:CADAFLAG00009281 transcript:CADAFLAT00009281 description: Hsp70 chaperone Hsp88	27.00	14.87	1	8	8	9	713
CADAFLAP0000887	pep:known supercontig:JCVI-afl1-v2.0:EQ963480:744729:746663:1 gene:CADAFLAG00008873 transcript:CADAFLAT00008873 description: Septin AspA, putative	26.84	28.50	1	8	8	8	379
CADAFLAP0001008	pep:known supercontig:JCVI-afl1-v2.0:EQ963481:1962393:1964153:-1 gene:CADAFLAG00010089 transcript:CADAFLAT00010089 description: Protein disulfide isomerase Pdi1, putative	25.08	21.94	1	8	8	8	515
CADAFLAP0000479	pep:known supercontig:JCVI-afl1-v2.0:EQ963475:1707091:1710083:-1 gene:CADAFLAG00004794 transcript:CADAFLAT00004794 description: Aminopeptidase	24.44	11.55	1	8	8	8	961
CADAFLAP0000141	pep:known supercontig:JCVI-afl1-v2.0:EQ963472:3778580:3780973:-1 gene:CADAFLAG00001419 transcript:CADAFLAT00001419 description: Isocitrate dehydrogenase	22.73	19.84	1	8	8	8	499
CADAFLAP0000062	pep:known supercontig:JCVI-afl1-v2.0:EQ963472:1615903:1617653:-1 gene:CADAFLAG00000620 transcript:CADAFLAT00000620 description: Acetyl-coA hydrolase Ach1, putative	39.98	27.24	1	7	7	10	525
CADAFLAP0000367	pep:known supercontig:JCVI-afl1-v2.0:EQ963474:1386461:1388119:-1 gene:CADAFLAG00003677 transcript:CADAFLAT00003677 description: Spermidine synthase	39.71	34.71	1	7	7	9	340

CADAFLAP0000605	pep:known supercontig:JCVI-afl1-v2.0:EQ963476:2499774:2501537:1 gene:CADAFLAG00006051 transcript:CADAFLAT00006051 description: Cystathionine beta-synthase, putative	33.62	24.39	1	7	7	7	529
CADAFLAP0000881	pep:known supercontig:JCVI-afl1-v2.0:EQ963480:570126:572169:-1 gene:CADAFLAG00008812 transcript:CADAFLAT00008812 description: Fumarate hydratase, putative	33.39	23.10	1	7	7	8	554
CADAFLAP0000563	pep:known supercontig:JCVI-afl1-v2.0:EQ963476:1282856:1284624:1 gene:CADAFLAG00005638 transcript:CADAFLAT00005638 description: Dihydrolipoyl dehydrogenase	33.32	22.46	1	7	7	8	512
CADAFLAP0000301	pep:known supercontig:JCVI-afl1-v2.0:EQ963473:3702311:3703864:1 gene:CADAFLAG00003019 transcript:CADAFLAT00003019 description: Alanine aminotransferase, putative	33.27	27.11	1	7	7	8	498
CADAFLAP0000504	pep:known supercontig:JCVI-afl1-v2.0:EQ963475:2384512:2385823:-1 gene:CADAFLAG00005046 transcript:CADAFLAT00005046 description: GPI-anchored cell wall organization protein Ecm33	32.28	20.60	1	7	7	9	398
CADAFLAP0000013	pep:known supercontig:JCVI-afl1-v2.0:EQ963472:324198:324959:1 gene:CADAFLAG00000131 transcript:CADAFLAT00000131 description: Tropomyosin, putative	32.06	50.93	1	7	7	8	161
CADAFLAP0000211	pep:known supercontig:JCVI-afl1-v2.0:EQ963473:1209310:1211142:-1 gene:CADAFLAG00002110 transcript:CADAFLAT00002110 description: Saccharopine dehydrogenase Lys9, putative	27.77	27.56	1	7	7	7	450
CADAFLAP0001033	pep:known supercontig:JCVI-afl1-v2.0:EQ963482:579999:582032:1 gene:CADAFLAG00010333 transcript:CADAFLAT00010333 description: Curved DNA-binding protein (42 kDa protein)	26.45	20.39	1	7	7	7	407
CADAFLAP0000454	pep:known supercontig:JCVI-afl1-v2.0:EQ963475:1032320:1033870:1 gene:CADAFLAG00004546 transcript:CADAFLAT00004546 description: Argininosuccinate synthase	25.84	22.78	1	7	7	8	417
CADAFLAP0000284	pep:known supercontig:JCVI-afl1-v2.0:EQ963473:3215149:3217086:1 gene:CADAFLAG00002845 transcript:CADAFLAT00002845 description: Transcription factor RfeD, putative	23.78	21.26	1	7	7	7	494
CADAFLAP0000743	pep:known supercontig:JCVI-afl1-v2.0:EQ963478:1225665:1226685:-1 gene:CADAFLAG00007432 transcript:CADAFLAT00007432 description: ATP synthase oligomycin sensitivity conferral protein, putative	21.06	28.76	1	7	7	7	226
CADAFLAP0000812	pep:known supercontig:JCVI-afl1-v2.0:EQ963479:760440:763107:1 gene:CADAFLAG00008125 transcript:CADAFLAT00008125 description: Carnitine acetyl transferase	20.53	12.14	1	7	7	7	799
CADAFLAP0000303	pep:known supercontig:JCVI-afl1-v2.0:EQ963473:3770596:3772369:1 gene:CADAFLAG00003039 transcript:CADAFLAT00003039 description: PH domain protein	20.23	23.62	1	7	7	7	525
CADAFLAP0000852	pep:known supercontig:JCVI-afl1-v2.0:EQ963479:1855567:1858665:1 gene:CADAFLAG00008520 transcript:CADAFLAT00008520 description: Involucrin, putative	39.27	17.28	1	6	6	9	677
CADAFLAP0000282	pep:known supercontig:JCVI-afl1-v2.0:EQ963473:3150116:3152402:1 gene:CADAFLAG00002820 transcript:CADAFLAT00002820 description: WD repeat protein	33.43	23.68	1	6	6	7	532
CADAFLAP0000889	pep:known supercontig:JCVI-afl1-v2.0:EQ963480:806443:808343:1 gene:CADAFLAG00008895 transcript:CADAFLAT00008895 description: Inosine-5'-monophosphate dehydrogenase	32.23	23.63	1	6	6	6	546
CADAFLAP0000465	pep:known supercontig:JCVI-afl1-v2.0:EQ963475:1327837:1328832:-1 gene:CADAFLAG00004653 transcript:CADAFLAT00004653 description: Possible replication factor-a protein	31.81	33.33	1	6	6	8	276
CADAFLAP0000991	pep:known supercontig:JCVI-afl1-v2.0:EQ963481:1452511:1454454:-1 gene:CADAFLAG00009911 transcript:CADAFLAT00009911 description: Delta-1-pyrroline-5-carboxylate dehydrogenase PrnC	27.24	23.34	1	6	6	6	574
CADAFLAP0000214	pep:known supercontig:JCVI-afl1-v2.0:EQ963473:1347555:1348597:1 gene:CADAFLAG00002148 transcript:CADAFLAT00002148 description: Glycerol dehydrogenase (GldB), putative	25.00	32.31	1	6	6	6	325
CADAFLAP0000210	pep:known supercontig:JCVI-afl1-v2.0:EQ963473:1197615:1198758:-1 gene:CADAFLAG00002106 transcript:CADAFLAT00002106 description: Fructose-1,6-bisphosphatase Fbp1, putative	24.97	26.76	1	6	6	6	355
CADAFLAP0001076	pep:known supercontig:JCVI-afl1-v2.0:EQ963482:1879404:1881039:-1 gene:CADAFLAG00010767 transcript:CADAFLAT00010767 description: Homocysteine synthase CysD	24.68	22.53	1	6	6	6	435
CADAFLAP0001030	pep:known supercontig:JCVI-afl1-v2.0:EQ963482:501187:504449:-1 gene:CADAFLAG00010303 transcript:CADAFLAT00010303 description: Cell division control protein Cdc48	24.60	10.23	1	6	6	7	821
CADAFLAP0000209	pep:known supercontig:JCVI-afl1-v2.0:EQ963473:1149241:1150080:-1 gene:CADAFLAG00002090 transcript:CADAFLAT00002090 description: Cell division control protein Cdc31, putative	24.40	44.18	1	6	6	6	249
CADAFLAP0001000	pep:known supercontig:JCVI-afl1-v2.0:EQ963481:1693935:1695761:1 gene:CADAFLAG00010002 transcript:CADAFLAT00010002 description: 1,3-beta-glucanosyltransferase, putative	23.32	15.99	1	6	6	6	544
CADAFLAP0000305	pep:known supercontig:JCVI-afl1-v2.0:EQ963473:3803535:3805320:1 gene:CADAFLAG00003053 transcript:CADAFLAT00003053 description: Putative uncharacterized protein	22.11	14.36	1	6	6	6	564
CADAFLAP0000165	pep:known supercontig:JCVI-afl1-v2.0:EQ963472:4442484:4444810:-1 gene:CADAFLAG00001659 transcript:CADAFLAT00001659 description: Glycerol-3-phosphate dehydrogenase, mitochondrial	21.71	13.80	1	6	6	6	710
CADAFLAP0000706	pep:known supercontig:JCVI-afl1-v2.0:EQ963478:237548:238102:1 gene:CADAFLAG00007065 transcript:CADAFLAT00007065 description: Putative uncharacterized protein	21.55	50.45	1	6	6	7	111
CADAFLAP0000840	pep:known supercontig:JCVI-afl1-v2.0:EQ963479:1516901:1517936:-1 gene:CADAFLAG00008409 transcript:CADAFLAT00008409 description: Glycerol dehydrogenase Gcy1, putative	21.47	34.56	1	6	6	6	298
CADAFLAP0001118	pep:known supercontig:JCVI-afl1-v2.0:EQ963483:1159648:1161930:-1 gene:CADAFLAG00011184 transcript:CADAFLAT00011184 description: Glutamine synthetase	20.96	25.20	1	6	6	6	377
CADAFLAP0001085	pep:known supercontig:JCVI-afl1-v2.0:EQ963483:190843:192496:-1 gene:CADAFLAG00010853 transcript:CADAFLAT00010853 description: Succinyl-CoA synthetase alpha subunit, putative	20.05	22.42	1	6	6	8	330
CADAFLAP0000063	pep:known supercontig:JCVI-afl1-v2.0:EQ963472:1661701:1663028:-1 gene:CADAFLAG00000634 transcript:CADAFLAT00000634 description: Mitochondrial ATPase subunit ATP4, putative	19.03	29.63	1	6	6	6	243
CADAFLAP0000374	pep:known supercontig:JCVI-afl1-v2.0:EQ963474:1563476:1566818:1 gene:CADAFLAG00003740 transcript:CADAFLAT00003740 description: Glycogen phosphorylase GlpV/Gph1, putative	17.37	12.06	1	6	6	6	879
CADAFLAP0000573	pep:known supercontig:JCVI-afl1-v2.0:EQ963476:1559843:1561676:1 gene:CADAFLAG00005736 transcript:CADAFLAT00005736 description: Uricase	17.24	22.85	1	6	6	6	302
CADAFLAP0001343	pep:known supercontig:JCVI-afl1-v2.0:EQ963487:256722:258530:-1 gene:CADAFLAG00013435 transcript:CADAFLAT00013435 description: Succinyl-CoA synthetase beta subunit, putative	16.26	14.05	1	6	6	6	491
CADAFLAP0001250	pep:known supercontig:JCVI-afl1-v2.0:EQ963485:949613:950359:-1 gene:CADAFLAG00012505 transcript:CADAFLAT00012505 description: Glutathione-S-transferase theta, GST, putative	15.21	39.52	1	6	6	7	210
CADAFLAP0000526	pep:known supercontig:JCVI-afl1-v2.0:EQ963476:350558:351379:1 gene:CADAFLAG00005268 transcript:CADAFLAT00005268 description: Cell wall serine-threonine-rich galactomannoprotein Mp1	29.99	34.07	1	5	5	6	273
CADAFLAP0000173	pep:known supercontig:JCVI-afl1-v2.0:EQ963473:199571:199957:1 gene:CADAFLAG00001737 transcript:CADAFLAT00001737 description: Putative uncharacterized protein	26.85	55.47	1	5	5	6	128
CADAFLAP0000103	pep:known supercontig:JCVI-afl1-v2.0:EQ963472:2741596:2742394:1 gene:CADAFLAG00001031 transcript:CADAFLAT00001031 description: TCTP family protein	24.71	40.46	1	5	5	6	173
CADAFLAP0000214	pep:known supercontig:JCVI-afl1-v2.0:EQ963473:1342058:1342917:1 gene:CADAFLAG00002146 transcript:CADAFLAT00002146 description: Peptidyl-prolyl cis-trans isomerase	21.75	33.73	1	5	5	7	166
CADAFLAP0000850	pep:known supercontig:JCVI-afl1-v2.0:EQ963479:1791721:1793654:-1 gene:CADAFLAG00008502 transcript:CADAFLAT00008502 description: Acetamidase, putative	20.75	16.58	1	5	5	5	579
CADAFLAP0000061	pep:known supercontig:JCVI-afl1-v2.0:EQ963472:1603161:1605021:-1 gene:CADAFLAG00000614 transcript:CADAFLAT00000614 description: Probable beta-glucosidase btgE	19.42	12.62	1	5	5	5	602
CADAFLAP0001076	pep:known supercontig:JCVI-afl1-v2.0:EQ963482:1866457:1868062:1 gene:CADAFLAG00010763 transcript:CADAFLAT00010763 description: Ubiquinol-cytochrome C reductase complex core protein 2, putative	18.70	27.04	1	5	5	5	318
CADAFLAP0000592	pep:known supercontig:JCVI-afl1-v2.0:EQ963476:2078558:2081418:-1 gene:CADAFLAG00005925 transcript:CADAFLAT00005925 description: Probable dipeptidyl-peptidase 5	18.65	9.44	1	5	5	6	731
CADAFLAP0000582	pep:known supercontig:JCVI-afl1-v2.0:EQ963476:1814509:1816071:-1 gene:CADAFLAG00005829 transcript:CADAFLAT00005829 description: Aspartate aminotransferase	18.23	18.06	1	5	5	5	443
CADAFLAP0000442	pep:known supercontig:JCVI-afl1-v2.0:EQ963475:714186:715377:-1 gene:CADAFLAG00004424 transcript:CADAFLAT00004424 description: Aldehyde reductase (AKR1), putative	17.84	30.12	1	5	5	5	332
CADAFLAP0000358	pep:known supercontig:JCVI-afl1-v2.0:EQ963474:1115753:1118605:1 gene:CADAFLAG00003581 transcript:CADAFLAT00003581 description: Acetylglutamate kinase, putative	17.55	7.17	1	5	5	5	906
CADAFLAP0000503	pep:known supercontig:JCVI-afl1-v2.0:EQ963475:2359587:2360927:-1 gene:CADAFLAG00005038 transcript:CADAFLAT00005038 description: Outer mitochondrial membrane protein porin	17.20	15.90	1	5	5	6	346
CADAFLAP0000142	pep:known supercontig:JCVI-afl1-v2.0:EQ963472:3782147:3785518:-1 gene:CADAFLAG00001420 transcript:CADAFLAT00001420 description: C1 tetrahydrofolate synthase, putative	17.15	7.10	1	5	5	5	1042
CADAFLAP0000310	pep:known supercontig:JCVI-afl1-v2.0:EQ963473:3958971:3959867:-1 gene:CADAFLAG00003105 transcript:CADAFLAT00003105 description: Rho GTPase Rho1	16.40	39.90	1	5	5	5	193
CADAFLAP0000716	pep:known supercontig:JCVI-afl1-v2.0:EQ963478:472774:474322:-1 gene:CADAFLAG00007163 transcript:CADAFLAT00007163 description: Arp2/3 complex subunit (Arp3), putative	15.97	21.48	1	5	5	5	433
CADAFLAP0001077	pep:known supercontig:JCVI-afl1-v2.0:EQ963482:1903512:1906994:-1 gene:CADAFLAG00010777 transcript:CADAFLAT00010777 description: Aminopeptidase, putative	14.04	9.76	1	5	5	5	881

CADAFLAP0001072	pep:known supercontig:JCVI-afl1-v2.0:EQ963482:1782647:1785024:-1 gene:CADAFLAG00010727 transcript:CADAFLAT00010727 description: Spindle pole body associated protein SnaD, putative	13.80	8.24	1	5	5	5	704
CADAFLAP0000753	pep:known supercontig:JCVI-afl1-v2.0:EQ963478:1531723:1532637:-1 gene:CADAFLAG00007538 transcript:CADAFLAT00007538 description: GrpE protein homolog	13.41	24.70	1	5	5	5	247
CADAFLAP0001112	pep:known supercontig:JCVI-afl1-v2.0:EQ963483:994025:997698:1 gene:CADAFLAG00011127 transcript:CADAFLAT00011127 description: Putative uncharacterized protein	12.70	6.56	1	5	5	5	899
CADAFLAP0000480	pep:known supercontig:JCVI-afl1-v2.0:EQ963475:1739599:1740743:-1 gene:CADAFLAG00004803 transcript:CADAFLAT00004803 description: Cytochrome c peroxidase Ccp1, putative	11.88	17.68	1	5	5	5	362
CADAFLAP0001181	pep:known supercontig:JCVI-afl1-v2.0:EQ963484:968882:970564:1 gene:CADAFLAG00011816 transcript:CADAFLAT00011816 description: Proteasome regulatory particle subunit (RpnE), putative	8.09	10.44	1	5	5	5	498
CADAFLAP0000906	pep:known supercontig:JCVI-afl1-v2.0:EQ963480:1291839:1292342:-1 gene:CADAFLAG00009067 transcript:CADAFLAT00009067 description: SsDNA binding protein Ssb3, putative	35.26	50.41	1	4	4	7	123
CADAFLAP0001163	pep:known supercontig:JCVI-afl1-v2.0:EQ963484:473407:474791:-1 gene:CADAFLAG00011638 transcript:CADAFLAT00011638 description: Superoxide dismutase	24.21	51.95	1	4	4	5	154
CADAFLAP0000256	pep:known supercontig:JCVI-afl1-v2.0:EQ963473:2456211:2460173:-1 gene:CADAFLAG00002560 transcript:CADAFLAT00002560 description: Clustered mitochondria protein homolog	23.83	7.69	1	4	4	5	1248
CADAFLAP0000405	pep:known supercontig:JCVI-afl1-v2.0:EQ963474:2432289:2433508:1 gene:CADAFLAG00004055 transcript:CADAFLAT00004055 description: Aminotransferase, class V, putative	19.30	11.66	1	4	4	5	386
CADAFLAP0000042	pep:known supercontig:JCVI-afl1-v2.0:EQ963472:1105368:1106555:1 gene:CADAFLAG00000427 transcript:CADAFLAT00000427 description: Electron transfer flavoprotein alpha subunit, putative	18.82	18.16	1	4	4	4	347
CADAFLAP0000078	pep:known supercontig:JCVI-afl1-v2.0:EQ963472:2077201:2078611:-1 gene:CADAFLAG00000784 transcript:CADAFLAT00000784 description: ATP synthase subunit gamma	18.46	17.51	1	4	4	5	297
CADAFLAP0000358	pep:known supercontig:JCVI-afl1-v2.0:EQ963474:1129063:1130937:1 gene:CADAFLAG00003586 transcript:CADAFLAT00003586 description: Mitochondrial processing peptidase beta subunit, putative	18.31	17.12	1	4	4	4	479
CADAFLAP0001216	pep:known supercontig:JCVI-afl1-v2.0:EQ963485:51761:52235:1 gene:CADAFLAG00012165 transcript:CADAFLAT00012165 description: Putative uncharacterized protein	17.98	44.68	1	4	4	4	141
CADAFLAP0000413	pep:known supercontig:JCVI-afl1-v2.0:EQ963474:2663109:2665249:-1 gene:CADAFLAG00004139 transcript:CADAFLAT00004139 description: Cupin domain protein	17.79	10.92	1	4	4	5	641
CADAFLAP0000231	pep:known supercontig:JCVI-afl1-v2.0:EQ963473:1782398:1783642:-1 gene:CADAFLAG00002319 transcript:CADAFLAT00002319 description: NADH-cytochrome b5 reductase, putative	17.77	22.29	1	4	4	4	323
CADAFLAP0000130	pep:known supercontig:JCVI-afl1-v2.0:EQ963472:3514361:3517540:-1 gene:CADAFLAG00001304 transcript:CADAFLAT00001304 description: RNA binding protein, putative	17.26	8.07	1	4	4	4	1041
CADAFLAP0001105	pep:known supercontig:JCVI-afl1-v2.0:EQ963483:776604:778253:1 gene:CADAFLAG00011052 transcript:CADAFLAT00011052 description: Betaine-aldehyde dehydrogenase, putative	16.97	14.29	1	4	4	4	483
CADAFLAP0000329	pep:known supercontig:JCVI-afl1-v2.0:EQ963474:339614:340434:1 gene:CADAFLAG00003295 transcript:CADAFLAT00003295 description: Putative uncharacterized protein	16.33	72.48	1	4	4	4	109
CADAFLAP0001075	pep:known supercontig:JCVI-afl1-v2.0:EQ963482:1848914:1850659:1 gene:CADAFLAG00010758 transcript:CADAFLAT00010758 description: NTF2 and RRM domain protein	15.78	11.50	1	4	4	6	539
CADAFLAP0000214	pep:known supercontig:JCVI-afl1-v2.0:EQ963473:1344392:1345852:-1 gene:CADAFLAG00002147 transcript:CADAFLAT00002147 description: Phosphatidyl synthase	15.74	10.29	1	4	4	4	486
CADAFLAP0001164	pep:known supercontig:JCVI-afl1-v2.0:EQ963484:488554:490163:1 gene:CADAFLAG00011640 transcript:CADAFLAT00011640 description: Autophagic serine protease Alp2	15.65	13.54	1	4	4	4	495
CADAFLAP0000702	pep:known supercontig:JCVI-afl1-v2.0:EQ963478:107244:108734:1 gene:CADAFLAG00007020 transcript:CADAFLAT00007020 description: Eukaryotic translation initiation factor 3 subunit M	15.62	15.88	1	4	4	4	466
CADAFLAP0001041	pep:known supercontig:JCVI-afl1-v2.0:EQ963482:799044:801434:1 gene:CADAFLAG00010410 transcript:CADAFLAT00010410 description: Translation initiation factor 4B	15.57	13.39	1	4	4	4	493
CADAFLAP0000202	pep:known supercontig:JCVI-afl1-v2.0:EQ963473:981877:983504:1 gene:CADAFLAG00002028 transcript:CADAFLAT00002028 description: Homocitrate synthase	15.30	12.53	1	4	4	4	463
CADAFLAP0000237	pep:known supercontig:JCVI-afl1-v2.0:EQ963473:1966313:1969575:1 gene:CADAFLAG00002378 transcript:CADAFLAT00002378 description: Eukaryotic translation initiation factor 3 subunit A	14.82	6.49	1	4	4	4	1047
CADAFLAP0000727	pep:known supercontig:JCVI-afl1-v2.0:EQ963478:779419:781271:1 gene:CADAFLAG00007278 transcript:CADAFLAT00007278 description: Secretory pathway gdp dissociation inhibitor	14.81	14.35	1	4	4	4	467
CADAFLAP0000151	pep:known supercontig:JCVI-afl1-v2.0:EQ963472:4054449:4055909:-1 gene:CADAFLAG00001519 transcript:CADAFLAT00001519 description: Histone deacetylase HosA	14.49	16.46	1	4	4	4	486
CADAFLAP0001070	pep:known supercontig:JCVI-afl1-v2.0:EQ963482:1718556:1720986:-1 gene:CADAFLAG00010702 transcript:CADAFLAT00010702 description: Glutamyl-tRNA synthetase	14.24	8.98	1	4	4	4	746
CADAFLAP0000147	pep:known supercontig:JCVI-afl1-v2.0:EQ963472:3932110:3934557:-1 gene:CADAFLAG00001478 transcript:CADAFLAT00001478 description: Cell wall protein, putative	13.96	6.01	1	4	4	5	815
CADAFLAP0000115	pep:known supercontig:JCVI-afl1-v2.0:EQ963472:3083165:3086306:-1 gene:CADAFLAG00001155 transcript:CADAFLAT00001155 description: Importin beta-1 subunit	13.65	6.88	1	4	4	4	872
CADAFLAP0001180	pep:known supercontig:JCVI-afl1-v2.0:EQ963484:943553:945457:-1 gene:CADAFLAG00011804 transcript:CADAFLAT00011804 description: ATP sulphurylase	13.45	10.87	1	4	4	4	515
CADAFLAP0000900	pep:known supercontig:JCVI-afl1-v2.0:EQ963480:1126513:1129940:1 gene:CADAFLAG00009007 transcript:CADAFLAT00009007 description: Ran-specific GTPase-activating protein 1, putative	13.29	12.53	1	4	4	4	471
CADAFLAP0001052	pep:known supercontig:JCVI-afl1-v2.0:EQ963482:1158813:1161211:-1 gene:CADAFLAG00010522 transcript:CADAFLAT00010522 description: Vacuolar ATP synthase catalytic subunit A, putative	12.56	7.88	1	4	4	4	698
CADAFLAP0000713	pep:known supercontig:JCVI-afl1-v2.0:EQ963478:393076:393514:-1 gene:CADAFLAG00007133 transcript:CADAFLAT00007133 description: Thioredoxin	12.32	56.36	1	4	4	4	110
CADAFLAP0000551	pep:known supercontig:JCVI-afl1-v2.0:EQ963476:970387:973645:-1 gene:CADAFLAG00005511 transcript:CADAFLAT00005511 description: Glycine dehydrogenase	12.14	4.70	1	4	4	4	1064
CADAFLAP0000723	pep:known supercontig:JCVI-afl1-v2.0:EQ963478:656101:659152:-1 gene:CADAFLAG00007231 transcript:CADAFLAT00007231 description: Coatomer subunit gamma	11.40	7.76	1	4	4	4	915
CADAFLAP0000447	pep:known supercontig:JCVI-afl1-v2.0:EQ963475:852712:854356:1 gene:CADAFLAG00004476 transcript:CADAFLAT00004476 description: Putative uncharacterized protein	11.19	10.09	1	4	4	4	456
CADAFLAP0000391	pep:known supercontig:JCVI-afl1-v2.0:EQ963474:2040098:2040947:-1 gene:CADAFLAG00003913 transcript:CADAFLAT00003913 description: Glycolipid transfer protein HET-C2, putative	9.93	21.36	1	4	4	4	206
CADAFLAP0001089	pep:known supercontig:JCVI-afl1-v2.0:EQ963483:323423:324843:1 gene:CADAFLAG00010894 transcript:CADAFLAT00010894 description: Acyl-CoA dehydrogenase family protein	9.38	12.37	1	4	4	4	388
CADAFLAP0000156	pep:known supercontig:JCVI-afl1-v2.0:EQ963472:4189783:4191067:-1 gene:CADAFLAG00001560 transcript:CADAFLAT00001560 description: Cell wall integrity signaling protein Lsp1/Pil1, putative	8.91	13.26	1	4	4	4	347
CADAFLAP0000161	pep:known supercontig:JCVI-afl1-v2.0:EQ963472:4319148:4320367:-1 gene:CADAFLAG00001610 transcript:CADAFLAT00001610 description: Protein phosphatase 2C, putative	8.79	19.23	1	4	4	4	312
CADAFLAP0000753	pep:known supercontig:JCVI-afl1-v2.0:EQ963478:1523778:1525000:1 gene:CADAFLAG00007535 transcript:CADAFLAT00007535 description: Cytochrome c	8.66	28.57	1	4	4	4	112
CADAFLAP0000720	pep:known supercontig:JCVI-afl1-v2.0:EQ963478:598013:601062:-1 gene:CADAFLAG00007209 transcript:CADAFLAT00007209 description: Putative uncharacterized protein	7.19	5.60	1	4	4	4	964
CADAFLAP0001108	pep:known supercontig:JCVI-afl1-v2.0:EQ963483:864750:867278:1 gene:CADAFLAG00011085 transcript:CADAFLAT00011085 description: Phenylalanyl-tRNA synthetase, beta subunit	7.11	7.33	1	4	4	4	600
CADAFLAP0000398	pep:known supercontig:JCVI-afl1-v2.0:EQ963474:2246125:2248381:1 gene:CADAFLAG00003985 transcript:CADAFLAT00003985 description: Putative uncharacterized protein	6.64	8.65	1	4	4	4	532
