# Supplementary material for: The KdmB-EcoA-RpdA-SntB (KERS) chromatin regulatory complex controls development, secondary metabolism and pathogenicity in Aspergillus flavus
Source: Fungal Genet Biol. Author manuscript; Available in PMC 2024 Feb 5. (PMC10841535; doi:10.1016/j.fgb.2023.103836)
Supplement: supplemental2 [file NIHMS1938650-supplement-supplemental2.pdf]

Table S2. KdmB::3xHA interacting protein list.

Accession	Description	Score	Coverage	# Proteins	# Unique Peptides	# Peptides	# PSMs	# AAs	MW [kDa]	calc. pI
CADAFLAP00010689	pep:known supercontig:JCVI-afl1-v2.0:EQ963482:1685104:1690608:-1 gene:CADAFLAG00010689 transcript:CADAFLAT00010689 description: PHD transcription factor, putative	614.53	61.97	1	77	77	131	1704	192.4	6.32
CADAFLAP00002374	pep:known supercontig:JCVI-afl1-v2.0:EQ963473:1948258:1953549:1 gene:CADAFLAG00002374 transcript:CADAFLAT00002374 description: PHD finger and BAH domain protein (Snt2), putative	285.81	43.90	1	56	56	76	1713	188.3	8.72
CADAFLAP00003973	pep:known supercontig:JCVI-afl1-v2.0:EQ963474:2204554:2210905:-1 gene:CADAFLAG00003973 transcript:CADAFLAT00003973 description: Transcriptional corepressor of histone genes (Hir3), putative	216.88	30.84	1	47	47	57	2059	230.8	4.91
CADAFLAP00003996	pep:known supercontig:JCVI-afl1-v2.0:EQ963474:2276434:2279944:-1 gene:CADAFLAG00003996 transcript:CADAFLAT00003996 description: Histone transcription regulator Hir1, putative	232.61	53.69	1	39	39	51	1058	114.4	6.34
CADAFLAP00010727	pep:known supercontig:JCVI-afl1-v2.0:EQ963482:1782647:1785024:-1 gene:CADAFLAG00010727 transcript:CADAFLAT00010727 description: Spindle pole body associated protein SnaD, putative	67.50	35.94	1	21	21	23	704	80.1	4.89
CADAFLAP00010106	pep:known supercontig:JCVI-afl1-v2.0:EQ963481:2006011:2007476:-1 gene:CADAFLAG00010106 transcript:CADAFLAT00010106 description: Replication protein A 70 kDa DNA-binding subunit	83.08	53.07	1	18	18	21	375	42.1	5.26
CADAFLAP00000902	pep:known supercontig:JCVI-afl1-v2.0:EQ963472:2412375:2414764:1 gene:CADAFLAG00000902 transcript:CADAFLAT00000902 description: ARS binding protein Abp2, putative	123.93	32.61	1	16	16	28	779	85.1	9.04
CADAFLAP00004824	pep:known supercontig:JCVI-afl1-v2.0:EQ963475:1801208:1804231:1 gene:CADAFLAG00004824 transcript:CADAFLAT00004824 description: Putative uncharacterized protein	65.83	20.68	1	16	16	18	972	108.5	6.44
CADAFLAP00001488	pep:known supercontig:JCVI-afl1-v2.0:EQ963472:3956565:3958794:-1 gene:CADAFLAG00001488 transcript:CADAFLAT00001488 description: C6 transcription factor (OTam), putative	81.59	32.21	1	14	14	18	711	78.6	7.05
CADAFLAP00010101	pep:known supercontig:JCVI-afl1-v2.0:EQ963481:1995326:1996965:-1 gene:CADAFLAG00010101 transcript:CADAFLAT00010101 description: Putative uncharacterized protein	63.69	35.56	1	14	14	16	509	56.5	9.23
CADAFLAP00004614	pep:known supercontig:JCVI-afl1-v2.0:EQ963475:1215660:1217220:-1 gene:CADAFLAG00004614 transcript:CADAFLAT00004614 description: Aldehyde dehydrogenase AldA, putative	46.73	36.02	1	14	14	15	497	53.9	6.27
CADAFLAP00003012	pep:known supercontig:JCVI-afl1-v2.0:EQ963473:3679184:3681492:-1 gene:CADAFLAG00003012 transcript:CADAFLAT00003012 description: Phosphoenolpyruvate carboxykinase AcuF	60.23	29.17	1	12	12	16	600	66.5	6.29
CADAFLAP00002050	pep:known supercontig:JCVI-afl1-v2.0:EQ963473:1030614:1032169:-1 gene:CADAFLAG00002050 transcript:CADAFLAT00002050 description: AAA family ATPase Pontin, putative	50.53	37.42	1	12	12	13	457	49.6	6.02
CADAFLAP00008921	pep:known supercontig:JCVI-afl1-v2.0:EQ963480:874863:877102:1 gene:CADAFLAG00008921 transcript:CADAFLAT00008921 description: Histone deacetylase RpdA/Rpd3	41.32	25.84	1	12	12	12	685	75.5	4.67
CADAFLAP00010931	pep:known supercontig:JCVI-afl1-v2.0:EQ963483:417154:418937:1 gene:CADAFLAG00010931 transcript:CADAFLAT00010931 description: Malate synthase	35.51	27.64	1	12	12	13	539	61.0	8.12
CADAFLAP00003053	pep:known supercontig:JCVI-afl1-v2.0:EQ963473:3803535:3805320:1 gene:CADAFLAG00003053 transcript:CADAFLAT00003053 description: Putative uncharacterized protein	70.41	20.04	1	11	11	18	564	61.5	9.17
CADAFLAP00002780	pep:known supercontig:JCVI-afl1-v2.0:EQ963473:3053401:3056239:-1 gene:CADAFLAG00002780 transcript:CADAFLAT00002780 description: Mitochondrial aconitate hydratase, putative	47.27	20.76	1	11	11	12	785	85.5	6.93
CADAFLAP00004139	pep:known supercontig:JCVI-afl1-v2.0:EQ963474:2663109:2665249:-1 gene:CADAFLAG00004139 transcript:CADAFLAT00004139 description: Cupin domain protein	42.26	24.49	1	10	10	12	641	69.8	8.60
CADAFLAP00000885	pep:known supercontig:JCVI-afl1-v2.0:EQ963472:2377479:2379339:-1 gene:CADAFLAG00000885 transcript:CADAFLAT00000885 description: AAA family ATPase Rvb2/Reptin, putative	38.25	26.17	1	10	10	11	470	51.4	5.83
CADAFLAP00012073	pep:known supercontig:JCVI-afl1-v2.0:EQ963484:1615136:1616293:1 gene:CADAFLAG00012073 transcript:CADAFLAT00012073 description: Sister chromatid cohesion acetyltransferase Eco1, putative	37.94	35.32	1	9	9	10	385	43.0	8.62
CADAFLAP00001307	pep:known supercontig:JCVI-afl1-v2.0:EQ963472:3522383:3524067:1 gene:CADAFLAG00001307 transcript:CADAFLAT00001307 description: Pre-rRNA processing nucleolar protein Sik1, putative	35.75	25.48	1	9	9	9	522	58.1	8.88
CADAFLAP00002022	pep:known supercontig:JCVI-afl1-v2.0:EQ963473:970805:972428:-1 gene:CADAFLAG00002022 transcript:CADAFLAT00002022 description: Aspartate aminotransferase	34.79	26.81	1	9	9	9	429	47.2	8.85
CADAFLAP00006154	pep:known supercontig:JCVI-afl1-v2.0:EQ963477:230152:231761:-1 gene:CADAFLAG00006154 transcript:CADAFLAT00006154 description: Translation elongation factor eEF-1 subunit gamma, putative	33.00	28.78	1	9	9	10	410	46.4	6.73
CADAFLAP00008520	pep:known supercontig:JCVI-afl1-v2.0:EQ963479:1855567:1858665:1 gene:CADAFLAG00008520 transcript:CADAFLAT00008520 description: Involucrin, putative	71.21	18.61	1	8	8	15	677	72.7	10.48
CADAFLAP00008559	pep:known supercontig:JCVI-afl1-v2.0:EQ963479:1964301:1965959:1 gene:CADAFLAG00008559 transcript:CADAFLAT00008559 description: UDP-N-acetylglucosamine pyrophosphorylase	32.24	20.16	1	8	8	11	506	56.1	6.32
CADAFLAP00008602	pep:known supercontig:JCVI-afl1-v2.0:EQ963480:27030:33372:-1 gene:CADAFLAG00008602 transcript:CADAFLAT00008602 description: Fatty acid synthase beta subunit, putative	25.94	6.12	1	8	8	8	2092	232.0	6.00
CADAFLAP00005963	pep:known supercontig:JCVI-afl1-v2.0:EQ963476:2187122:2194103:1 gene:CADAFLAG00005963 transcript:CADAFLAT00005963 description: Acetyl-CoA carboxylase, putative	22.77	5.13	1	8	8	8	2125	236.5	6.07
CADAFLAP00009109	pep:known supercontig:JCVI-afl1-v2.0:EQ963480:1402310:1404536:1 gene:CADAFLAG00009109 transcript:CADAFLAT00009109 description: Putative uncharacterized protein	21.98	20.10	1	8	8	8	612	67.1	6.93
CADAFLAP00003945	pep:known supercontig:JCVI-afl1-v2.0:EQ963474:2120397:2123780:1 gene:CADAFLAG00003945 transcript:CADAFLAT00003945 description: AT DNA binding protein, putative	19.01	10.47	1	8	8	8	1127	127.1	5.11
CADAFLAP00012083	pep:known supercontig:JCVI-afl1-v2.0:EQ963484:1638349:1640768:1 gene:CADAFLAG00012083 transcript:CADAFLAT00012083 description: Histone promoter control protein, putative	51.63	12.71	1	7	7	12	787	81.8	9.86
CADAFLAP00004653	pep:known supercontig:JCVI-afl1-v2.0:EQ963475:1327837:1328832:-1 gene:CADAFLAG00004653 transcript:CADAFLAT00004653 description: Possible replication factor-a protein	37.23	38.41	1	7	7	9	276	28.8	5.10
CADAFLAP00002641	pep:known supercontig:JCVI-afl1-v2.0:EQ963473:2674101:2676382:1 gene:CADAFLAG00002641 transcript:CADAFLAT00002641 description: HEC/Ndc80p family protein	31.29	13.86	1	7	7	8	736	83.6	5.29
CADAFLAP00008024	pep:known supercontig:JCVI-afl1-v2.0:EQ963479:459507:460852:-1 gene:CADAFLAG00008024 transcript:CADAFLAT00008024 description: BZIP transcription factor, putative	30.30	28.52	1	7	7	9	284	30.8	6.81
CADAFLAP00004946	pep:known supercontig:JCVI-afl1-v2.0:EQ963475:2095220:2099094:-1 gene:CADAFLAG00004946 transcript:CADAFLAT00004946 description: Pyruvate carboxylase	21.66	9.39	1	7	7	7	1193	131.1	6.55
CADAFLAP00000730	pep:known supercontig:JCVI-afl1-v2.0:EQ963472:1925515:1927778:1 gene:CADAFLAG00000730 transcript:CADAFLAT00000730 description: Oligopeptidase family protein	20.75	14.48	1	7	7	7	718	78.8	6.42
CADAFLAP00004546	pep:known supercontig:JCVI-afl1-v2.0:EQ963475:1032320:1033870:1 gene:CADAFLAG00004546 transcript:CADAFLAT00004546 description: Argininosuccinate synthase	19.59	20.38	1	7	7	7	417	46.8	5.57
CADAFLAP00011019	pep:known supercontig:JCVI-afl1-v2.0:EQ963483:679089:680918:-1 gene:CADAFLAG00011019 transcript:CADAFLAT00011019 description: Conserved lysine-rich protein, putative	18.33	14.50	1	7	7	7	538	57.1	5.01
CADAFLAP00001316	pep:known supercontig:JCVI-afl1-v2.0:EQ963472:3544382:3548054:1 gene:CADAFLAG00001316 transcript:CADAFLAT00001316 description: Spindle-pole body protein (Pcp1), putative	28.95	14.61	1	6	6	8	445	50.8	9.22
CADAFLAP00002106	pep:known supercontig:JCVI-afl1-v2.0:EQ963473:1197615:1198758:-1 gene:CADAFLAG00002106 transcript:CADAFLAT00002106 description: Fructose-1,6-bisphosphatase Fbp1, putative	26.44	29.01	1	6	6	6	355	38.9	6.06
CADAFLAP00011231	pep:known supercontig:JCVI-afl1-v2.0:EQ963483:1281340:1283202:-1 gene:CADAFLAG00011231 transcript:CADAFLAT00011231 description: Isocitrate lyase	25.73	18.96	1	6	6	6	538	60.0	7.01
CADAFLAP00002554	pep:known supercontig:JCVI-afl1-v2.0:EQ963473:2435324:2436960:-1 gene:CADAFLAG00002554 transcript:CADAFLAT00002554 description: BZIP transcription factor (MeaB), putative	24.76	18.09	1	6	6	7	398	43.2	6.90
CADAFLAP00000620	pep:known supercontig:JCVI-afl1-v2.0:EQ963472:1615903:1617653:-1 gene:CADAFLAG00000620 transcript:CADAFLAT00000620 description: Acetyl-coA hydrolase Ach1, putative	21.45	20.38	1	6	6	6	525	58.0	6.74
CADAFLAP00002389	pep:known supercontig:JCVI-afl1-v2.0:EQ963473:2002547:2003611:-1 gene:CADAFLAG00002389 transcript:CADAFLAT00002389 description: 60S ribosomal protein P0	21.18	25.56	1	6	6	7	313	33.4	4.96
CADAFLAP00002478	pep:known supercontig:JCVI-afl1-v2.0:EQ963473:2228907:2231489:1 gene:CADAFLAG00002478 transcript:CADAFLAT00002478 description: Putative uncharacterized protein	20.10	9.86	1	6	6	7	740	83.3	6.34
CADAFLAP00002082	pep:known supercontig:JCVI-afl1-v2.0:EQ963473:1126458:1128842:-1 gene:CADAFLAG00002082 transcript:CADAFLAT00002082 description: Acetyl-coenzyme A synthetase FacA	18.06	8.59	1	6	6	6	710	78.9	6.71
CADAFLAP00001419	pep:known supercontig:JCVI-afl1-v2.0:EQ963472:3778580:3780973:-1 gene:CADAFLAG00001419 transcript:CADAFLAT00001419 description: Isocitrate dehydrogenase	17.86	14.63	1	6	6	6	499	55.8	8.28
CADAFLAP00003958	pep:known supercontig:JCVI-afl1-v2.0:EQ963474:2165407:2166459:-1 gene:CADAFLAG00003958 transcript:CADAFLAT00003958 description: Putative uncharacterized protein	16.21	37.36	1	6	6	7	265	29.8	8.76
CADAFLAP00005736	pep:known supercontig:JCVI-afl1-v2.0:EQ963476:1559843:1561676:1 gene:CADAFLAG00005736 transcript:CADAFLAT00005736 description: Uricase	13.92	21.52	1	6	6	6	302	34.2	7.62
CADAFLAP00008603	pep:known supercontig:JCVI-afl1-v2.0:EQ963480:36079:41772:1 gene:CADAFLAG00008603 transcript:CADAFLAT00008603 description: Fatty acid synthase alpha subunit FasA	10.47	3.23	1	6	6	6	1857	204.2	6.34
CADAFLAP00013326	pep:known supercontig:JCVI-afl1-v2.0:EQ963486:1259924:1261388:-1 gene:CADAFLAG00013326 transcript:CADAFLAT00013326 description: Acetyl-CoA acetyltransferase, putative	26.14	26.82	1	5	5	5	399	40.8	6.79

CADAFLAP00006051	pep:known supercontig:JCVI-afl1-v2.0:EQ963476:2499774:2501537:1 gene:CADAFLAG00006051 transcript:CADAFLAT00006051 description: Cystathionine beta-synthase, putative	23.34	17.39	1	5	5	5	529	57.2	6.42
CADAFLAP00008160	pep:known supercontig:JCVI-afl1-v2.0:EQ963479:837390:838972:-1 gene:CADAFLAG00008160 transcript:CADAFLAT00008160 description: Elongation factor Tu	19.67	17.23	1	5	5	6	441	48.3	6.81
CADAFLAP00002937	pep:known supercontig:JCVI-afl1-v2.0:EQ963473:3466835:3469020:-1 gene:CADAFLAG00002937 transcript:CADAFLAT00002937 description: Hsp70 chaperone BiP/Kar2, putative	17.40	9.97	1	5	5	5	672	73.4	4.94
CADAFLAP00007209	pep:known supercontig:JCVI-afl1-v2.0:EQ963478:598013:601062:-1 gene:CADAFLAG00007209 transcript:CADAFLAT00007209 description: Putative uncharacterized protein	16.43	7.68	1	5	5	5	964	104.6	10.39
CADAFLAP00006639	pep:known supercontig:JCVI-afl1-v2.0:EQ963477:1480422:1481624:1 gene:CADAFLAG00006639 transcript:CADAFLAT00006639 description: Ketol-acid reductoisomerase	14.01	19.00	1	5	5	5	400	44.4	8.70
CADAFLAP00000877	pep:known supercontig:JCVI-afl1-v2.0:EQ963472:2350263:2352028:-1 gene:CADAFLAG00000877 transcript:CADAFLAT00000877 description: Origin recognition complex subunit Orc5, putative	12.01	12.41	1	5	5	5	540	57.9	9.38
CADAFLAP00010921	pep:known supercontig:JCVI-afl1-v2.0:EQ963483:390873:392389:-1 gene:CADAFLAG00010921 transcript:CADAFLAT00010921 description: Citrate synthase	11.93	10.71	1	5	5	6	467	51.6	8.81
CADAFLAP00007538	pep:known supercontig:JCVI-afl1-v2.0:EQ963478:1531723:1532637:-1 gene:CADAFLAG00007538 transcript:CADAFLAT00007538 description: GrpE protein homolog	10.76	24.70	1	5	5	5	247	28.1	8.69
CADAFLAP00008827	pep:known supercontig:JCVI-afl1-v2.0:EQ963480:607657:609968:-1 gene:CADAFLAG00008827 transcript:CADAFLAT00008827 description: ATP dependent RNA helicase (Sub2), putative	9.62	12.47	1	5	5	5	441	49.5	5.82
CADAFLAP00003657	pep:known supercontig:JCVI-afl1-v2.0:EQ963474:1326199:1329523:1 gene:CADAFLAG00003657 transcript:CADAFLAT00003657 description: Centrin-binding protein Sfi1, putative	6.16	4.24	1	5	5	5	1085	126.8	10.33
CADAFLAP00009067	pep:known supercontig:JCVI-afl1-v2.0:EQ963480:1291839:1292342:-1 gene:CADAFLAG00009067 transcript:CADAFLAT00009067 description: SsDNA binding protein Ssb3, putative	34.55	47.97	1	4	4	7	123	13.4	6.51
CADAFLAP00012165	pep:known supercontig:JCVI-afl1-v2.0:EQ963485:51761:52235:1 gene:CADAFLAG00012165 transcript:CADAFLAT00012165 description: Putative uncharacterized protein	22.23	51.77	1	4	4	5	141	16.3	5.31
CADAFLAP00003873	pep:known supercontig:JCVI-afl1-v2.0:EQ963474:1917046:1918987:1 gene:CADAFLAG00003873 transcript:CADAFLAT00003873 description: Mis12-Mtw1 family protein	21.72	11.43	1	4	4	5	560	62.0	9.89
CADAFLAP00002146	pep:known supercontig:JCVI-afl1-v2.0:EQ963473:1342058:1342917:1 gene:CADAFLAG00002146 transcript:CADAFLAT00002146 description: Peptidyl-prolyl cis-trans isomerase	21.53	31.93	1	4	4	6	166	18.2	8.57
CADAFLAP00000131	pep:known supercontig:JCVI-afl1-v2.0:EQ963472:324198:324959:1 gene:CADAFLAG00000131 transcript:CADAFLAT00000131 description: Tropomyosin, putative	21.43	32.92	1	4	4	5	161	18.8	5.05
CADAFLAP00001079	pep:known supercontig:JCVI-afl1-v2.0:EQ963472:2875147:2876318:-1 gene:CADAFLAG00001079 transcript:CADAFLAT00001079 description: MIND kinetochore complex component Mtw1, putative	18.91	20.24	1	4	4	4	336	36.4	5.50
CADAFLAP00010065	pep:known supercontig:JCVI-afl1-v2.0:EQ963481:1890958:1892981:1 gene:CADAFLAG00010065 transcript:CADAFLAT00010065 description: Hexokinase Kxk, putative	18.64	14.90	1	4	4	5	490	54.1	5.31
CADAFLAP00010884	pep:known supercontig:JCVI-afl1-v2.0:EQ963483:289969:292077:-1 gene:CADAFLAG00010884 transcript:CADAFLAT00010884 description: C6 finger domain protein, putative	17.83	9.70	1	4	4	5	608	68.9	6.10
CADAFLAP00002306	pep:known supercontig:JCVI-afl1-v2.0:EQ963473:1745130:1746774:1 gene:CADAFLAG00002306 transcript:CADAFLAT00002306 description: Alcohol dehydrogenase, zinc-containing, putative	16.26	18.21	1	4	4	4	346	37.7	6.44
CADAFLAP00006333	pep:known supercontig:JCVI-afl1-v2.0:EQ963477:706525:708010:-1 gene:CADAFLAG00006333 transcript:CADAFLAT00006333 description: RNP domain protein	15.92	13.42	1	4	4	5	365	38.2	9.19
CADAFLAP00000159	pep:known supercontig:JCVI-afl1-v2.0:EQ963472:399440:404950:1 gene:CADAFLAG00000159 transcript:CADAFLAT00000159 description: DNA mismatch repair protein Msh3	15.27	4.11	1	4	4	4	1386	152.6	6.57
CADAFLAP00010702	pep:known supercontig:JCVI-afl1-v2.0:EQ963482:1718556:1720986:-1 gene:CADAFLAG00010702 transcript:CADAFLAT00010702 description: Glutamyl-tRNA synthetase	15.01	10.46	1	4	4	4	746	83.9	6.79
CADAFLAP00003923	pep:known supercontig:JCVI-afl1-v2.0:EQ963474:2070956:2072222:1 gene:CADAFLAG00003923 transcript:CADAFLAT00003923 description: Stomatin family protein	14.92	13.66	1	4	4	5	344	37.3	6.29
CADAFLAP00002510	pep:known supercontig:JCVI-afl1-v2.0:EQ963473:2303025:2306093:1 gene:CADAFLAG00002510 transcript:CADAFLAT00002510 description: Spindle pole body component (Alp6), putative	14.84	7.65	1	4	4	4	1006	112.6	6.42
CADAFLAP00002148	pep:known supercontig:JCVI-afl1-v2.0:EQ963473:1347555:1348597:1 gene:CADAFLAG00002148 transcript:CADAFLAT00002148 description: Glycerol dehydrogenase (GldB), putative	14.48	20.31	1	4	4	5	325	36.9	6.38
CADAFLAP00011702	pep:known supercontig:JCVI-afl1-v2.0:EQ963484:653933:656439:-1 gene:CADAFLAG00011702 transcript:CADAFLAT00011702 description: Woronin body major protein, putative	14.06	8.92	1	4	4	5	493	56.6	6.61
CADAFLAP00006772	pep:known supercontig:JCVI-afl1-v2.0:EQ963477:1838042:1838891:-1 gene:CADAFLAG00006772 transcript:CADAFLAT00006772 description: Single-stranded DNA-binding protein	13.36	31.25	1	4	4	4	144	15.6	9.98
CADAFLAP00008502	pep:known supercontig:JCVI-afl1-v2.0:EQ963479:1791721:1793654:-1 gene:CADAFLAG00008502 transcript:CADAFLAT00008502 description: Acetamidase, putative	12.78	11.57	1	4	4	4	579	63.7	8.60
CADAFLAP00010089	pep:known supercontig:JCVI-afl1-v2.0:EQ963481:1962393:1964153:-1 gene:CADAFLAG00010089 transcript:CADAFLAT00010089 description: Protein disulfide isomerase Pdi1, putative	12.73	11.07	1	4	4	4	515	56.4	4.68
CADAFLAP00000442	pep:known supercontig:JCVI-afl1-v2.0:EQ963472:1149017:1152795:1 gene:CADAFLAG00000442 transcript:CADAFLAT00000442 description: Translation elongation factor eEF-3, putative	12.01	5.25	1	4	4	5	1067	117.8	6.32
CADAFLAP00010457	pep:known supercontig:JCVI-afl1-v2.0:EQ963482:957762:961135:-1 gene:CADAFLAG00010457 transcript:CADAFLAT00010457 description: Nuclear condensin complex subunit 3, putative	11.99	4.25	1	4	4	4	1105	122.2	5.36
CADAFLAP00010829	pep:known supercontig:JCVI-afl1-v2.0:EQ963483:112627:114814:1 gene:CADAFLAG00010829 transcript:CADAFLAT00010829 description: Glycyl-tRNA synthetase	11.35	6.07	1	4	4	5	708	80.4	6.83
CADAFLAP00004949	pep:known supercontig:JCVI-afl1-v2.0:EQ963475:2114609:2116687:-1 gene:CADAFLAG00004949 transcript:CADAFLAT00004949 description: Phosphoribosylaminoimidazolecarboxamide formyltransferas	8.69	8.24	1	4	4	4	595	65.0	6.73
