# Supplementary material for: The KdmB-EcoA-RpdA-SntB (KERS) chromatin regulatory complex controls development, secondary metabolism and pathogenicity in Aspergillus flavus
Source: Fungal Genet Biol. Author manuscript; Available in PMC 2024 Feb 5. (PMC10841535; doi:10.1016/j.fgb.2023.103836)
Supplement: supplemental3 [file NIHMS1938650-supplement-supplemental3.pdf]

Table S3. List of predicted secondary metabolite gene clusters in *A. flavus* genome by Secondary Metabolite Unique Regions Finder (SMURF) tool (https://www.jcvi.org/smurf).

Cluster:1

Backbone_ge	Gene_id	Gene_positic	Chromosome	Gene_order	5'end	3'end	Gene_distan	Domain_scor	Annotated_gene_function
AFLA_00290	AFLA_00292	-2	1569	252	648825	650784	727	1	Cytochrome P450 family protein
AFLA_00290	AFLA_00291	-1	1569	251	647376	648098	546	0	hypothetical protein
AFLA_00290	AFLA_00290	0	1569	250	646830	639511	0	0	polyketide synthase, putative

Cluster:2

Backbone_ge	Gene_id	Gene_positic	Chromosome	Gene_order	5'end	3'end	Gene_distan	Domain_scor	Annotated_gene_function
AFLA_00445	AFLA_00445	0	1569	405	1115175	1131702	0	1	nonribosomal peptide synthase, putative
AFLA_00445	AFLA_00444	1	1569	404	1113845	1109751	1330	1	ABC transporter family protein
AFLA_00445	AFLA_00443	2	1569	403	1108599	1106632	1152	0	hypothetical protein
AFLA_00445	AFLA_00442	3	1569	402	1103238	1102369	3394	0	hypothetical protein
AFLA_00445	AFLA_00441	4	1569	401	1100003	1099638	2366	0	hypothetical protein
AFLA_00445	AFLA_00440	5	1569	400	1098417	1098812	826	0	Trypsin Inhibitor like cysteine rich domain containing protein
AFLA_00445	AFLA_00439	6	1569	399	1095738	1096878	1539	0	conserved hypothetical protein
AFLA_00445	AFLA_00438	7	1569	398	1095120	1092880	618	0	glycosyl hydrolase, family 43 protein
AFLA_00445	AFLA_00437	8	1569	397	1090139	1088155	2741	1	Cytochrome P450 family protein
AFLA_00445	AFLA_00436	9	1569	396	1087391	1085996	764	1	oxidoreductase, zinc-binding dehydrogenase family protein

Cluster:3

Backbone_ge	Gene_id	Gene_positic	Chromosome	Gene_order	5'end	3'end	Gene_distan	Domain_scor	Annotated_gene_function
AFLA_00532	AFLA_00536	-4	1569	495	1422063	1423775	2082	1	hypothetical protein
AFLA_00532	AFLA_00535	-3	1569	494	1419712	1419981	2147	0	hypothetical protein
AFLA_00532	AFLA_00534	-2	1569	493	1417565	1416530	1297	0	conserved hypothetical protein
AFLA_00532	AFLA_00533	-1	1569	492	1414409	1415233	631	0	hypothetical protein
AFLA_00532	AFLA_00532	0	1569	491	1413778	1405582	0	1	polyketide synthase, putative
AFLA_00532	AFLA_00531	1	1569	490	1404510	1401884	1072	0	V-type ATPase, C subunit family protein
AFLA_00532	AFLA_00530	2	1569	489	1400018	1397292	1866	0	hypothetical protein
AFLA_00532	AFLA_00529	3	1569	488	1394180	1396515	777	1	New cDNA-based gene: (AO_CDS_042706, novel, updateIDs: 352, [gene: novel_gene_57, model: novel_model_57])

Cluster:4

Backbone_ge	Gene_id	Gene_positic	Chromosome	Gene_order	5'end	3'end	Gene_distan	Domain_scor	Annotated_gene_function
AFLA_00544	AFLA_00552	-8	1569	511	1475253	1473372	650	1	hypothetical protein
AFLA_00544	AFLA_00551	-7	1569	510	1470936	1472722	6	1	hypothetical protein
AFLA_00544	AFLA_00550	-6	1569	509	1470462	1470930	2446	0	hypothetical protein
AFLA_00544	AFLA_00549	-5	1569	508	1468016	1467296	52	0	hypothetical protein
AFLA_00544	AFLA_00548	-4	1569	507	1467244	1464173	1492	0	mitochondrial nicotinamide nucleotide transhydrogenase subunit, putative
AFLA_00544	AFLA_00547	-3	1569	506	1462681	1456979	1959	0	hypothetical protein
AFLA_00544	AFLA_00546	-2	1569	505	1455020	1454392	1793	0	cytochrome P450 monooxygenase SirB-like, putative
AFLA_00544	AFLA_00545	-1	1569	504	1452599	1451477	1252	1	conserved protein; similar to SLR0865 Synechocystis sp. and other bacteria; no apparent S. cerevisiae ortholog-related
AFLA_00544	AFLA_00544	0	1569	503	1450225	1442264	0	0	nonribosomal peptide synthase, putative
AFLA_00544	AFLA_00543	1	1569	502	1439576	1440704	1560	0	conserved hypothetical protein
AFLA_00544	AFLA_00542	2	1569	501	1437084	1437629	1947	0	conserved hypothetical protein
AFLA_00544	AFLA_00541	3	1569	500	1436220	1432677	864	0	Protein kinase domain containing protein
AFLA_00544	AFLA_00540	4	1569	499	1432543	1431949	134	0	hypothetical protein
AFLA_00544	AFLA_00539	5	1569	498	1429687	1428285	2262	0	hypothetical protein
AFLA_00544	AFLA_00538	6	1569	497	1426512	1425756	1773	0	hypothetical protein
AFLA_00544	AFLA_00537	7	1569	496	1424685	1423861	1071	0	hypothetical protein
AFLA_00544	AFLA_00536	8	1569	495	1422063	1423775	86	1	hypothetical protein

Cluster:5

Backbone_ge	Gene_id	Gene_positic	Chromosome	Gene_order	5'end	3'end	Gene_distan	Domain_scor	Annotated_gene_function
AFLA_00617	AFLA_00623	-6	1569	582	1683111	1684316	458	1	1-aminocyclopropane-1-carboxylate deaminase, putative

AFLA_00617	AFLA_00622	-5	1569	581	1682653	1681265	612	0	hypothetical protein
AFLA_00617	AFLA_00621	-4	1569	580	1676835	1680653	628	0	Variant SH3 domain containing protein
AFLA_00617	AFLA_00620	-3	1569	579	1676207	1675887	1050	0	New cDNA-based gene: (AO_CDS_042706, novel, updateIDs: 417, [gene: novel_gene_73, model: novel_model_73])
AFLA_00617	AFLA_00619	-2	1569	578	1674837	1674196	2	0	hypothetical protein
AFLA_00617	AFLA_00618	-1	1569	577	1674194	1672714	1462	0	conidial pigment biosynthesis oxidase Abr1/brown 1
AFLA_00617	AFLA_00617	0	1569	576	1671252	1664602	0	0	polyketide synthetase PksP

Cluster:6

Backbone_ge	Gene_id	Gene_positic	Chromosome	Gene_order	5'end	3'end	Gene_distan	Domain_scor	Annotated_gene_function
AFLA_00877	AFLA_00877	0	1739	143	349578	333887	0	0	nonribosomal peptide synthase, putative
AFLA_00877	AFLA_00876	1	1739	142	332785	328544	1102	1	ABC transporter family protein
AFLA_00877	AFLA_00875	2	1739	141	327942	327354	602	0	hypothetical protein
AFLA_00877	AFLA_00874	3	1739	140	325659	324646	1695	1	oxidoreductase, short chain dehydrogenase/reductase family protein
AFLA_00877	AFLA_00873	4	1739	139	324248	323871	398	0	hypothetical protein
AFLA_00877	AFLA_00872	5	1739	138	323658	322531	213	0	Patatin-like phospholipase family protein
AFLA_00877	AFLA_00871	6	1739	137	320117	320587	1944	0	hypothetical protein
AFLA_00877	AFLA_00870	7	1739	136	318905	319923	194	0	hypothetical protein
AFLA_00877	AFLA_00869	8	1739	135	317335	317789	1116	0	hypothetical protein
AFLA_00877	AFLA_00868	9	1739	134	315538	316891	444	0	hypothetical protein
AFLA_00877	AFLA_00867	10	1739	133	313672	314862	676	0	hypothetical protein
AFLA_00877	AFLA_00866	11	1739	132	310965	312334	1338	0	hypothetical protein
AFLA_00877	AFLA_00865	12	1739	131	310570	309992	395	1	oxidoreductase, short chain dehydrogenase/reductase family protein

Cluster:7

Backbone_ge	Gene_id	Gene_positic	Chromosome	Gene_order	5'end	3'end	Gene_distan	Domain_scor	Annotated_gene_function
AFLA_01001	AFLA_01003	-2	1739	269	705078	706213	554	1	hypothetical protein
AFLA_01001	AFLA_01002	-1	1739	268	704524	700414	744	0	nonribosomal peptide synthase, putative
AFLA_01001	AFLA_01001	0	1739	267	694790	699670	0	0	nonribosomal peptide synthase, putative
AFLA_01001	AFLA_01000	1	1739	266	687862	693859	931	0	polyketide synthase, putative
AFLA_01001	AFLA_00999	2	1739	265	686495	685466	1367	1	oxidoreductase, 2OG-Fe(II) oxygenase family, putative
AFLA_01001	AFLA_00998	3	1739	264	680721	684644	822	1	To ATP-binding cassette transporter protein YOR1, putative

Cluster:8

Backbone_ge	Gene_id	Gene_positic	Chromosome	Gene_order	5'end	3'end	Gene_distan	Domain_scor	Annotated_gene_function
AFLA_01062	AFLA_01064	-2	1739	330	860965	862908	1008	1	MFS siderophore transporter, putative
AFLA_01062	AFLA_01063	-1	1739	329	859957	855974	827	1	ABC transporter, identical
AFLA_01062	AFLA_01062	0	1739	328	848792	855147	0	0	nonribosomal siderophore peptide synthase Sid2
AFLA_01062	AFLA_01061	1	1739	327	846539	847423	1369	0	enoyl-CoA hydratase/isomerase family protein
AFLA_01062	AFLA_01060	2	1739	326	846222	844747	317	0	acetylase, putative
AFLA_01062	AFLA_01059	3	1739	325	841108	840788	3639	0	siderophore biosynthesis lipase/esterase, putative
AFLA_01062	AFLA_01058	4	1739	324	815096	838441	2347	0	nonribosomal peptide synthase, putative
AFLA_01062	AFLA_01057	5	1739	323	814602	814191	494	0	hypothetical protein
AFLA_01062	AFLA_01056	6	1739	322	812044	811769	2147	0	hypothetical protein
AFLA_01062	AFLA_01055	7	1739	321	811536	810512	233	1	O-methyltransferase family protein

Cluster:9

Backbone_ge	Gene_id	Gene_positic	Chromosome	Gene_order	5'end	3'end	Gene_distan	Domain_scor	Annotated_gene_function
AFLA_01784	AFLA_01790	-6	1866	505	1378298	1381120	3094	1	Fungal specific transcription factor domain containing protein
AFLA_01784	AFLA_01789	-5	1866	504	1373490	1375204	299	0	SET domain protein, ,putative
AFLA_01784	AFLA_01788	-4	1866	503	1373191	1372394	103	0	hypothetical protein
AFLA_01784	AFLA_01787	-3	1866	502	1370500	1372497	2640	0	GTP-binding protein, putative
AFLA_01784	AFLA_01786	-2	1866	501	1367860	1367376	1201	0	hypothetical protein
AFLA_01784	AFLA_01785	-1	1866	500	1366175	1365370	883	0	hypothetical protein
AFLA_01784	AFLA_01784	0	1866	499	1361134	1364487	0	0	NRPS-like enzyme, putative

AFLA_01784	AFLA_01783	1	1866	498	1359214	1360086	1048	0	Ribosomal protein L7Ae containing protein
AFLA_01784	AFLA_01782	2	1866	497	1352556	1355626	3588	0	Fes/CIP4 homology domain containing protein
AFLA_01784	AFLA_01781	3	1866	496	1352033	1350671	523	0	Protein kinase domain containing protein
AFLA_01784	AFLA_01780	4	1866	495	1348575	1346497	2096	0	proline-rich SH3 domain protein, ,putative
AFLA_01784	AFLA_01779	5	1866	494	1346287	1345971	210	0	New cDNA-based gene: (AO_CDS_042706, novel, updateIDs: 1439, [gene: novel_gene_185, model: novel_model_185])
AFLA_01784	AFLA_01778	6	1866	493	1343225	1345672	299	0	oligonucleotide transporter, putative
AFLA_01784	AFLA_01777	7	1866	492	1341770	1342859	366	0	D-isomer specific 2-hydroxyacid dehydrogenase, NAD binding domain containing protein
AFLA_01784	AFLA_01776	8	1866	491	1339127	1341356	414	1	FAD binding domain containing protein

Cluster:10

Backbone_ge	Gene_id	Gene_positic	Chromosome	Gene_order	5'end	3'end	Gene_distan	Domain_scor	Annotated_gene_function
AFLA_02302	AFLA_02305	-3	1918	16	42622	44123	601	1	Major Facilitator Superfamily protein
AFLA_02302	AFLA_02304	-2	1918	15	39903	42021	208	1	Fungal specific transcription factor domain containing protein
AFLA_02302	AFLA_02303	-1	1918	14	38036	39695	615	1	Cytochrome P450 family protein
AFLA_02302	AFLA_02302	0	1918	13	37421	34356	0	0	NRPS-like enzyme, putative
AFLA_02302	AFLA_02301	1	1918	12	32707	33814	542	0	hypothetical protein
AFLA_02302	AFLA_02300	2	1918	11	31125	30057	1582	0	hypothetical protein
AFLA_02302	AFLA_02299	3	1918	10	26396	27139	2918	0	hypothetical protein
AFLA_02302	AFLA_02288	4	1918	9	25237	22398	1159	1	POT family protein
AFLA_02302	AFLA_02287	5	1918	8	21929	20652	469	0	hypothetical protein
AFLA_02302	AFLA_02286	6	1918	7	20098	19673	554	0	hypothetical protein
AFLA_02302	AFLA_02285	7	1918	6	16942	17796	1877	0	Amidohydrolase family protein
AFLA_02302	AFLA_02284	8	1918	5	16614	15005	328	1	Major Facilitator Superfamily protein
AFLA_02302	AFLA_02283	9	1918	4	11750	13504	1501	0	hypothetical protein
AFLA_02302	AFLA_02282	10	1918	3	10959	7454	791	0	hypothetical protein
AFLA_02302	AFLA_02281	11	1918	2	4125	5886	1568	1	Sugar transporter family protein

Cluster:11

Backbone_ge	Gene_id	Gene_positic	Chromosome	Gene_order	5'end	3'end	Gene_distan	Domain_scor	Annotated_gene_function
AFLA_02872	AFLA_02872	0	1918	582	1595382	1592347	0	0	NRPS-like enzyme, putative
AFLA_02872	AFLA_02871	1	1918	581	1590345	1591103	1244	1	oxidoreductase, short chain dehydrogenase/reductase family protein
AFLA_02872	AFLA_02870	2	1918	580	1588291	1587194	2054	0	ubiquitin (UbiA), putative
AFLA_02872	AFLA_02869	3	1918	579	1585925	1587022	172	0	Yip1 domain containing protein
AFLA_02872	AFLA_02868	4	1918	578	1584430	1585591	334	0	hypothetical protein
AFLA_02872	AFLA_02867	5	1918	577	1582838	1578892	1592	0	thermotolerance protein, putative
AFLA_02872	AFLA_02866	6	1918	576	1578642	1574431	250	0	Elongation factor Tu GTP binding domain containing protein
AFLA_02872	AFLA_02865	7	1918	575	1573422	1574996	565	0	Rhodanese-like domain containing protein
AFLA_02872	AFLA_02864	8	1918	574	1570900	1572733	689	1	cytochrome P450 sterol C-22 desaturase, putaitve, putative

Cluster:12

Backbone_ge	Gene_id	Gene_positic	Chromosome	Gene_order	5'end	3'end	Gene_distan	Domain_scor	Annotated_gene_function
AFLA_03860	AFLA_03867	-7	2043	51	171097	173060	678	1	Major Facilitator Superfamily protein
AFLA_03860	AFLA_03866	-6	2043	50	170125	170419	682	0	New cDNA-based gene: (AO_CDS_042706, novel, updateIDs: 3547, [gene: novel_gene_397, model: novel_model_397])
AFLA_03860	AFLA_03865	-5	2043	49	168208	169443	510	0	Eukaryotic aspartyl protease family protein
AFLA_03860	AFLA_03864	-4	2043	48	162029	167698	708	1	fatty acid synthase alpha subunit, putative
AFLA_03860	AFLA_03863	-3	2043	47	161321	159465	659	1	Cytochrome P450 family protein
AFLA_03860	AFLA_03862	-2	2043	46	156808	158806	85	0	aminotransferase, class IV family protein
AFLA_03860	AFLA_03861	-1	2043	45	156723	155841	3400	0	hypothetical protein
AFLA_03860	AFLA_03860	0	2043	44	152441	143335	0	0	nonribosomal peptide synthase, putative

Cluster:13

Backbone_ge	Gene_id	Gene_positic	Chromosome	Gene_order	5'end	3'end	Gene_distan	Domain_scor	Annotated_gene_function
AFLA_04161	AFLA_04161	0	2043	345	904196	901113	0	0	NRPS-like enzyme, putative
AFLA_04161	AFLA_04160	1	2043	344	898173	900320	793	0	hypothetical protein

AFLA_04161	AFLA_04159	2	2043	343	896003	897496	677	0	hypothetical protein
AFLA_04161	AFLA_04158	3	2043	342	894565	895281	722	1	short chain dehydrogenase/oxidoreductase, putative
AFLA_04161	AFLA_04157	4	2043	341	893539	892256	1026	0	conserved hypothetical protein
AFLA_04161	AFLA_04156	5	2043	340	890819	891815	441	0	hypothetical protein
AFLA_04161	AFLA_04155	6	2043	339	888719	890082	737	0	cystathionine beta-lyase family protein
AFLA_04161	AFLA_04154	7	2043	338	884862	888121	598	1	C6 transcription factor, putative

Cluster:14

Backbone_ge	Gene_id	Gene_positic	Chromosome	Gene_order	5'end	3'end	Gene_distan	Domain_scor	Annotated_gene_function
AFLA_04549	AFLA_04556	-7	2043	740	1959838	1961722	25	1	sugar transporter family protein, putative
AFLA_04549	AFLA_04555	-6	2043	739	1959813	1959493	1529	0	hypothetical protein
AFLA_04549	AFLA_04554	-5	2043	738	1956126	1957964	456	1	cytochrome P450 monooxygenase, putative
AFLA_04549	AFLA_04553	-4	2043	737	1955281	1955670	311	0	hypothetical protein
AFLA_04549	AFLA_04552	-3	2043	736	1954480	1954970	363	0	hypothetical protein
AFLA_04549	AFLA_04551	-2	2043	735	1954117	1953325	790	0	hypothetical protein
AFLA_04549	AFLA_04550	-1	2043	734	1952535	1950597	983	1	cytochrome P450 monooxygenase, putative
AFLA_04549	AFLA_04549	0	2043	733	1949614	1948240	0	0	dimethylallyl tryptophan synthase, putative

Cluster:15

Backbone_ge	Gene_id	Gene_positic	Chromosome	Gene_order	5'end	3'end	Gene_distan	Domain_scor	Annotated_gene_function
AFLA_05387	AFLA_05391	-4	2091	595	1703485	1705440	1070	1	Major Facilitator Superfamily protein
AFLA_05387	AFLA_05390	-3	2091	594	1701195	1702415	832	0	New cDNA-based gene: (AO_CDS_042706, novel, updateIDs: 4835, [gene: novel_gene_570, model: novel_model_570])
AFLA_05387	AFLA_05389	-2	2091	593	1700363	1698957	79	0	Carboxylesterase family protein
AFLA_05387	AFLA_05388	-1	2091	592	1698878	1698657	1154	0	hypothetical protein
AFLA_05387	AFLA_05387	0	2091	591	1697503	1688441	0	1	polyketide synthase, putative
AFLA_05387	AFLA_05386	1	2091	590	1686559	1687040	1401	0	New cDNA-based gene: (AO_CDS_042706, novel, updateIDs: 4833, [gene: novel_gene_569, model: novel_model_569])
AFLA_05387	AFLA_05385	2	2091	589	1685920	1686438	121	0	hypothetical protein
AFLA_05387	AFLA_05384	3	2091	588	1684405	1681736	1515	0	conserved hypothetical protein
AFLA_05387	AFLA_05383	4	2091	587	1679345	1681385	351	1	Major Facilitator Superfamily protein
AFLA_05387	AFLA_05382	5	2091	586	1676994	1678678	667	1	Flavin-binding monooxygenase-like family protein
AFLA_05387	AFLA_05381	6	2091	585	1676347	1675102	647	0	Taurine catabolism dioxygenase TauD, TfdA family protein
AFLA_05387	AFLA_05380	7	2091	584	1674145	1674690	412	0	New cDNA-based gene: (AO_CDS_042706, novel, updateIDs: 4829, [gene: novel_gene_568, model: novel_model_568])
AFLA_05387	AFLA_05379	8	2091	583	1673157	1672510	988	0	mas-related
AFLA_05387	AFLA_05378	9	2091	582	1672498	1671444	12	0	polyketide synthase-related
AFLA_05387	AFLA_05377	10	2091	581	1671364	1670504	80	0	polyketide synthase-related
AFLA_05387	AFLA_05376	11	2091	580	1667661	1669317	1187	1	hypothetical protein

Cluster:16

Backbone_ge	Gene_id	Gene_positic	Chromosome	Gene_order	5'end	3'end	Gene_distan	Domain_scor	Annotated_gene_function
AFLA_05409	AFLA_05409	0	2091	613	1755004	1746910	0	1	polyketide synthase, putative
AFLA_05409	AFLA_05408	1	2091	612	1745043	1746458	452	0	Sodium/hydrogen exchanger family protein
AFLA_05409	AFLA_05407	2	2091	611	1743632	1744351	692	0	hypothetical protein
AFLA_05409	AFLA_05406	3	2091	610	1743152	1742205	480	0	ATP/GTP binding protein, putative
AFLA_05409	AFLA_05405	4	2091	609	1739456	1735124	2749	1	ABC-2 type transporter family protein
AFLA_05409	AFLA_05404	5	2091	608	1733389	1731120	1735	1	hypothetical protein

Cluster:17

Backbone_ge	Gene_id	Gene_positic	Chromosome	Gene_order	5'end	3'end	Gene_distan	Domain_scor	Annotated_gene_function
AFLA_05427	AFLA_05439	-12	2091	643	1827371	1829206	84	1	Fungal specific transcription factor domain containing protein
AFLA_05427	AFLA_05438	-11	2091	642	1826952	1827287	2208	0	hypothetical protein
AFLA_05427	AFLA_05437	-10	2091	641	1823709	1824744	1348	1	oxidoreductase, short chain dehydrogenase/reductase family protein
AFLA_05427	AFLA_05436	-9	2091	640	1822361	1821491	923	0	conserved hypothetical protein
AFLA_05427	AFLA_05435	-8	2091	639	1819378	1820568	643	0	actin-binding protein fragmin, putative
AFLA_05427	AFLA_05434	-7	2091	638	1817631	1818735	433	0	NmrA-like family protein

AFLA_05427	AFLA_05433	-6	2091	637	1816477	1817198	450	0	hypothetical protein
AFLA_05427	AFLA_05432	-5	2091	636	1815250	1816027	2067	0	NPP1 domain protein, putative
AFLA_05427	AFLA_05431	-4	2091	635	1813183	1810986	309	1	Fungal specific transcription factor domain containing protein
AFLA_05427	AFLA_05430	-3	2091	634	1808950	1810677	807	1	Major Facilitator Superfamily protein
AFLA_05427	AFLA_05429	-2	2091	633	1808143	1807107	224	0	oxidoreductase, aldo/keto reductase family protein
AFLA_05427	AFLA_05428	-1	2091	632	1806437	1806883	1860	0	conserved hypothetical protein
AFLA_05427	AFLA_05427	0	2091	631	1804577	1800767	0	0	NRPS-like enzyme, putative
AFLA_05427	AFLA_05426	1	2091	630	1798533	1800305	462	1	Major Facilitator Superfamily protein

Cluster:18

Backbone_ge	Gene_id	Gene_positic	Chromosome	Gene_order	5'end	3'end	Gene_distan	Domain_scoı	Annotated_gene_function
AFLA_06002	AFLA_06009	-7	2258	545	1434636	1433008	979	1	Major Facilitator Superfamily protein
AFLA_06002	AFLA_06008	-6	2258	544	1432029	1426642	1080	1	ABC transporter family protein
AFLA_06002	AFLA_06007	-5	2258	543	1423650	1425562	839	0	hypothetical protein
AFLA_06002	AFLA_06006	-4	2258	542	1422811	1421139	1392	1	Major Facilitator Superfamily protein
AFLA_06002	AFLA_06005	-3	2258	541	1417975	1419747	693	1	Amino acid permease family protein
AFLA_06002	AFLA_06004	-2	2258	540	1417282	1415945	253	0	hypothetical protein
AFLA_06002	AFLA_06003	-1	2258	539	1413349	1415692	2716	0	hypothetical protein
AFLA_06002	AFLA_06002	0	2258	538	1406717	1410633	0	0	PKS-like enzyme, putative
AFLA_06002	AFLA_06001	1	2258	537	1404376	1406005	712	0	hypothetical protein
AFLA_06002	AFLA_06000	2	2258	536	1402360	1403600	776	1	metallo-beta-lactamase superfamily protein
AFLA_06002	AFLA_05999	3	2258	535	1401933	1398469	427	1	Tryptophan halogenase family protein
AFLA_06002	AFLA_05998	4	2258	534	1396349	1397996	473	0	GMC oxidoreductase family protein
AFLA_06002	AFLA_05997	5	2258	533	1394858	1393864	1491	1	NAD dependent epimerase/dehydratase family protein
AFLA_06002	AFLA_05996	6	2258	532	1392496	1393509	355	1	hypothetical protein
AFLA_06002	AFLA_05995	7	2258	531	1389270	1390926	1570	1	oxidoreductase, FAD-binding, putative

Cluster:19

Backbone_ge	Gene_id	Gene_positic	Chromosome	Gene_order	5'end	3'end	Gene_distan	Domain_scoı	Annotated_gene_function
AFLA_06068	AFLA_06076	-8	2258	612	1604191	1608539	1500	1	hypothetical protein
AFLA_06068	AFLA_06075	-7	2258	611	1602691	1600398	1360	0	Sec1 family protein
AFLA_06068	AFLA_06074	-6	2258	610	1599038	1597858	432	0	mating-type alpha-pheromone receptor PreB
AFLA_06068	AFLA_06073	-5	2258	609	1597426	1595566	532	0	conserved hypothetical protein
AFLA_06068	AFLA_06072	-4	2258	608	1593450	1595034	495	1	oxidoreductase, oxygen dependent, FAD-dependent protein-related
AFLA_06068	AFLA_06071	-3	2258	607	1591507	1592955	418	0	hydrolase, alpha/beta fold family protein
AFLA_06068	AFLA_06070	-2	2258	606	1588821	1591089	560	0	phenylalanine ammonia-lyase family protein
AFLA_06068	AFLA_06069	-1	2258	605	1586494	1588261	170	1	Cytochrome P450 family protein
AFLA_06068	AFLA_06068	0	2258	604	1586324	1584884	0	0	dimethylallyl tryptophan synthase, putative

Cluster:20

Backbone_ge	Gene_id	Gene_positic	Chromosome	Gene_order	5'end	3'end	Gene_distan	Domain_scoı	Annotated_gene_function
AFLA_06286	AFLA_06299	-13	2258	835	2205524	2206837	244	1	hypothetical protein
AFLA_06286	AFLA_06298	-12	2258	834	2205280	2204236	691	0	enoyl-CoA hydratase/isomerase family protein
AFLA_06286	AFLA_06297	-11	2258	833	2203545	2201302	918	0	Copper amine oxidase, enzyme domain containing protein
AFLA_06286	AFLA_06296	-10	2258	832	2198253	2200384	365	1	hypothetical protein
AFLA_06286	AFLA_06295	-9	2258	831	2197888	2197476	36	0	hypothetical protein
AFLA_06286	AFLA_06294	-8	2258	830	2197440	2195885	1914	0	hypothetical protein
AFLA_06286	AFLA_06293	-7	2258	829	2193971	2192936	1047	0	glycosyl hydrolase, family 43 protein
AFLA_06286	AFLA_06292	-6	2258	828	2191254	2191889	356	0	hypothetical protein
AFLA_06286	AFLA_06291	-5	2258	827	2188732	2190898	651	1	New cDNA-based gene: (AO_CDS_042706, novel, updateIDs: 5628, [gene: novel_gene_645, model: novel_model_645])
AFLA_06286	AFLA_06290	-4	2258	826	2185955	2188081	2100	0	Acyltransferase family protein
AFLA_06286	AFLA_06289	-3	2258	825	2183855	2183270	1476	0	hypothetical protein
AFLA_06286	AFLA_06288	-2	2258	824	2181794	2180961	803	1	oxidoreductase, short chain dehydrogenase/reductase family protein
AFLA_06286	AFLA_06287	-1	2258	823	2179433	2180158	2391	0	hypothetical protein

AFLA_06286	AFLA_06286	0	2258	822	2170305	2177042	0	0	polyketide synthase, putative
AFLA_06286	AFLA_06285	1	2258	821	2169231	2167549	1074	0	Fatty acid desaturase family protein
AFLA_06286	AFLA_06284	2	2258	820	2165747	2166541	1008	0	conserved hypothetical protein
AFLA_06286	AFLA_06283	3	2258	819	2164703	2163155	1044	1	FAD binding domain containing protein
AFLA_06286	AFLA_06282	4	2258	818	2161415	2153493	1740	1	polyketide synthase, putative

Cluster:21

Backbone_ge	Gene_id	Gene_positic	Chromosome	Gene_order	5'end	3'end	Gene_distan	Domain_scoi	Annotated_gene_function
AFLA_06424	AFLA_06444	-20	2368	27	83972	81563	600	1	vacuolar ABC heavy metal transporter (Hmt1), putative
AFLA_06424	AFLA_06443	-19	2368	26	79308	80963	904	1	Major Facilitator Superfamily protein
AFLA_06424	AFLA_06442	-18	2368	25	78404	77944	1305	0	GliK-related
AFLA_06424	AFLA_06441	-17	2368	24	76639	75158	104	0	hypothetical protein
AFLA_06424	AFLA_06440	-16	2368	23	73235	75054	216	1	Cytochrome P450 family protein
AFLA_06424	AFLA_06439	-15	2368	22	73019	71207	235	1	Cytochrome P450 family protein
AFLA_06424	AFLA_06438	-14	2368	21	70972	68974	898	1	Tryptophan halogenase family protein
AFLA_06424	AFLA_06437	-13	2368	20	65641	68076	743	1	Fungal specific transcription factor domain containing protein
AFLA_06424	AFLA_06436	-12	2368	19	64898	60297	842	1	ABC transporter family protein
AFLA_06424	AFLA_06435	-11	2368	18	57672	59455	1701	1	conserved hypothetical protein
AFLA_06424	AFLA_06434	-10	2368	17	55971	55171	2098	0	haloacid dehalogenase, type II family protein
AFLA_06424	AFLA_06433	-9	2368	16	53073	51418	427	0	hypothetical protein
AFLA_06424	AFLA_06432	-8	2368	15	47758	50991	33	1	POT family protein
AFLA_06424	AFLA_06431	-7	2368	14	47408	47725	444	0	di/tri peptide transporter 2-related
AFLA_06424	AFLA_06430	-6	2368	13	46964	45453	267	0	FAD dependent oxidoreductase family protein
AFLA_06424	AFLA_06429	-5	2368	12	45186	44443	427	0	O-methyltransferase family protein
AFLA_06424	AFLA_06428	-4	2368	11	42851	44016	537	1	hypothetical protein
AFLA_06424	AFLA_06427	-3	2368	10	41154	42314	644	1	hypothetical protein
AFLA_06424	AFLA_06426	-2	2368	9	40510	38582	1480	0	MFS peptide transporter, putaitve, putative
AFLA_06424	AFLA_06425	-1	2368	8	36311	37102	920	1	FAD binding domain containing protein
AFLA_06424	AFLA_06424	0	2368	7	35391	25905	0	0	nonribosomal peptide synthase, putative

Cluster:22

Backbone_ge	Gene_id	Gene_positic	Chromosome	Gene_order	5'end	3'end	Gene_distan	Domain_scoi	Annotated_gene_function
AFLA_06456	AFLA_06461	-5	2368	44	116491	115109	798	1	oxidoreductase, short chain dehydrogenase/reductase family protein
AFLA_06456	AFLA_06460	-4	2368	43	112870	114311	707	0	hypothetical protein
AFLA_06456	AFLA_06459	-3	2368	42	112163	111281	263	1	O-methyltransferase family protein
AFLA_06456	AFLA_06458	-2	2368	41	109800	111018	248	0	Oxidoreductase family, NAD-binding Rossmann fold containing protein
AFLA_06456	AFLA_06457	-1	2368	40	109552	107693	804	0	permease, cytosine/purines, uracil, thiamine, allantoin family protein
AFLA_06456	AFLA_06456	0	2368	39	106889	101963	0	0	nonribosomal peptide synthase, putative
AFLA_06456	AFLA_06455	1	2368	38	100260	101660	303	1	Renal dipeptidase family protein
AFLA_06456	AFLA_06454	2	2368	37	100034	98411	226	1	Cytochrome P450 family protein
AFLA_06456	AFLA_06453	3	2368	36	97373	98280	131	1	Glutathione S-transferase, C-terminal domain containing protein
AFLA_06456	AFLA_06452	4	2368	35	95877	97232	141	1	O-methyltransferase GliM-like, putative
AFLA_06456	AFLA_06451	5	2368	34	95613	94556	264	1	GliT-related
AFLA_06456	AFLA_06450	6	2368	33	93592	92595	964	1	pyridine nucleotide-disulphide oxidoreductase, class II-related
AFLA_06456	AFLA_06449	7	2368	32	91098	92168	427	1	7alpha-cephem-methoxylase P8 chain, putative
AFLA_06456	AFLA_06448	8	2368	31	90822	89773	276	1	GliT-related
AFLA_06456	AFLA_06447	9	2368	30	89655	87729	118	1	Cytochrome P450 family protein
AFLA_06456	AFLA_06446	10	2368	29	86181	87534	195	0	hypothetical protein
AFLA_06456	AFLA_06445	11	2368	28	84795	86027	154	1	aminotransferase, classes I and II family protein
AFLA_06456	AFLA_06444	12	2368	27	83972	81563	823	1	vacuolar ABC heavy metal transporter (Hmt1), putative
AFLA_06456	AFLA_06443	13	2368	26	79308	80963	600	1	Major Facilitator Superfamily protein
AFLA_06456	AFLA_06442	14	2368	25	78404	77944	904	0	GliK-related
AFLA_06456	AFLA_06441	15	2368	24	76639	75158	1305	0	hypothetical protein
AFLA_06456	AFLA_06440	16	2368	23	73235	75054	104	1	Cytochrome P450 family protein

AFLA_06456	AFLA_06439	17	2368	22	73019	71207	216	1	Cytochrome P450 family protein
AFLA_06456	AFLA_06438	18	2368	21	70972	68974	235	1	Tryptophan halogenase family protein
AFLA_06456	AFLA_06437	19	2368	20	65641	68076	898	1	Fungal specific transcription factor domain containing protein
AFLA_06456	AFLA_06436	20	2368	19	64898	60297	743	1	ABC transporter family protein

Cluster:23

Backbone_ge	Gene_id	Gene_positic	Chromosome	Gene_order	5'end	3'end	Gene_distan	Domain_scoi	Annotated_gene_function
AFLA_06684	AFLA_06698	-14	2368	281	742432	749952	979	1	polyketide synthase, putative
AFLA_06684	AFLA_06697	-13	2368	280	741453	739298	487	0	conserved hypothetical protein
AFLA_06684	AFLA_06696	-12	2368	279	738385	738811	141	0	hypothetical protein
AFLA_06684	AFLA_06695	-11	2368	278	736840	738244	528	0	FAD binding domain containing protein
AFLA_06684	AFLA_06694	-10	2368	277	734919	736312	333	1	O-methyltransferase family protein
AFLA_06684	AFLA_06693	-9	2368	276	734586	732934	212	1	Cytochrome P450 family protein
AFLA_06684	AFLA_06692	-8	2368	275	731458	732722	237	0	ToxD-like zinc binding oxidoreductase, putative
AFLA_06684	AFLA_06691	-7	2368	274	731221	730081	715	0	hypothetical protein
AFLA_06684	AFLA_06690	-6	2368	273	726830	729366	2276	1	Fungal specific transcription factor domain containing protein
AFLA_06684	AFLA_06689	-5	2368	272	724554	722643	599	1	Cytochrome P450 family protein
AFLA_06684	AFLA_06688	-4	2368	271	720534	722044	633	1	Major Facilitator Superfamily protein
AFLA_06684	AFLA_06687	-3	2368	270	719901	719479	75	1	hypothetical protein
AFLA_06684	AFLA_06686	-2	2368	269	719404	717626	330	0	conserved hypothetical protein
AFLA_06684	AFLA_06685	-1	2368	268	716818	717296	285	0	hypothetical protein
AFLA_06684	AFLA_06684	0	2368	267	716533	704693	0	1	hybrid NRPS/PKS enzyme, putative
AFLA_06684	AFLA_06683	1	2368	266	703972	701610	721	1	Fungal specific transcription factor domain containing protein
AFLA_06684	AFLA_06682	2	2368	265	698602	699772	1838	1	oxidoreductase, zinc-binding dehydrogenase family protein
AFLA_06684	AFLA_06681	3	2368	264	696628	697948	654	0	hypothetical protein
AFLA_06684	AFLA_06680	4	2368	263	695070	694459	1558	0	hypothetical protein
AFLA_06684	AFLA_06679	5	2368	262	690975	693451	1008	0	hypothetical protein
AFLA_06684	AFLA_06678	6	2368	261	688382	690085	890	0	Polyprenyl synthetase family protein
AFLA_06684	AFLA_06677	7	2368	260	688074	686534	308	1	FAD dependent oxidoreductase, putative
AFLA_06684	AFLA_06676	8	2368	259	682176	685132	1402	0	unknown-related
AFLA_06684	AFLA_06675	9	2368	258	678960	681518	658	0	Glycosyl hydrolase family 3 C terminal domain containing protein
AFLA_06684	AFLA_06674	10	2368	257	676143	677540	1420	1	Major Facilitator Superfamily protein
AFLA_06684	AFLA_06673	11	2368	256	673317	674393	1750	1	oxidoreductase, zinc-binding dehydrogenase family protein
AFLA_06684	AFLA_06672	12	2368	255	655460	671802	1515	0	nonribosomal peptide synthase, putative
AFLA_06684	AFLA_06671	13	2368	254	655109	653979	351	1	hypothetical protein
AFLA_06684	AFLA_06670	14	2368	253	653630	652134	349	1	Cytochrome P450 family protein

Cluster:24

Backbone_ge	Gene_id	Gene_positic	Chromosome	Gene_order	5'end	3'end	Gene_distan	Domain_scoi	Annotated_gene_function
AFLA_06933	AFLA_06941	-8	2368	524	1399261	1396108	580	1	RNase L inhibitor of the ABC superfamily, putative
AFLA_06933	AFLA_06940	-7	2368	523	1392947	1395528	290	0	FF domain containing protein
AFLA_06933	AFLA_06939	-6	2368	522	1392657	1390724	691	0	hypothetical protein
AFLA_06933	AFLA_06938	-5	2368	521	1390033	1388908	831	0	Mpv17 / PMP22 family protein
AFLA_06933	AFLA_06937	-4	2368	520	1388077	1386692	189	0	phosphoglycerate kinase PgkA, putative
AFLA_06933	AFLA_06936	-3	2368	519	1385454	1386503	796	1	26 proteasome complex subunit Sem1, putative
AFLA_06933	AFLA_06935	-2	2368	518	1384658	1382210	980	0	conserved hypothetical protein
AFLA_06933	AFLA_06934	-1	2368	517	1381230	1379343	569	1	MSF drug transporter, putative
AFLA_06933	AFLA_06933	0	2368	516	1362826	1378774	0	0	nonribosomal peptide synthase Pes1

Cluster:25

Backbone_ge	Gene_id	Gene_positic	Chromosome	Gene_order	5'end	3'end	Gene_distan	Domain_scoi	Annotated_gene_function
AFLA_07092	AFLA_07098	-6	2368	681	1864430	1861877	932	1	C6 transcription factor, putative
AFLA_07092	AFLA_07097	-5	2368	680	1860945	1858934	269	1	Fungal specific transcription factor domain containing protein
AFLA_07092	AFLA_07096	-4	2368	679	1855567	1858665	1634	0	AT hook motif family protein

AFLA_07092	AFLA_07095	-3	2368	678	1852565	1853933	1896	0	malate permease, ,putative
AFLA_07092	AFLA_07094	-2	2368	677	1850669	1848985	322	0	extracellular rhamnogalacturonase, putative
AFLA_07092	AFLA_07093	-1	2368	676	1848663	1846933	1152	1	membrane transporter; 11 predicted transmembrane helices, putative
AFLA_07092	AFLA_07092	0	2368	675	1842755	1845781	0	0	NRPS-like enzyme, putative
AFLA_07092	AFLA_07091	1	2368	674	1841501	1840100	1254	1	aminotransferase, classes I and II family protein
AFLA_07092	AFLA_07090	2	2368	673	1838063	1838698	1402	0	hypothetical protein
AFLA_07092	AFLA_07089	3	2368	672	1836192	1836995	1068	0	hypothetical protein
AFLA_07092	AFLA_07088	4	2368	671	1833085	1834369	1823	1	Acyl-coenzyme A:6-aminopenicillanic-acid-acyltransferase precursor, putative
AFLA_07092	AFLA_07087	5	2368	670	1831356	1832351	734	1	New cDNA-based gene: (AO_CDS_042706, novel, updateIDs: 6373, [gene: novel_gene_717, model: novel_model_717])
AFLA_07092	AFLA_07086	6	2368	669	1830330	1819006	1026	0	nonribosomal peptide synthase, putative
AFLA_07092	AFLA_07085	7	2368	668	1818468	1817090	538	0	Phosphatidylserine decarboxylase, ,putative
AFLA_07092	AFLA_07084	8	2368	667	1815175	1816971	119	1	Major Facilitator Superfamily protein
AFLA_07092	AFLA_07083	9	2368	666	1809066	1814555	620	0	hypothetical protein
AFLA_07092	AFLA_07082	10	2368	665	1804776	1806633	2433	1	3-hydroxy-3-methylglutaryl-coenzyme A lyase/3-methylglutaconyl-coenzyme A hydratase, putative
AFLA_07092	AFLA_07081	11	2368	664	1801567	1799384	3209	0	Carboxylesterase family protein
AFLA_07092	AFLA_07080	12	2368	663	1797196	1798842	542	0	hypothetical protein
AFLA_07092	AFLA_07079	13	2368	662	1795038	1794436	2158	0	New cDNA-based gene: (AO_CDS_042706, novel, updateIDs: 6368, [gene: novel_gene_716, model: novel_model_716])
AFLA_07092	AFLA_07078	14	2368	661	1793654	1791721	782	0	Amidase family protein
AFLA_07092	AFLA_07077	15	2368	660	1788584	1786982	3137	0	hypothetical protein
AFLA_07092	AFLA_07076	16	2368	659	1784323	1786494	488	1	Major Facilitator Superfamily protein
AFLA_07092	AFLA_07075	17	2368	658	1781368	1781614	2709	0	New cDNA-based gene: (AO_CDS_042706, novel, updateIDs: 6365, [gene: novel_gene_715, model: novel_model_715])
AFLA_07092	AFLA_07074	18	2368	657	1780336	1779468	1032	0	hypothetical protein
AFLA_07092	AFLA_07073	19	2368	656	1774151	1777064	2404	1	hypothetical protein

Cluster:26

Backbone_ge	Gene_id	Gene_positic	Chromosome	Gene_order	5'end	3'end	Gene_distan	Domain_scor	Annotated_gene_function
AFLA_07940	AFLA_07951	-11	2504	734	1932862	1930791	109	1	Major Facilitator Superfamily protein
AFLA_07940	AFLA_07950	-10	2504	733	1929684	1930682	885	0	xylosidase, putative
AFLA_07940	AFLA_07949	-9	2504	732	1928248	1928799	470	0	hypothetical protein
AFLA_07940	AFLA_07948	-8	2504	731	1925515	1927778	526	0	prolyl oligopeptidase family protein
AFLA_07940	AFLA_07947	-7	2504	730	1918357	1924989	611	0	pre-mRNA splicing helicase, putative
AFLA_07940	AFLA_07946	-6	2504	729	1917746	1914711	179	0	MutS domain III family protein
AFLA_07940	AFLA_07945	-5	2504	728	1913795	1914532	264	0	Ureidoglycolate hydrolase family protein
AFLA_07940	AFLA_07944	-4	2504	727	1912113	1913531	1038	1	Major Facilitator Superfamily protein
AFLA_07940	AFLA_07943	-3	2504	726	1911075	1910291	1926	0	glutamyl-tRNA(Gln) amidotransferase subunit A, putative
AFLA_07940	AFLA_07942	-2	2504	725	1908365	1907742	218	0	hypothetical protein
AFLA_07940	AFLA_07941	-1	2504	724	1905565	1907524	508	1	Major Facilitator Superfamily protein
AFLA_07940	AFLA_07940	0	2504	723	1901221	1905057	0	1	NRPS-like enzyme, putative
AFLA_07940	AFLA_07939	1	2504	722	1898598	1899094	2127	0	hypothetical protein
AFLA_07940	AFLA_07938	2	2504	721	1894656	1897651	947	0	NRPS-like enzyme, putative
AFLA_07940	AFLA_07937	3	2504	720	1894296	1894484	172	0	hypothetical protein
AFLA_07940	AFLA_07936	4	2504	719	1892994	1892237	1302	0	PKS-like enzyme, putative
AFLA_07940	AFLA_07935	5	2504	718	1890807	1890073	1430	0	hypothetical protein
AFLA_07940	AFLA_07934	6	2504	717	1886382	1887372	2701	0	Dienelactone hydrolase family protein
AFLA_07940	AFLA_07933	7	2504	716	1885259	1883652	1123	0	Transmembrane amino acid transporter protein
AFLA_07940	AFLA_07932	8	2504	715	1882763	1880467	889	1	Fungal specific transcription factor domain containing protein

Cluster:27

Backbone_ge	Gene_id	Gene_positic	Chromosome	Gene_order	5'end	3'end	Gene_distan	Domain_scor	Annotated_gene_function
AFLA_08215	AFLA_08228	-13	2504	1011	2680129	2679227	189	1	Glutathione S-transferase, N-terminal domain containing protein
AFLA_08215	AFLA_08227	-12	2504	1010	2677008	2679038	554	0	UDP-glucose/GDP-mannose dehydrogenase family, UDP binding domain containing protein
AFLA_08215	AFLA_08226	-11	2504	1009	2676454	2675009	698	0	glycosyl transferase, group 1 family protein
AFLA_08215	AFLA_08225	-10	2504	1008	2674311	2672301	1400	0	GMC oxidoreductase family protein
AFLA_08215	AFLA_08224	-9	2504	1007	2670901	2669988	1635	0	hypothetical protein

AFLA_08215	AFLA_08223	-8	2504	1006	2668353	2666670	119	1	Major Facilitator Superfamily protein
AFLA_08215	AFLA_08222	-7	2504	1005	2664797	2666551	283	0	oxidoreductase-related
AFLA_08215	AFLA_08221	-6	2504	1004	2664514	2663441	776	0	hypothetical protein
AFLA_08215	AFLA_08220	-5	2504	1003	2662665	2660638	531	0	ATPase, AAA family protein
AFLA_08215	AFLA_08219	-4	2504	1002	2660107	2659280	856	0	hypothetical protein
AFLA_08215	AFLA_08218	-3	2504	1001	2658424	2658155	50	0	hypothetical protein
AFLA_08215	AFLA_08217	-2	2504	1000	2658105	2656834	1460	1	Major Facilitator Superfamily protein
AFLA_08215	AFLA_08216	-1	2504	999	2655374	2653373	536	1	Sugar transporter family protein
AFLA_08215	AFLA_08215	0	2504	998	2646293	2652837	0	0	polyketide synthase, putative

Cluster:28

Backbone_ge	Gene_id	Gene_positic	Chromosome	Gene_order	5'end	3'end	Gene_distan	Domain_scor	Annotated_gene_function
AFLA_08248	AFLA_08248	0	2504	1031	2730816	2732067	0	1	NRPS-like enzyme, putative
AFLA_08248	AFLA_08247	1	2504	1030	2730410	2728379	406	0	AMP-binding enzyme family protein
AFLA_08248	AFLA_08246	2	2504	1029	2726731	2727646	733	0	Phosphatidylserine decarboxylase-related
AFLA_08248	AFLA_08245	3	2504	1028	2726036	2724533	695	1	Major Facilitator Superfamily protein
AFLA_08248	AFLA_08244	4	2504	1027	2723068	2723992	541	0	hypothetical protein
AFLA_08248	AFLA_08243	5	2504	1026	2721452	2722705	363	1	hypothetical protein
AFLA_08248	AFLA_08242	6	2504	1025	2718976	2719365	2087	0	New cDNA-based gene: (AO_CDS_042706, novel, updateIDs: 7291, [gene: novel_gene_812, model: novel_model_812])
AFLA_08248	AFLA_08241	7	2504	1024	2718542	2717312	434	0	hypothetical protein
AFLA_08248	AFLA_08240	8	2504	1023	2716786	2712072	526	1	ABC transporter family protein

Cluster:29

Backbone_ge	Gene_id	Gene_positic	Chromosome	Gene_order	5'end	3'end	Gene_distan	Domain_scor	Annotated_gene_function
AFLA_08325	AFLA_08333	-8	2504	1116	2962748	2959979	367	1	Fungal specific transcription factor domain containing protein
AFLA_08325	AFLA_08332	-7	2504	1115	2957830	2959612	1205	1	Sugar transporter family protein
AFLA_08325	AFLA_08331	-6	2504	1114	2956625	2955630	1556	0	Amidohydrolase family protein
AFLA_08325	AFLA_08330	-5	2504	1113	2951101	2954074	2515	0	Glycosyl hydrolases family 31 protein
AFLA_08325	AFLA_08329	-4	2504	1112	2948586	2947507	1194	0	conserved hypothetical protein
AFLA_08325	AFLA_08328	-3	2504	1111	2946313	2945832	1718	0	hypothetical protein
AFLA_08325	AFLA_08327	-2	2504	1110	2944114	2942091	935	1	Amino acid permease family protein
AFLA_08325	AFLA_08326	-1	2504	1109	2941156	2939989	1858	0	hypothetical protein
AFLA_08325	AFLA_08325	0	2504	1108	2938131	2936772	0	0	dimethylallyl tryptophan synthase, putative
AFLA_08325	AFLA_08324	1	2504	1107	2934533	2935740	1032	1	xylitol dehydrogenase LadA/XdhB

Cluster:30

Backbone_ge	Gene_id	Gene_positic	Chromosome	Gene_order	5'end	3'end	Gene_distan	Domain_scor	Annotated_gene_function
AFLA_08408	AFLA_08421	-13	2504	1204	3200982	3200087	1137	1	hypothetical protein
AFLA_08408	AFLA_08420	-12	2504	1203	3198950	3197412	810	0	hypothetical protein
AFLA_08408	AFLA_08419	-11	2504	1202	3196602	3194506	152	0	hypothetical protein
AFLA_08408	AFLA_08418	-10	2504	1201	3194354	3193799	442	0	New cDNA-based gene: (AO_CDS_042706, novel, updateIDs: 7429, [gene: novel_gene_834, model: novel_model_834])
AFLA_08408	AFLA_08417	-9	2504	1200	3193357	3191475	885	0	Multicopper oxidase family protein
AFLA_08408	AFLA_08416	-8	2504	1199	3190590	3189654	461	0	conserved hypothetical protein
AFLA_08408	AFLA_08415	-7	2504	1198	3188069	3189193	863	0	peroxisome biogenesis factor, putative
AFLA_08408	AFLA_08414	-6	2504	1197	3187206	3185676	592	1	2-oxoglutarate dehydrogenase, E2 component, dihydrolipoamide succinyltransferase family protein
AFLA_08408	AFLA_08413	-5	2504	1196	3185084	3183580	60	1	2-oxoglutarate dehydrogenase E1 component, mitochondrial precursor-related
AFLA_08408	AFLA_08412	-4	2504	1195	3183520	3183068	53	1	2-oxoglutarate dehydrogenase E1 component, mitochondrial precursor-related
AFLA_08408	AFLA_08411	-3	2504	1194	3183015	3182578	141	1	2-oxoglutarate dehydrogenase E1 component, mitochondrial precursor, putative
AFLA_08408	AFLA_08410	-2	2504	1193	3182437	3181824	90	1	2-oxoglutarate dehydrogenase E1 component, mitochondrial precursor-related
AFLA_08408	AFLA_08409	-1	2504	1192	3179694	3181734	696	1	Fungal specific transcription factor domain containing protein
AFLA_08408	AFLA_08408	0	2504	1191	3177863	3178998	0	0	dimethylallyl tryptophan synthase, putative

Cluster:31

Backbone_ge	Gene_id	Gene_positic	Chromosome	Gene_order	5'end	3'end	Gene_distan	Domain_scor	Annotated_gene_function

AFLA_09020	AFLA_09021	-1	2541	115	309444	307780	344	1	Amino acid permease family protein
AFLA_09020	AFLA_09020	0	2541	114	302181	307436	0	0	nonribosomal peptide synthase, putative
AFLA_09020	AFLA_09019	1	2541	113	300318	298721	1863	0	dimethylallyl tryptophan synthase, putative
AFLA_09020	AFLA_09018	2	2541	112	298048	297158	673	0	hydrolase, alpha/beta fold family protein
AFLA_09020	AFLA_09017	3	2541	111	294013	295717	1441	1	Sugar transporter family protein

Cluster:32

Backbone_ge	Gene_id	Gene_positic	Chromosome	Gene_order	5'end	3'end	Gene_distan	Domain_scor	Annotated_gene_function
AFLA_09677	AFLA_09677	0	2541	771	2022113	2025146	0	0	polyketide synthase, putative
AFLA_09677	AFLA_09676	1	2541	770	2019179	2020813	1300	1	Major Facilitator Superfamily protein
AFLA_09677	AFLA_09675	2	2541	769	2018354	2017183	825	1	Cytochrome P450 family protein
AFLA_09677	AFLA_09674	3	2541	768	2016237	2014725	946	1	FAD binding domain containing protein

Cluster:33

Backbone_ge	Gene_id	Gene_positic	Chromosome	Gene_order	5'end	3'end	Gene_distan	Domain_scor	Annotated_gene_function
AFLA_10170	AFLA_10174	-4	2634	467	1211563	1209968	357	1	major facilitator superfamily protein
AFLA_10170	AFLA_10173	-3	2634	466	1207868	1209611	694	1	Cytochrome P450 family protein
AFLA_10170	AFLA_10172	-2	2634	465	1207174	1205421	1529	1	Cytochrome P450 family protein
AFLA_10170	AFLA_10171	-1	2634	464	1202775	1203892	685	0	NmrA-like family protein
AFLA_10170	AFLA_10170	0	2634	463	1202090	1198962	0	0	NRPS-like enzyme, putative
AFLA_10170	AFLA_10169	1	2634	462	1197753	1195973	1209	1	oxidoreductase, zinc-binding dehydrogenase family protein

Cluster:34

Backbone_ge	Gene_id	Gene_positic	Chromosome	Gene_order	5'end	3'end	Gene_distan	Domain_scor	Annotated_gene_function
AFLA_10421	AFLA_10425	-4	2689	7	24095	26449	354	1	conserved hypothetical protein
AFLA_10421	AFLA_10424	-3	2689	6	22682	23741	601	0	PKS-like enzyme, putative
AFLA_10421	AFLA_10423	-2	2689	5	22081	20244	240	1	Major Facilitator Superfamily protein
AFLA_10421	AFLA_10422	-1	2689	4	17820	20004	1125	1	Fungal specific transcription factor domain containing protein
AFLA_10421	AFLA_10421	0	2689	3	16695	14187	0	0	PKS-like enzyme, putative

Cluster:35

Backbone_ge	Gene_id	Gene_positic	Chromosome	Gene_order	5'end	3'end	Gene_distan	Domain_scor	Annotated_gene_function
AFLA_10519	AFLA_10530	-11	2689	112	297517	298524	660	1	metallo-beta-lactamase superfamily protein
AFLA_10519	AFLA_10529	-10	2689	111	296857	296367	1380	0	hypothetical protein
AFLA_10519	AFLA_10528	-9	2689	110	293919	294987	1692	0	hypothetical protein
AFLA_10519	AFLA_10527	-8	2689	109	292227	291730	888	0	hypothetical protein
AFLA_10519	AFLA_10526	-7	2689	108	290842	290185	407	0	hypothetical protein
AFLA_10519	AFLA_10525	-6	2689	107	289778	289009	1229	0	hypothetical protein
AFLA_10519	AFLA_10524	-5	2689	106	287341	287780	2368	0	hypothetical protein
AFLA_10519	AFLA_10523	-4	2689	105	284973	283497	447	0	hypothetical protein
AFLA_10519	AFLA_10522	-3	2689	104	283050	282265	78	1	oxidoreductase, short chain dehydrogenase/reductase family protein
AFLA_10519	AFLA_10521	-2	2689	103	282187	281052	2666	0	enoyl-CoA hydratase/isomerase family protein
AFLA_10519	AFLA_10520	-1	2689	102	278386	276383	598	0	GPI anchored glucanase, putative
AFLA_10519	AFLA_10519	0	2689	101	272459	275785	0	0	NRPS-like enzyme, putative
AFLA_10519	AFLA_10518	1	2689	100	270065	269796	2394	0	hypothetical protein
AFLA_10519	AFLA_10517	2	2689	99	269020	267559	776	1	O-methyltransferase family protein
AFLA_10519	AFLA_10516	3	2689	98	264930	265400	2159	0	hypothetical protein
AFLA_10519	AFLA_10515	4	2689	97	263456	264876	54	0	Carboxylesterase family protein
AFLA_10519	AFLA_10514	5	2689	96	262532	262940	516	0	hypothetical protein
AFLA_10519	AFLA_10513	6	2689	95	260881	261653	879	0	hypothetical protein
AFLA_10519	AFLA_10512	7	2689	94	259615	260166	715	1	hypothetical protein
AFLA_10519	AFLA_10511	8	2689	93	259414	258906	201	0	hypothetical protein
AFLA_10519	AFLA_10510	9	2689	92	256537	258553	353	0	conserved hypothetical protein
AFLA_10519	AFLA_10509	10	2689	91	256083	254102	454	1	New cDNA-based gene: (AO_CDS_042706, novel, updateIDs: 9638, [gene: novel_gene_1014, model: novel_model_1014

Cluster:36

Backbone_ge	Gene_id	Gene_positic	Chromosome	Gene_order	5'end	3'end	Gene_distan	Domain_scoı	Annotated_gene_function
AFLA_10545	AFLA_10545	0	2689	127	336825	345056	0	1	polyketide synthase, putative
AFLA_10545	AFLA_10544	1	2689	126	335722	334376	1103	1	Cytochrome P450 family protein

Cluster:37

Backbone_ge	Gene_id	Gene_positic	Chromosome	Gene_order	5'end	3'end	Gene_distan	Domain_scoı	Annotated_gene_function
AFLA_10855	AFLA_10861	-6	2689	443	1165381	1166754	3305	1	Cytochrome P450 family protein
AFLA_10855	AFLA_10860	-5	2689	442	1162076	1161182	112	1	acetyltransferase, GNAT family protein
AFLA_10855	AFLA_10859	-4	2689	441	1160173	1161070	2919	0	hypothetical protein
AFLA_10855	AFLA_10858	-3	2689	440	1157254	1155495	612	1	hypothetical protein
AFLA_10855	AFLA_10857	-2	2689	439	1154883	1153809	584	0	hypothetical protein
AFLA_10855	AFLA_10856	-1	2689	438	1151758	1153225	1551	1	O-methyltransferase family protein
AFLA_10855	AFLA_10855	0	2689	437	1150207	1144762	0	0	polyketide synthase, putative

Cluster:38

Backbone_ge	Gene_id	Gene_positic	Chromosome	Gene_order	5'end	3'end	Gene_distan	Domain_scoı	Annotated_gene_function
AFLA_10943	AFLA_10943	0	2689	525	1416093	1401753	0	0	nonribosomal siderophore peptide synthase SidC
AFLA_10943	AFLA_10942	1	2689	524	1398897	1401293	460	0	predicted protein
AFLA_10943	AFLA_10941	2	2689	523	1395653	1397964	933	0	UV-endonuclease uvde family protein
AFLA_10943	AFLA_10940	3	2689	522	1394651	1393611	1002	0	hypothetical protein
AFLA_10943	AFLA_10939	4	2689	521	1390969	1391828	1783	0	conserved hypothetical protein
AFLA_10943	AFLA_10938	5	2689	520	1388586	1389948	1021	1	oxidoreductase, zinc-binding dehydrogenase family protein

Cluster:39

Backbone_ge	Gene_id	Gene_positic	Chromosome	Gene_order	5'end	3'end	Gene_distan	Domain_scoı	Annotated_gene_function
AFLA_11284	AFLA_11289	-5	2689	871	2323499	2325296	520	1	Major Facilitator Superfamily protein
AFLA_11284	AFLA_11288	-4	2689	870	2322979	2320865	135	1	FAD binding domain containing protein
AFLA_11284	AFLA_11287	-3	2689	869	2318738	2320730	191	1	FAD dependent oxidoreductase family protein
AFLA_11284	AFLA_11286	-2	2689	868	2318547	2317464	752	0	hypothetical protein
AFLA_11284	AFLA_11285	-1	2689	867	2316712	2315306	507	1	O-methyltransferase family protein
AFLA_11284	AFLA_11284	0	2689	866	2307795	2314799	0	0	polyketide synthase, putative
AFLA_11284	AFLA_11283	1	2689	865	2304569	2306866	929	1	Fungal specific transcription factor domain containing protein
AFLA_11284	AFLA_11282	2	2689	864	2300919	2299817	3650	1	NAD dependent epimerase/dehydratase family protein

Cluster:40

Backbone_ge	Gene_id	Gene_positic	Chromosome	Gene_order	5'end	3'end	Gene_distan	Domain_scoı	Annotated_gene_function
AFLA_11482	AFLA_11482	0	2802	63	153643	159213	0	1	polyketide synthase, putative
AFLA_11482	AFLA_11481	1	2802	62	151713	149890	1930	1	Cytochrome P450 family protein

Cluster:41

Backbone_ge	Gene_id	Gene_positic	Chromosome	Gene_order	5'end	3'end	Gene_distan	Domain_scoı	Annotated_gene_function
AFLA_11622	AFLA_11633	-11	2842	61	148002	149287	596	1	O-methyltransferase family protein
AFLA_11622	AFLA_11632	-10	2842	60	146401	147406	2482	0	hypothetical protein
AFLA_11622	AFLA_11631	-9	2842	59	142284	143919	124	0	conserved hypothetical protein
AFLA_11622	AFLA_11630	-8	2842	58	142160	141351	433	1	oxidoreductase, short chain dehydrogenase/reductase family protein
AFLA_11622	AFLA_11629	-7	2842	57	140918	140597	474	0	hypothetical protein
AFLA_11622	AFLA_11628	-6	2842	56	140123	138135	319	0	Dehydratase family protein
AFLA_11622	AFLA_11627	-5	2842	55	136575	137816	775	0	hypothetical protein
AFLA_11622	AFLA_11626	-4	2842	54	134791	135800	424	0	N-acetyltransferase family protein
AFLA_11622	AFLA_11625	-3	2842	53	133302	134367	775	0	conserved hypothetical protein
AFLA_11622	AFLA_11624	-2	2842	52	132527	131580	1094	0	conserved hypothetical protein
AFLA_11622	AFLA_11623	-1	2842	51	129644	130486	1335	0	hypothetical protein

AFLA_11622	AFLA_11622	0	2842	50	121844	128309	0	0	polyketide synthase, putative
AFLA_11622	AFLA_11621	1	2842	49	119733	121105	739	1	O-methyltransferase family protein
AFLA_11622	AFLA_11620	2	2842	48	118012	118518	1215	0	hypothetical protein
AFLA_11622	AFLA_11619	3	2842	47	116560	110436	1452	0	hypothetical protein
AFLA_11622	AFLA_11618	4	2842	46	108807	109508	928	0	hypothetical protein
AFLA_11622	AFLA_11617	5	2842	45	108373	107985	434	0	hypothetical protein
AFLA_11622	AFLA_11616	6	2842	44	105722	104504	2263	1	oxidoreductase, zinc-binding dehydrogenase family protein
AFLA_11622	AFLA_11615	7	2842	43	103586	104193	311	0	hypothetical protein
AFLA_11622	AFLA_11614	8	2842	42	102202	100917	1384	0	hypothetical protein
AFLA_11622	AFLA_11613	9	2842	41	99530	100198	719	0	hypothetical protein
AFLA_11622	AFLA_11612	10	2842	40	97549	94432	1981	0	conserved hypothetical protein
AFLA_11622	AFLA_11611	11	2842	39	92279	94315	117	1	Fungal specific transcription factor domain containing protein
AFLA_11622	AFLA_11610	12	2842	38	91917	90391	362	1	Major Facilitator Superfamily protein
AFLA_11622	AFLA_11609	13	2842	37	88277	90102	289	1	Sugar transporter family protein

Cluster:42

Backbone_ge	Gene_id	Gene_positic	Chromosome	Gene_order	5'end	3'end	Gene_distan	Domain_scor	Annotated_gene_function
AFLA_11660	AFLA_11660	0	2842	88	206389	207845	0	0	dimethylallyl tryptophan synthase, putative
AFLA_11660	AFLA_11659	1	2842	87	205225	204489	1164	0	CRAL/TRIO domain containing protein
AFLA_11660	AFLA_11658	2	2842	86	203716	202349	773	1	3-beta hydroxysteroid dehydrogenase/isomerase family protein
AFLA_11660	AFLA_11657	3	2842	85	201402	201920	429	0	DUF636 domain protein, putative
AFLA_11660	AFLA_11656	4	2842	84	200998	200009	404	1	2-hydroxyisoflavone reductase, putative
AFLA_11660	AFLA_11655	5	2842	83	199601	199031	408	0	Phage lysozyme family protein
AFLA_11660	AFLA_11654	6	2842	82	197296	198006	1025	0	hypothetical protein
AFLA_11660	AFLA_11653	7	2842	81	194775	193219	2521	1	Cytochrome P450 family protein
AFLA_11660	AFLA_11652	8	2842	80	191867	193037	182	1	hypothetical protein
AFLA_11660	AFLA_11651	9	2842	79	191562	190351	305	0	hypothetical protein
AFLA_11660	AFLA_11650	10	2842	78	188716	190220	131	1	oxidoreductase, short chain dehydrogenase/reductase family protein
AFLA_11660	AFLA_11649	11	2842	77	187561	188037	679	0	hypothetical protein
AFLA_11660	AFLA_11648	12	2842	76	187194	186108	367	0	NAD dependent epimerase/dehydratase family protein
AFLA_11660	AFLA_11647	13	2842	75	185066	184224	1042	1	metallo-beta-lactamase superfamily protein
AFLA_11660	AFLA_11646	14	2842	74	182370	183151	1073	0	oxidoreductase-related
AFLA_11660	AFLA_11645	15	2842	73	180441	182039	331	1	FAD binding domain containing protein

Cluster:43

Backbone_ge	Gene_id	Gene_positic	Chromosome	Gene_order	5'end	3'end	Gene_distan	Domain_scor	Annotated_gene_function
AFLA_11689	AFLA_11692	-3	2842	120	309649	316354	2049	1	oxidoreductase, short chain dehydrogenase/reductase family protein
AFLA_11689	AFLA_11691	-2	2842	119	307036	307600	1562	0	hypothetical protein
AFLA_11689	AFLA_11690	-1	2842	118	305474	304762	359	0	hypothetical protein
AFLA_11689	AFLA_11689	0	2842	117	296613	304403	0	0	polyketide synthase, putative
AFLA_11689	AFLA_11688	1	2842	116	294100	295920	693	1	New cDNA-based gene: (AO_CDS_042706, novel, updateIDs: 10705, [gene: novel_gene_1117, model: novel_model_111
AFLA_11689	AFLA_11687	2	2842	115	291683	290319	2417	1	Transferase family protein
AFLA_11689	AFLA_11686	3	2842	114	289659	287442	660	0	alpha-N-acetylglucosaminidase, putative
AFLA_11689	AFLA_11685	4	2842	113	287114	285858	328	0	hypothetical protein
AFLA_11689	AFLA_11684	5	2842	112	284401	282903	1457	1	FAD binding domain containing protein
AFLA_11689	AFLA_11683	6	2842	111	277662	282510	393	0	oxidoreductase, 2-nitropropane dioxygenase family protein
AFLA_11689	AFLA_11682	7	2842	110	274509	269808	3153	1	4'-phosphopantetheinyl transferase superfamily protein

Cluster:44

Backbone_ge	Gene_id	Gene_positic	Chromosome	Gene_order	5'end	3'end	Gene_distan	Domain_scor	Annotated_gene_function
AFLA_11844	AFLA_11846	-2	2842	274	724528	717381	306	1	Major Facilitator Superfamily protein
AFLA_11844	AFLA_11845	-1	2842	273	717075	714970	1442	0	conserved hypothetical protein
AFLA_11844	AFLA_11844	0	2842	272	713528	710225	0	0	NRPS-like enzyme, putative

Cluster:45

Backbone_ge	Gene_id	Gene_positic	Chromosome	Gene_order	5'end	3'end	Gene_distan	Domain_scor	Annotated_gene_function
AFLA_11894	AFLA_11900	-6	2842	328	864460	865900	1728	1	O-methyltransferase family protein
AFLA_11894	AFLA_11899	-5	2842	327	860771	862732	988	1	Major Facilitator Superfamily protein
AFLA_11894	AFLA_11898	-4	2842	326	859783	858715	128	0	Mitochondrial carrier protein
AFLA_11894	AFLA_11897	-3	2842	325	857064	858587	723	1	FAD dependent oxidoreductase family protein
AFLA_11894	AFLA_11896	-2	2842	324	856341	849580	317	0	polyketide synthase, putative
AFLA_11894	AFLA_11895	-1	2842	323	849263	848472	807	0	candidate tumor suppressor in ovarian cancer 2-related
AFLA_11894	AFLA_11894	0	2842	322	847665	840026	0	1	polyketide synthase, putative
AFLA_11894	AFLA_11893	1	2842	321	837504	837923	2103	0	hypothetical protein
AFLA_11894	AFLA_11892	2	2842	320	836981	837268	236	0	hypothetical protein
AFLA_11894	AFLA_11891	3	2842	319	835803	836674	307	0	hypothetical protein
AFLA_11894	AFLA_11890	4	2842	318	833331	834827	976	0	conserved hypothetical protein
AFLA_11894	AFLA_11889	5	2842	317	831650	832296	1035	0	translation initiation factor eIF-5A family protein
AFLA_11894	AFLA_11888	6	2842	316	829161	830552	1098	1	conserved hypothetical protein
AFLA_11894	AFLA_11887	7	2842	315	828567	828080	594	0	Endoribonuclease L-PSP family protein
AFLA_11894	AFLA_11886	8	2842	314	827637	827912	168	0	New cDNA-based gene: (AO_CDS_042706, novel, updateIDs: 10859, [gene: novel_gene_1135, model: novel_model_113
AFLA_11894	AFLA_11885	9	2842	313	826960	827451	186	0	hypothetical protein
AFLA_11894	AFLA_11884	10	2842	312	826054	824528	906	1	conserved hypothetical protein
AFLA_11894	AFLA_11883	11	2842	311	822272	824353	175	0	Zinc finger, C2H2 type family protein
AFLA_11894	AFLA_11882	12	2842	310	821202	820180	1070	1	oxidoreductase, short chain dehydrogenase/reductase family protein

Cluster:46

Backbone_ge	Gene_id	Gene_positic	Chromosome	Gene_order	5'end	3'end	Gene_distan	Domain_scor	Annotated_gene_function
AFLA_11911	AFLA_11914	-3	2842	342	906069	906963	3131	1	NAD dependent epimerase/dehydratase family protein
AFLA_11911	AFLA_11913	-2	2842	341	902211	902938	469	0	hypothetical protein
AFLA_11911	AFLA_11912	-1	2842	340	900338	901742	578	0	beta-lactamase family protein
AFLA_11911	AFLA_11911	0	2842	339	896447	899760	0	0	NRPS-like enzyme, putative
AFLA_11911	AFLA_11910	1	2842	338	896116	896393	54	0	New cDNA-based gene: (AO_CDS_042706, novel, updateIDs: 10881, [gene: novel_gene_1138, model: novel_model_113
AFLA_11911	AFLA_11909	2	2842	337	895578	894408	538	1	NAD dependent epimerase/dehydratase family protein

Cluster:47

Backbone_ge	Gene_id	Gene_positic	Chromosome	Gene_order	5'end	3'end	Gene_distan	Domain_scor	Annotated_gene_function
AFLA_12152	AFLA_12154	-2	2842	582	1485551	1487055	1301	1	Major Facilitator Superfamily protein
AFLA_12152	AFLA_12153	-1	2842	581	1483117	1484250	727	0	oxidoreductase, FAD/FMN-binding family protein
AFLA_12152	AFLA_12152	0	2842	580	1482390	1479298	0	0	NRPS-like enzyme, putative
AFLA_12152	AFLA_12151	1	2842	579	1477769	1476852	1529	0	methyltransferase LaeA-like, putative
AFLA_12152	AFLA_12150	2	2842	578	1474631	1476355	497	1	Cytochrome P450 family protein
AFLA_12152	AFLA_12149	3	2842	577	1474181	1472992	450	0	NmrA-like family protein
AFLA_12152	AFLA_12148	4	2842	576	1471814	1472787	205	1	hypothetical protein

Cluster:48

Backbone_ge	Gene_id	Gene_positic	Chromosome	Gene_order	5'end	3'end	Gene_distan	Domain_scor	Annotated_gene_function
AFLA_12709	AFLA_12711	-2	2856	408	1043171	1041460	182	1	Major Facilitator Superfamily protein
AFLA_12709	AFLA_12710	-1	2856	407	1040353	1041278	657	0	hypothetical protein
AFLA_12709	AFLA_12709	0	2856	406	1031825	1039696	0	0	polyketide synthase, putative
AFLA_12709	AFLA_12708	1	2856	405	1030106	1031122	703	0	NmrA-like family protein
AFLA_12709	AFLA_12707	2	2856	404	1029840	1028933	266	1	oxidoreductase, short chain dehydrogenase/reductase family protein
AFLA_12709	AFLA_12706	3	2856	403	1027377	1028585	348	0	hypothetical protein
AFLA_12709	AFLA_12705	4	2856	402	1026663	1026334	714	0	hypothetical protein
AFLA_12709	AFLA_12704	5	2856	401	1024714	1026015	319	1	To monocarboxylate transporter, putative
AFLA_12709	AFLA_12703	6	2856	400	1023836	1023033	878	0	unknown-related
AFLA_12709	AFLA_12702	7	2856	399	1022410	1020928	623	1	FAD binding domain containing protein
AFLA_12709	AFLA_12701	8	2856	398	1020241	1020699	229	0	hypothetical protein

AFLA_12709	AFLA_12700	9	2856	397	1019062	1019679	562	0	hypothetical protein
AFLA_12709	AFLA_12699	10	2856	396	1017582	1018880	182	1	Fungal specific transcription factor domain containing protein
AFLA_12709	AFLA_12698	11	2856	395	1014062	1015736	1846	0	hypothetical protein
AFLA_12709	AFLA_12697	12	2856	394	1012031	1013848	214	1	Amino acid permease family protein

Cluster:49

Backbone_ge	Gene_id	Gene_positio	Chromosome	Gene_order	5'end	3'end	Gene_distan	Domain_scor	Annotated_gene_function
AFLA_12806	AFLA_12809	-3	2856	506	1328474	1330062	775	1	Cytochrome P450 family protein
AFLA_12806	AFLA_12808	-2	2856	505	1326250	1327699	705	0	conserved hypothetical protein
AFLA_12806	AFLA_12807	-1	2856	504	1325545	1324111	1112	0	conserved hypothetical protein
AFLA_12806	AFLA_12806	0	2856	503	1314426	1322999	0	1	polyketide synthase, putative
AFLA_12806	AFLA_12805	1	2856	502	1313485	1312404	941	0	DUF341 domain oxidoreductase, putative
AFLA_12806	AFLA_12804	2	2856	501	1312172	1310950	232	1	Major Facilitator Superfamily protein

Cluster:50

Backbone_ge	Gene_id	Gene_positio	Chromosome	Gene_order	5'end	3'end	Gene_distan	Domain_scor	Annotated_gene_function
AFLA_12817	AFLA_12817	0	2856	514	1361595	1364900	0	1	NRPS-like enzyme, putative
AFLA_12817	AFLA_12816	1	2856	513	1357969	1360564	1031	1	Fungal specific transcription factor domain containing protein
AFLA_12817	AFLA_12815	2	2856	512	1355550	1356233	1736	1	hypothetical protein

Cluster:51

Backbone_ge	Gene_id	Gene_positio	Chromosome	Gene_order	5'end	3'end	Gene_distan	Domain_scor	Annotated_gene_function
AFLA_12993	AFLA_12993	0	2856	690	1853766	1855767	0	1	3-oxoacyl carrier protein synthase, putative
AFLA_12993	AFLA_12992	1	2856	689	1853373	1852018	393	0	agmatinase, putative
AFLA_12993	AFLA_12991	2	2856	688	1849649	1850619	1399	0	RAS small monomeric GTPase Rab6, putative
AFLA_12993	AFLA_12990	3	2856	687	1845774	1847810	1839	1	MFS siderophore transporter, putative

Cluster:52

Backbone_ge	Gene_id	Gene_positio	Chromosome	Gene_order	5'end	3'end	Gene_distan	Domain_scor	Annotated_gene_function
AFLA_13549	AFLA_13549	0	2911	441	1209037	1212252	0	0	nonribosomal peptide synthase, putative
AFLA_13549	AFLA_13548	1	2911	440	1207576	1208112	925	0	conserved hypothetical protein
AFLA_13549	AFLA_13547	2	2911	439	1206763	1205330	813	0	Pyridoxal-dependent decarboxylase, pyridoxal binding domain containing protein
AFLA_13549	AFLA_13546	3	2911	438	1204397	1203084	933	0	hypothetical protein
AFLA_13549	AFLA_13545	4	2911	437	1200602	1201774	1310	0	hypothetical protein
AFLA_13549	AFLA_13544	5	2911	436	1199997	1198143	605	1	Cytochrome P450 family protein
AFLA_13549	AFLA_13543	6	2911	435	1197282	1195553	861	1	Cytochrome P450 family protein
AFLA_13549	AFLA_13542	7	2911	434	1194144	1195202	351	0	NmrA-like family protein
AFLA_13549	AFLA_13541	8	2911	433	1193172	1190701	972	0	conserved hypothetical protein
AFLA_13549	AFLA_13540	9	2911	432	1187408	1185764	3293	1	hypothetical protein

Cluster:53

Backbone_ge	Gene_id	Gene_positio	Chromosome	Gene_order	5'end	3'end	Gene_distan	Domain_scor	Annotated_gene_function
AFLA_13787	AFLA_13792	-5	2911	684	1887924	1886127	244	1	Major Facilitator Superfamily protein
AFLA_13787	AFLA_13791	-4	2911	683	1884679	1885883	2354	0	monooxygenase-related
AFLA_13787	AFLA_13790	-3	2911	682	1881887	1882325	1427	0	hypothetical protein
AFLA_13787	AFLA_13789	-2	2911	681	1879744	1880460	1736	0	hypothetical protein
AFLA_13787	AFLA_13788	-1	2911	680	1876855	1878008	1382	0	Lipase family protein
AFLA_13787	AFLA_13787	0	2911	679	1868122	1875473	0	1	polyketide synthase, putative
AFLA_13787	AFLA_13786	1	2911	678	1865851	1867182	940	0	hypothetical protein
AFLA_13787	AFLA_13785	2	2911	677	1864603	1863441	1248	0	hypothetical protein
AFLA_13787	AFLA_13784	3	2911	676	1862418	1861633	1023	0	hypothetical protein
AFLA_13787	AFLA_13783	4	2911	675	1861345	1860472	288	0	To negative acting factor
AFLA_13787	AFLA_13782	5	2911	674	1857823	1859636	836	1	metallo-beta-lactamase superfamily protein, putative
AFLA_13787	AFLA_13781	6	2911	673	1857270	1855677	553	1	Major Facilitator Superfamily protein

AFLA_13787	AFLA_13780	7	2911	672	1854366	1855107	570	0	hypothetical protein
AFLA_13787	AFLA_13779	8	2911	671	1853040	1853610	756	0	hypothetical protein
AFLA_13787	AFLA_13778	9	2911	670	1850177	1852591	449	1	ABC-2 type transporter family protein

Cluster:54

Backbone_ge	Gene_id	Gene_positio	Chromosome	Gene_order	5'end	3'end	Gene_distan	Domain_scor	Annotated_gene_function
AFLA_13941	AFLA_13950	-9	2911	841	2278117	2279969	440	1	Fungal specific transcription factor domain containing protein
AFLA_13941	AFLA_13949	-8	2911	840	2265957	2277677	979	1	hybrid PKS/NRPS enzyme, putative
AFLA_13941	AFLA_13948	-7	2911	839	2264978	2263609	499	0	dimethylallyl tryptophan synthase, putative
AFLA_13941	AFLA_13947	-6	2911	838	2261743	2263110	3110	0	hypothetical protein
AFLA_13941	AFLA_13946	-5	2911	837	2258633	2257028	2329	1	major facilitator superfamily protein
AFLA_13941	AFLA_13945	-4	2911	836	2254023	2254699	921	0	New cDNA-based gene: (AO_CDS_042706, novel, updateIDs: 12573, [gene: novel_gene_1311, model: novel_model_131
AFLA_13941	AFLA_13944	-3	2911	835	2252340	2253102	18	0	aflF / norB / dehydrogenase
AFLA_13941	AFLA_13943	-2	2911	834	2252322	2250833	371	0	aflU / cypA / P450 monooxygenase
AFLA_13941	AFLA_13942	-1	2911	833	2250462	2248242	1659	1	aflT / aflT / transmembrane protein
AFLA_13941	AFLA_13941	0	2911	832	2239960	2246583	0	0	aflC / pksA / pksL1 / poleketide synthase
AFLA_13941	AFLA_13940	1	2911	831	2239550	2238777	410	0	aflCa / hypC / hypothetical protein
AFLA_13941	AFLA_13939	2	2911	830	2238324	2237281	453	1	aflD / nor-1 / reductase
AFLA_13941	AFLA_13938	3	2911	829	2230826	2235970	1311	1	aflA / fas-2 / hexA / fatty acid synthase alpha subunit
AFLA_13941	AFLA_13937	4	2911	828	2230136	2224290	690	0	aflB / fas-1 / fatty acid synthase beta subunit
AFLA_13941	AFLA_13936	5	2911	827	2221686	2223020	1270	1	aflR / apa-2 / afl-2 / transcription activator
AFLA_13941	AFLA_13934	6	2911	826	2220947	2219496	739	0	aflS/ pathway regulator
AFLA_13941	AFLA_13933	7	2911	825	2218928	2217954	568	1	aflH/ adhA/ short chain alcohol dehydrogenase
AFLA_13941	AFLA_13932	8	2911	824	2217172	2216171	782	0	aflJ/ estA/ esterase
AFLA_13941	AFLA_13931	9	2911	823	2215880	2214407	291	0	aflE/ norA/ aad/ adh-2/ NOR reductase/ dehydrogenase
AFLA_13941	AFLA_13930	10	2911	822	2213781	2212881	626	1	aflM/ ver-1/ dehydrogenase/ ketoreductase
AFLA_13941	AFLA_13929	11	2911	821	2212268	2212651	230	0	aflMa/ hypE/ hypothetical protein
AFLA_13941	AFLA_13928	12	2911	820	2212022	2210319	246	1	aflN/ verA/ monooxygenase
AFLA_13941	AFLA_13927	13	2911	819	2209882	2209493	437	0	aflNa/ hypD/ hypothetical protein
AFLA_13941	AFLA_13926	14	2911	818	2207673	2209264	229	1	aflG/ avnA/ ord-1/ cytochrome P450 monooxygenase
AFLA_13941	AFLA_13925	15	2911	817	2205644	2207305	368	1	aflL/ verB/ desaturase/ P450 monooxygenase
AFLA_13941	AFLA_13924	16	2911	816	2204914	2205455	189	0	aflLa/ hypB/ hypothetical protein
AFLA_13941	AFLA_13923	17	2911	815	2203883	2204843	71	0	aflI/ avfA/ cytochrome P450 monooxygenase
AFLA_13941	AFLA_13922	18	2911	814	2202376	2203709	174	1	aflO/ omtB/ dmtA/ O-methyltransferase B
AFLA_13941	AFLA_13921	19	2911	813	2199609	2201242	1134	1	aflP/ omtA/ omt-1/ O-methyltransferase A
AFLA_13941	AFLA_13920	20	2911	812	2198596	2196236	1013	1	aflQ/ ordA/ ord-1/ oxidoreductase/ cytochrome P450 monooxigenase

Cluster:55

Backbone_ge	Gene_id	Gene_positio	Chromosome	Gene_order	5'end	3'end	Gene_distan	Domain_scor	Annotated_gene_function
AFLA_13967	AFLA_13966	1	2911	857	2328209	2328721	103	0	hypothetical protein
AFLA_13967	AFLA_13965	2	2911	856	2326370	2326089	1839	0	hypothetical protein
AFLA_13967	AFLA_13964	3	2911	855	2324840	2324460	1249	0	hypothetical protein
AFLA_13967	AFLA_13963	4	2911	854	2320176	2324124	336	1	ABC multidrug transporter, putative
AFLA_13967	AFLA_13962	5	2911	853	2316403	2318184	1992	1	Amino acid permease family protein
AFLA_13967	AFLA_13961	6	2911	852	2314230	2311922	2173	0	conserved hypothetical protein
AFLA_13967	AFLA_13960	7	2911	851	2310748	2311455	467	0	hypothetical protein
AFLA_13967	AFLA_13959	8	2911	850	2309635	2310636	112	0	hypothetical protein
AFLA_13967	AFLA_13958	9	2911	849	2307690	2306248	1945	1	gluconolactone oxidase, putative
AFLA_13967	AFLA_13957	10	2911	848	2303196	2303633	2615	0	hypothetical protein
AFLA_13967	AFLA_13956	11	2911	847	2302374	2300224	822	1	Fungal specific transcription factor domain containing protein
