# Supplementary material for: The KdmB-EcoA-RpdA-SntB (KERS) chromatin regulatory complex controls development, secondary metabolism and pathogenicity in Aspergillus flavus
Source: Fungal Genet Biol. Author manuscript; Available in PMC 2024 Feb 5. (PMC10841535; doi:10.1016/j.fgb.2023.103836)
Supplement: supplemental4 [file NIHMS1938650-supplement-supplemental4.docx]

| **Table S4**. Strains used in this study | |  |
| --- | --- | --- |
|  |  |  |
| **strain** | Genotype | **Reference** |
| NRRL3357 | *nku70Δ::argB* | Keller Lab. |
| Tjes19.1 | *nku70Δ::argB, pyrG89* | Pfannenstiel et al., 2018 |
| AFLBK1 | *kdmB::3xHA::pyrG, nku70Δ* | This study |
| AFLBK2 | *kdmB::sgfp::pyrG, nku70Δ* | This study |
| AFLBK22 | *kdmBΔ::pyrG, nku70Δ* | This study |
| AFLBK23 | *rpdAΔ::pyrG, nku70Δ* | This study |
| AFLBK6.10 | *kdmBΔ::pyrG; kdmB::phleO; nkuAΔ::argB* | This study |
| AFLBK7.4 | *rpdAΔ::pyrG; rpdA::phleO; nkuAΔ::argB* | This study |
