# Supplementary material for: The KdmB-EcoA-RpdA-SntB (KERS) chromatin regulatory complex controls development, secondary metabolism and pathogenicity in Aspergillus flavus
Source: Fungal Genet Biol. Author manuscript; Available in PMC 2024 Feb 5. (PMC10841535; doi:10.1016/j.fgb.2023.103836)
Supplement: supplemental5 [file NIHMS1938650-supplement-supplemental5.docx]

| **Table S5**. Oligonucleotides used in this study | |
| --- | --- |
|  |  |
| **Designation** | Sequence in 5' > 3' direction |
| BK67 (5UTR AFL8211 pUC tail) | TTCGAGCTCGGTACCCagacgatcggtttgctggcac |
| BK68 (5UTR AFL8211 nest) | agacgatcggtttgctggcac |
| BK69 (5UTR AFL8211 pyrG tail) | GAGCATTGTTTGAGGCgctaatgaggctctggaagtact |
| BK70 (3UTR AFL8211 pyrG tail) | GCCTCCTCTCAGACAGttcttaaatgcgacgatctgatcgt |
| BK71 (3UTR AFL8211 pUC tail) | ACTCTAGAGGATCCCCtttatttccccctcctatggtgc |
| BK72 (3UTR AFL8211 nest) | tttatttccccctcctatggtgc |
| BK73 (5UTR AFL RpdA pUC tail) | TTCGAGCTCGGTACCCctgggtgttgatatcgacagatg |
| BK74 (5UTR AFL RpdA nest) | ctgggtgttgatatcgacagatg |
| BK75 (5UTR AFL RpdA pyrG tail) | GAGCATTGTTTGAGGCcttggatgacaatgtatgagagag |
| BK76 (3UTR AFL RpdA pyrG tail) | GCCTCCTCTCAGACAGgctccgctttttagaggcgttta |
| BK77 (3UTR AFL RpdA pUC tail) | ACTCTAGAGGATCCCCacggtggccatgaccatgtca |
| BK78 (3UTR AFL RpdA nest) | acggtggccatgaccatgtca |
| BK182 (AFL 8211 5UTR OUT) | gccctactggattccttcgg |
| BK184 (AFL RpdA/Rpd3 5UTR OUT) | gttgtgctcgccttcctcatg |
| BK212 (pyroA control RVS) | CACCACCCTTCAGCATCTGAG |
| BK213 (pyroA control FWD) | GGTATCAATGTCTCTCAGATGCC |
| BK214 (AN8211 5UTR OUT) | GAT GGG GTG AAT GGG TTG AAC |
| BK250 (AFL kdmB cDNA FWD) | CGTGGTGAACCTACATTGGC |
| BK251 (AFL kdmB cDNA RVS) | CCGGTGTAGAATCGCTTGC |
| BK254 (AFL rpdA cDNA FWD) | GTCAAGAGCAAGCAGCTCC |
| BK255 (AFL rpdA cDNA RVS) | GATTGCTCCTGCTGCTCTG |
| BK363 (AFL kdmB 5UTR pan8-1 tail) | cccaagaccgacaagggatagttcgacggtggataacag |
| BK364 (AFL kdmB 3UTR pan8-1 tail) | gcgttctggagggaggcggagaagtcatcgtatctctc |
| BK365 (AFL rpd3 5UTR pan8-1 tail) | cccaagaccgacaaggcgacctttccgacatatcttgtc |
| BK366 (AFL rpd3 5UTR pan8-1 tail) | gcgttctggagggaggagttggaagcgatacgctgttg |
| BK399 (AFLkdmB ORF pUC tail) | TTCGAGCTCGGTACCCccagtctctgtatgagagaagc |
| BK400 (AFLkdmB ORF linker tail) | CACCGCTACCACCTCCcaaatcctctgtactgcgatcg |
| BK401 (AFLkdmB 3UTR pyrG/pyroA tail) | GCCTCCTCTCAGACAGcgctgttttaaagtataatccctatc |
| BK402 (AFLkdmB 3UTR pUC tail) | ACTCTAGAGGATCCCCgatagttcgacggtggataacag |
| BK403 (AFLkdmB 5UTR nest) | ccagtctctgtatgagagaagc |
| BK404 (AFLkdmB 3UTR nest) | gatagttcgacggtggataacag |
| BK417 (AFLrpd3 5UTR pUC tail) | TTCGAGCTCGGTACCCcgctatcggtgctctctcatac |
| BK418 (AFLrpd3 ORF linker tail) | CACCGCTACCACCTCCtgcctcgctcttggtgggc |
| BK419 (AFLrpd3 3UTR pyrG tail) | GCCTCCTCTCAGACAGgctccgctttttagaggcgttt |
| BK420 (AFLrpd3 3UTR pUC tail) | ACTCTAGAGGATCCCCcttgctctgacggacgacaac |
| BK421 (AFLrpd3 5UTR F) | cgctatcggtgctctctcatac |
| BK422 (AFLrpd3 3UTR R) | cttgctctgacggacgacaac |
| BK431 (AFLrpd3 F qPCR) | GACGAAGAAGAGAACGCTGC |
| BK432 (AFLrpd3 R qPCR) | GTAGTATCCATGTCATCGTCAG |
| BK461 (AFLkdmB 5UTR for comp) | cccaagaccgacaagggacccgattacgcctattctgg |
| BK462 (AFLkdmB 3UTR for comp) | gcgttctggagggagggtttgtggtgtttgtggaaagatag |
| BK463 (AFLkdmB ORF F pOB110tail) | GCAGACATCACCGTTTatggtggctccggcctcg |
| BK464 (AFLkdmB ORF R pOB110tail) | GATAGACATGGCGTTTttacaaatcctctgtactgcgatcg |
| brlA-F (AFLA qPCR) | TATCCAGACATTCAAGACGCACAG |
| brlA-R (AFLA qPCR) | GATAATAGAGGGCAAGTTCTCCAAAG |
| abaA-F (AFLA qPCR) | GAGTGGCAGACCGAATGTATGTTG |
| abaA-R (AFLA qPCR) | TAGTGGTAGGCATTGGGTGAGTTG |
| aflR-F (AFLA qPCR) | GCAACCTGATGACGACTGATATGG |
| aflR-R (AFLA qPCR) | TGCCAGCACCTTGAGAACGATAAG |
| BK471 (AFLA nsdC cDNA F) | CAGCCATTCTAGCAACCATAAC |
| BK472 (AFLA nsdC cDNA R) | TCTCGCTCACGATTCTGATC |
| BK473 (AFLA nsdD cDNA F) | CAATGTACCAAGACGAATACAAG |
| BK474 (AFLA nsdD cDNA R) | TGTCTCAGCTCGGTTACAAC |
| aflA-F (AFLA qPCR) | CCTATAAGTGCTTCAAAGATCGTGATCG |
| aflA-R (AFLA qPCR) | CGTACATGGATGACACGTTGTCCCAG |
| aflC-F (AFLA qPCR) | CCTATTCTAGCCGCCTTTCTTGAC |
| aflC-R (AFLA qPCR) | CATGTTGCCAGATTCCTCATATTCC |
| aflD-F (AFLA qPCR) | TGTATGCTCCCGTCCTACTGTTTC |
| aflD-R (AFLA qPCR) | TGTAGTCTCCTTAGTCGCTTCATC |
| aflM-F (AFLA qPCR) | GCGGAGAAAGTGGTTGAACAGATC |
| aflM-R (AFLA qPCR) | CAGCGAACAAAGGTGTCAATAGCC |
| aflP-F (AFLA qPCR) | CGATGTCTATCTTCTCCGATCTATTC |
| aflP-R (AFLA qPCR) | TCTCAGTCTCCAGTCTATTATCTACC |
| BK585 (AFLA flbA cDNA F) | CACTGCGCAACAGCTTGG |
| BK586 (AFLA flbA cDNA R) | GATCGAATCACTTGACATCAAC |
| BK589 (AFLA flbC cDNA F) | CATGATGAGCCAGTTCAGTTC |
| BK590 (AFLA flbC cDNA R) | CACCAGTGTGACTGTACATG |
| BK603_AFCL1F | GGAAGCAAGAGAGATTATCCTC |
| BK604_AFCL1R | CGAGTGAGTCAATTCCTAGTTC |
| BK605_AFCL2F | CATGCTGCACAGTGGTATCC |
| BK606_AFCL2R | CTTCCAGTTCAGTGAACTCTG |
| BK607_AFCL3F | GGAGATCAGTCCTGAGAAGC |
| BK608_AFCL3R | CTACTCATGATATCAAAGATGACG |
| BK609_AFCL4F | CAAGTCAGCATGGTTGACATTC |
| BK610_AFCL4R | TCGTCGCATCTTGTTCCGAG |
| BK611_AFCL5F | CCATTCAAAGACGAGAAATGGG |
| BK612_AFCL5R | CAATGTATCCCACTTGTTAGGTC |
| BK613_AFCL6F | CAGACTATCTTCGAAACCTCG |
| BK614_AFCL6R | GACCTGCTGGTCTGTACAG |
| BK615_AFCL7F | CTCGACCACATTTGAGTTATCG |
| BK616_AFCL7R | CCTCTTTGCTCTACCAAGCC |
| BK617_AFCL8F | GTTGATATTCTGAACCCAGATG |
| BK618_AFCL8R | GGCAGCCAACTCATCAAGG |
| BK619_AFCL9F | CTACAATGTCCAGCCAATTGAG |
| BK620_AFCL9R | CCTTCCACTAGCCCTTGG |
| BK621_AFCL10F | GGATTGGTTGGAGATACTATCG |
| BK622_AFCL10R | CATCGTAAGTGGCCATGTCC |
| BK623_AFCL11F | CTGTGATGCCACTCCTTCAG |
| BK624_AFCL11R | GAATATCCGGTGCATCCTTC |
| BK625_AFCL12F | CTGATGATGCGAAGTCCACG |
| BK626_AFCL12R | GATGGAGTCCAAGACAATCC |
| BK627_AFCL13F | CTAGCGTTATAGTACGGATTGG |
| BK628_AFCL13R | TGCTAAGGGAGTCAGGCAG |
| BK629_AFCL14F | GATCAACAATAAAGACCAGGATAG |
| BK630_AFCL14R | GTAATGAAGTGGTGGATTCCTG |
| BK631_AFCL15F | CAGTTCCATTCTGGCGGAG |
| BK632_AFCL15R | GGTGATCAGTCATCGCTCG |
| BK633_AFCL16F | CAGGGAACTGATACTAAGACC |
| BK634_AFCL16R | GATCGACCTGCTTTCCACTG |
| BK637_AFCL18F | GTTGATGCTGGTAAGAAGCG |
| BK638_AFCL18R | AGGTAGGAGTCGTCAAGTTC |
| BK639_AFCL19F | GTTTCGCTCGTTAGACCAGG |
| BK640_AFCL19R | CAGCGACCAGGGTGGTAG |
| BK641_AFCL20F | CATCGTCAAGTCAGCGCAG |
| BK642_AFCL20R | CGTCTCTTCGTCTCCGATG |
| BK643_AFCL21F | CCTGAATCAGGATGATCTAGC |
| BK644_AFCL21R | GTGATATGCGTAAGTTGTACCTC |
| BK645_AFCL22F | ACTCCGGACCTGCTACAC |
| BK646_AFCL22R | AGAAGCTGTATATTCTCTTCCG |
| BK647_AFCL23F | CAGCGAGCGATATCTGGAG |
| BK648_AFCL23R | AGGATCGCATTCAAGGCATC |
| BK649_AFCL24F | CTACTTTCTTGGGCCATTTGG |
| BK650_AFCL24R | GAGACAGACGGAACACGAG |
| BK651_AFCL25F | GTTATCAACGATGCAGGGTAC |
| BK652_AFCL25R | CGGAACATCGGTAACAGAGG |
| BK653_AFCL26F | CCTGGCCTTGGTGTCTAC |
| BK654_AFCL26R | GTCCGTAGACATGTTGAGAAG |
| BK655_AFCL27F | GTGAACAGTCTACATACATACC |
| BK656_AFCL27R | CAATCCCTCTGGGTCATCG |
| BK657_AFCL28F | CCATCAACAGAACTAATTCCAG |
| BK658_AFCL28R | CGTACTAGAAGGATTGACATGG |
| BK659_AFCL29F | CCGTCGCTACATTCTGTGC |
| BK660_AFCL29R | GATACCAATGATTGAAGATGGTTC |
| BK661_AFCL30F | GGATAGAGAGCTTCAGATCAAAG |
| BK662_AFCL30R | TGGTCAACGGCGATGGATC |
| BK663_AFCL31F | CTCCCTCATTGAGATATTACGA |
| BK664_AFCL31R | CAACTCCTTAAAGCAGAGACTC |
| BK665_AFCL32F | GTCCTTCTCGACAGATCATG |
| BK666_AFCL32R | CGTGCTGGAAGAGCTGTC |
| BK667_AFCL33F | GGAAACGAATCTTCCGTCCG |
| BK668_AFCL33R | ATATTCTCGTACAGCTCCCAC |
| BK669_AFCL34F | GTGACTCCAGAGGATATCTC |
| BK670_AFCL34R | TGGCAGAAGCTCTCCATCAC |
| BK671_AFCL35F | AGTGGATTCGACTTCCAGAC |
| BK672_AFCL35R | TTCTCTGTGACGAAGGTCTTC |
| BK673_AFCL36F | CAAGAGCTGTGTCTGCTAATC |
| BK674_AFCL36R | CTAGCTGCATATTACACCGAAC |
| BK675_AFCL37F | GTCACAGCAAGATGAGTCAG |
| BK676_AFCL37R | GGTCTCGGATGCGATTAGAG |
| BK677_AFCL38F | GATGTTAGCGACAATGCTGAG |
| BK678_AFCL38R | GAGGTATCCAAGGCAGACAC |
| BK679_AFCL39F | AGAACGATTGGTTGCAGGATC |
| BK680_AFCL39R | GCAACGTCGAAGACCTGG |
| BK681_AFCL40F | CTTCCTCTGCCTCGAAACC |
| BK682_AFCL40R | CATATTGCTCAGTGGCTCTTG |
| BK683_AFCL41F | CCTTCAATGATGTACTAAGCCAG |
| BK684_AFCL41R | GGGTCCCTTGGCGACTTG |
| BK685_AFCL42F | GACCAGATGCAGGACCAAG |
| BK686_AFCL42R | GGAATCTCCTGACACATGATG |
| BK687_AFCL43F | GTGGAGTTATTGCTGACTGAC |
| BK688_AFCL43R | GACAATATTACCGGTAGGAATAC |
| BK689_AFCL44F | CTTCCACCTTATGAACCCTAATC |
| BK690_AFCL44R | CTTGATAGATTCCAGATCACTGAT |
| BK691_AFCL45F | GTCTAGTGTTGAAAGAGCAGC |
| BK692_AFCL45R | TGACTGCTTCCACCAATTGC |
| BK693_AFCL46F | CAGAGGTTGTCGATATGCATG |
| BK694_AFCL46R | GTAAAGGTACCATGGTTGCTG |
| BK695_AFCL47F | GTTGACTACGTTAACTCGGTC |
| BK696_AFCL47R | ACAGGTCCATTGTGGCATCC |
| BK697_AFCL48F | GATCTCGACGGTGTTCTATC |
| BK700_AFCL49R | TGCAACCAGCGAATCAACAC |
| BK701_AFCL50F | CAACGAGACTGGATCCACC |
| BK702_AFCL50R | TCAGTATGCCAGTTGAACGAC |
| BK703_AFCL51F | GTATGACCATCTGTGCTGTG |
| BK704_AFCL51R | GTTGGTCAATGCCACATCTATC |
| BK705_AFCL52F | GTTTCGAGAGCGGGACATG |
| BK706_AFCL52R | CTCCATCCATCGTGCTCTC |
| BK709_AFCL54F | CGTCCTACTTAATCCCACAC |
| BK710_AFCL54R | CTCGTCCATGACTGTATCTG |
| BK711_AFCL55F | CAATTCAACCCGAGATTCGAC |
| BK712_AFCL55R | CTCACAAGGGAGCCAATTATAC |
