# Supplementary material for: The KdmB-EcoA-RpdA-SntB (KERS) chromatin regulatory complex controls development, secondary metabolism and pathogenicity in Aspergillus flavus
Source: Fungal Genet Biol. Author manuscript; Available in PMC 2024 Feb 5. (PMC10841535; doi:10.1016/j.fgb.2023.103836)
Supplement: supplemental6 [file NIHMS1938650-supplement-supplemental6.docx]

| Table S6. Plasmids used in this study | |  |
| --- | --- | --- |
|  |  |  |
| **Plasmid** | **Description** | **Reference** |
| pUC19 | *E. coli* cloning plasmid with *bla* (ampicillin resistance gene) gene | Thermo Fisher |
| pBK22 | *kdmB* deletion cassette with *AfpyrG* in *Sma*I site of pUC19 | This study |
| pBK23 | *rpdA deletion cassette with AfpyrG in SmaI site of pUC19* | This study |
| pBK66 | *kdmB* genomic locus in *StuI* site of pan8-1 | This study |
| pBK67 | *rpdA* genomic locus in *StuI* site of pan8-1 | This study |
| pBK79 | *kdmB::sgfp::AfpyrG* cassette in *Sma*I site of pUC19 | This study |
| pBK80 | *kdmB::3xha::AfpyrG* cassette in *Sma*I site of pUC19 | This study |
| pOB430 | 3X HA with GGGSGG linker trpC pyrG in SmaI of pUC19 | This study |
