# Supplementary figures and images for: The KdmB-EcoA-RpdA-SntB (KERS) chromatin regulatory complex controls development, secondary metabolism and pathogenicity in Aspergillus flavus
Source: Fungal Genet Biol. Author manuscript; Available in PMC 2024 Feb 5. (PMC10841535; doi:10.1016/j.fgb.2023.103836)

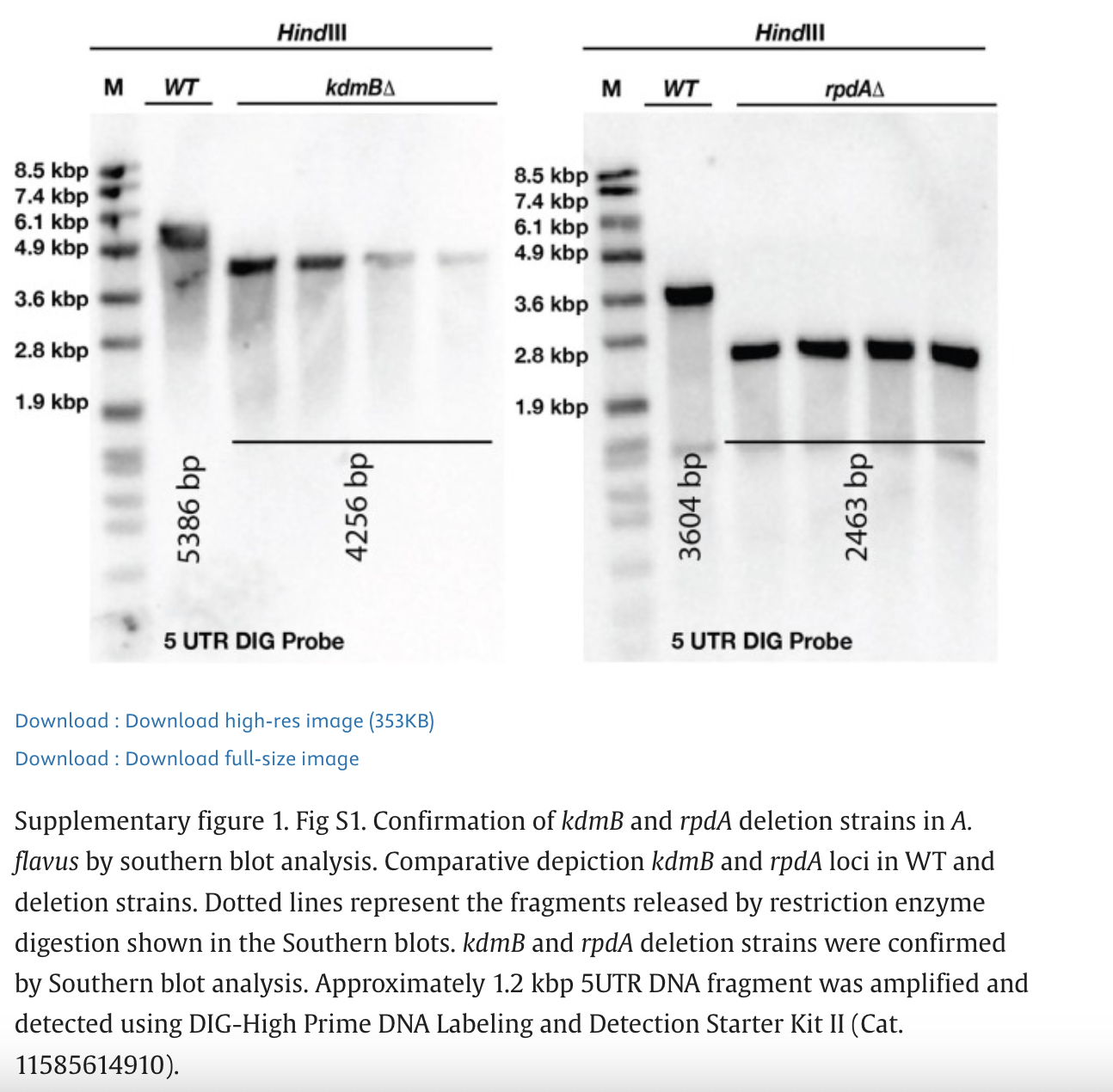

Supplement: figure S1 [file NIHMS1938650-supplement-figure_S1.png]

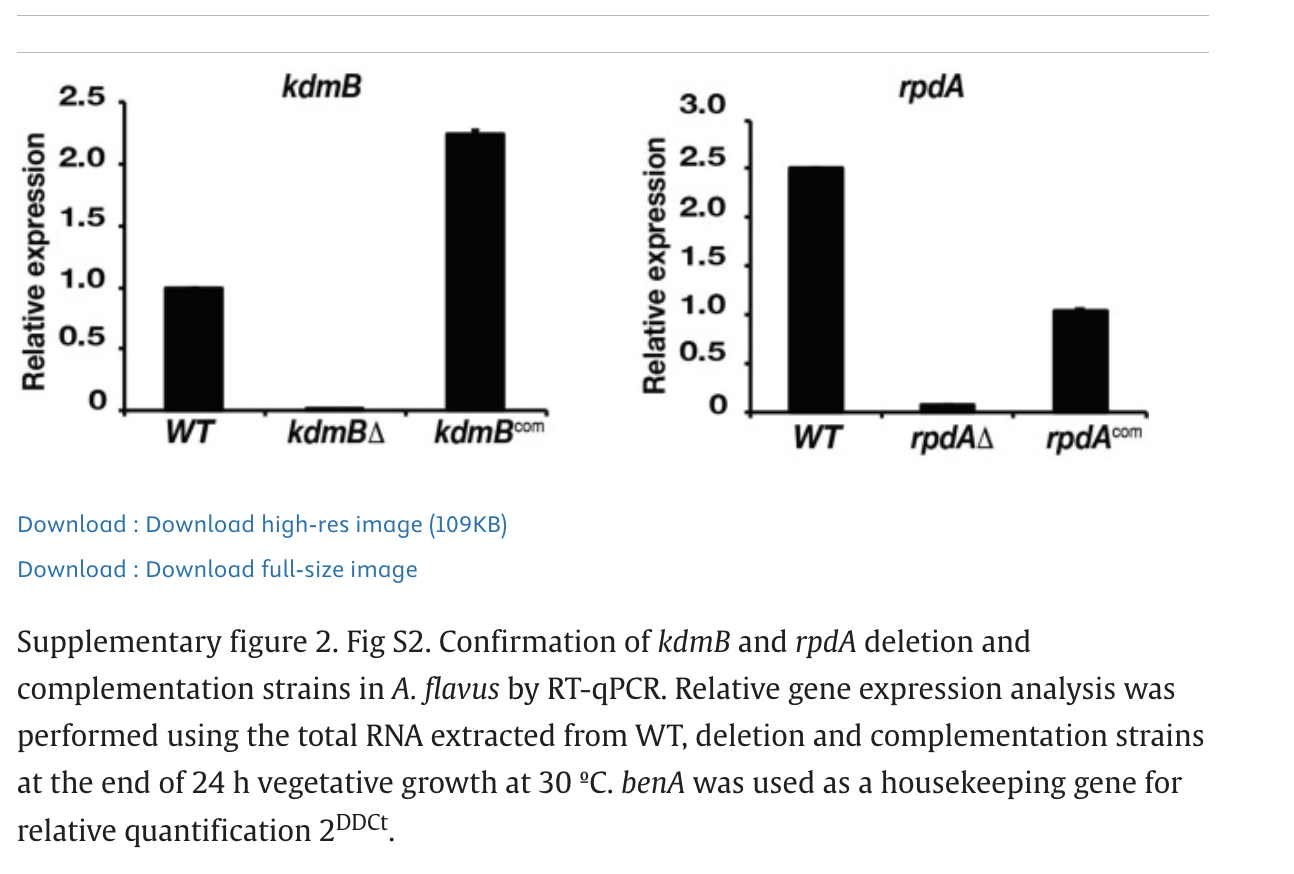

Supplement: Figure S2 [file NIHMS1938650-supplement-Figure_S2.png]

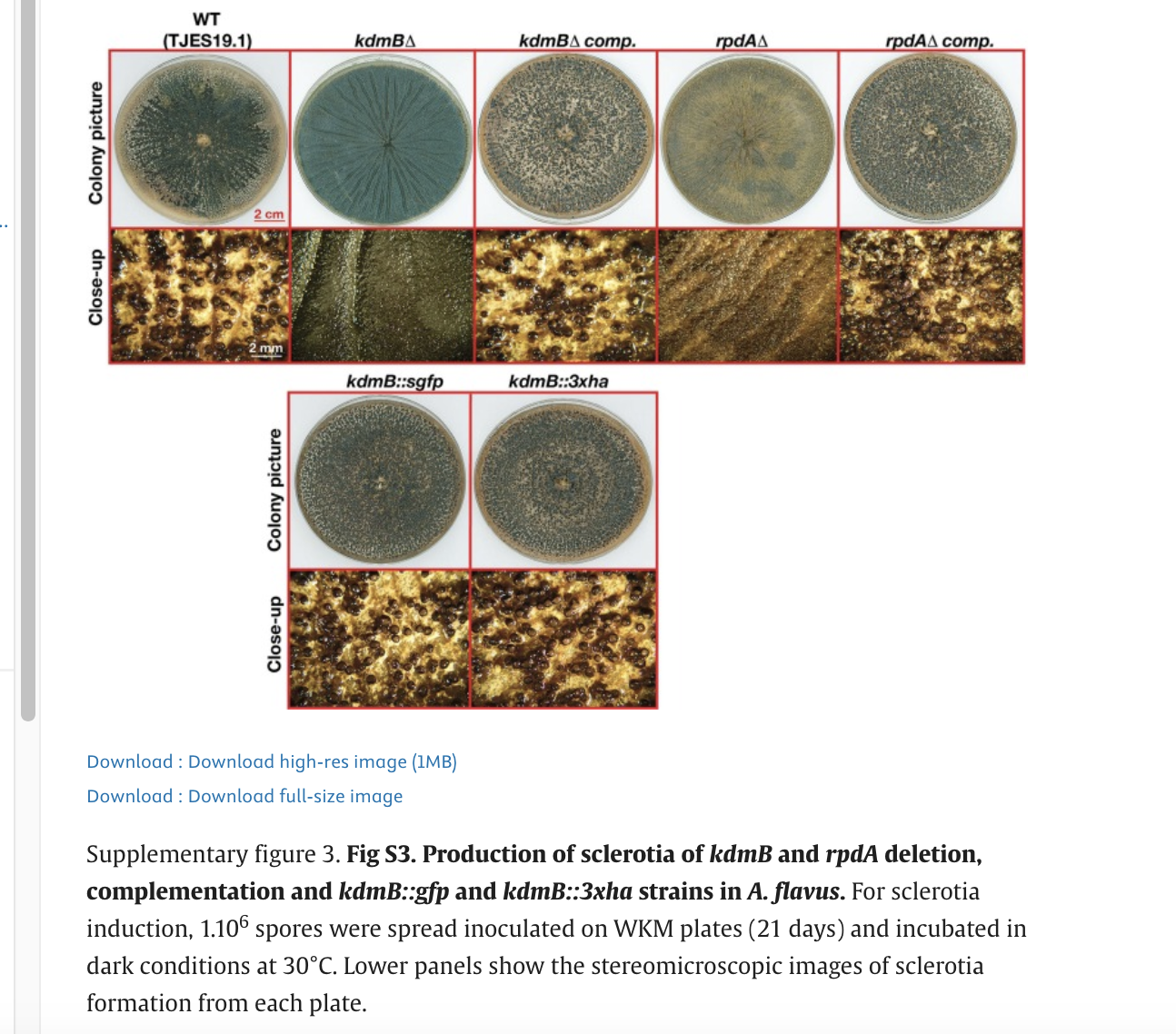

Supplement: Figure S3 [file NIHMS1938650-supplement-Figure_S3.png]
